# A context for the last Neandertals of interior Iberia: Los Casares cave revisited

**DOI:** 10.1371/journal.pone.0180823

**Published:** 2017-07-19

**Authors:** Manuel Alcaraz-Castaño, Javier Alcolea-González, Martin Kehl, Rosa-María Albert, Javier Baena-Preysler, Rodrigo de Balbín-Behrmann, Felipe Cuartero, Gloria Cuenca-Bescós, Fernando Jiménez-Barredo, José-Antonio López-Sáez, Raquel Piqué, David Rodríguez-Antón, José Yravedra, Gerd-Christian Weniger

**Affiliations:** 1 Neanderthal Museum, Mettmann, Germany; 2 Area of Prehistory, University of Alcalá, Alcalá de Henares, Spain; 3 Institute of Geography, University of Cologne, Cologne, Germany; 4 ERAAUB (Department of History and Achaeology), University of Barcelona, Barcelona, Spain; 5 ICREA, Barcelona, Spain; 6 Department of Prehistory and Archeology, Autonomous University of Madrid, Madrid, Spain; 7 Aragosaurus-IUCA, Department of Geosciences, University of Zaragoza, Zaragoza, Spain; 8 CENIEH (National Research Centre on Human Evolution), Burgos, Spain; 9 Archeobiology Research Group, History Institute, CCHS CSIC, Madrid, Spain; 10 Department of Prehistory, Autonomous University of Barcelona, Barcelona, Spain; 11 Department of Prehistory, Complutense University of Madrid, Madrid, Spain; Max Planck Institute for the Science of Human History, GERMANY

## Abstract

**Introduction and objectives:**

Although the Iberian Peninsula is a key area for understanding the Middle to Upper Paleolithic transition and the demise of the Neandertals, valuable evidence for these debates remains scarce and problematic in its interior regions. Sparse data supporting a late Neandertal persistence in the Iberian interior have been recently refuted and hence new evidence is needed to build new models on the timing and causes of Neandertal disappearance in inland Iberia and the whole peninsula. In this study we provide new evidence from Los Casares, a cave located in the highlands of the Spanish Meseta, where a Neandertal-associated Middle Paleolithic site was discovered and first excavated in the 1960’s. Our main objective is twofold: (1) provide an updated geoarcheological, paleoenvironmental and chronological framework for this site, and (2) discuss obtained results in the context of the time and nature of the last Neandertal presence in Iberia.

**Methods:**

We conducted new fieldwork in an interior chamber of Los Casares cave named ‘Seno A’. Our methods included micromorphology, sedimentology, radiocarbon dating, Uranium/Thorium dating, palinology, microfaunal analysis, anthracology, phytolith analysis, archeozoology and lithic technology. Here we present results on site formation processes, paleoenvironment and the chronological setting of the Neandertal occupation at Los Casares cave-Seno A.

**Results and discussion:**

The sediment sequence reveals a mostly *in situ* archeological deposit containing evidence of both Neandertal activity and carnivore action in level c, dated to 44,899–42,175 calendar years ago. This occupation occurred during a warm and humid interval of Marine Isotopic Stage 3, probably correlating with Greenland Interstadial 11, representing one of the latest occurrences of Neandertals in the Iberian interior. However, overlying layer b records a deterioration of local environments, thus providing a plausible explanation for the abandonment of the site, and perhaps for the total disappearance of Neandertals of the highlands of inland Iberia during subsequent Greenland Stadials 11 or 10, or even Heinrich Stadial 4. Since layer b provided very few signs of human activity and no reliable chronometric results, and given the scarce chronostratigrapic evidence recorded so far for this period in interior Iberia, this can only be taken as a working hypothesis to be tested with future research. Meanwhile, 42,000 calendar years ago remains the most plausible date for the abandonment of interior Iberia by Neandertals, possibly due to climate deterioration. Currently, a later survival of this human species in Iberia is limited to the southern coasts.

## Introduction

**Fig 1:**
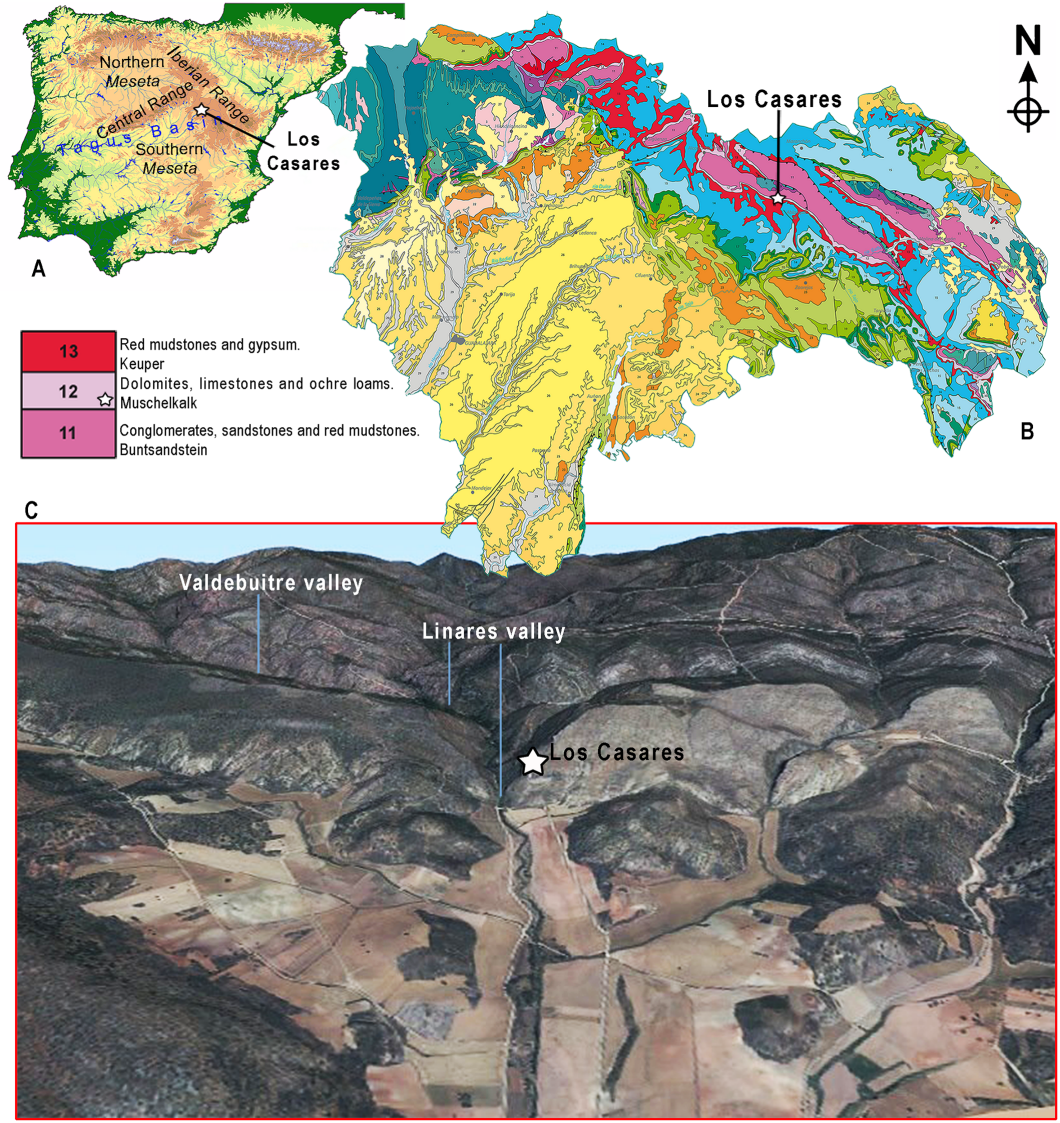
Regional setting of Los Casares. Location of Los Casares cave in the Iberian Peninsula (A) and in the Geologic map of the Guadalajara province (B). C: 3D view of Los Casares cave and the Linares and Valdebuitre valleys (Aerial photography and Digital Terrain Model—PNOA—from *Instituto Geográfico Nacional de España*).

The Iberian Peninsula has long been considered a crucial scenario for the Middle to Upper Paleolithic transition and the replacement of Neandertals by Modern Humans [1–6]. Since the late 1980’s, a key point on these discussions was the contention that Neandertals persisted in the center and south of Iberia until at least c. 36.7–34.5 ka cal BP [5], or even as late as *c*. 32–28 ka cal BP [4, 7]. This suggested that Neandertals and Modern Humans coexisted for several millennia, since Modern humans were presumably established in the northern regions of the peninsula from around 42–40 ka cal BP, or even earlier [2, 3, 8]. Here we focus on the interior lands of Iberia, which are dominated by the highlands of the Northern and Southern *Mesetas* divided by the Central System mountain range (Fig 1). Despite these inland territories had traditionally contributed with some chronometric evidence to the late survival model, reevaluation of the few sites involved has suggested however that no late Mousterian survival took place in inland Iberia [6, 9]. Since still few sites from this area have contributed to this discussion, new evidence is needed to build new models concerning the timing and causes of Neandertal disappearance in inland Iberia and the whole peninsula. A new interdisciplinary research project on Los Casares cave is aimed at moving forward in these scientific problems.

**Fig 2:**
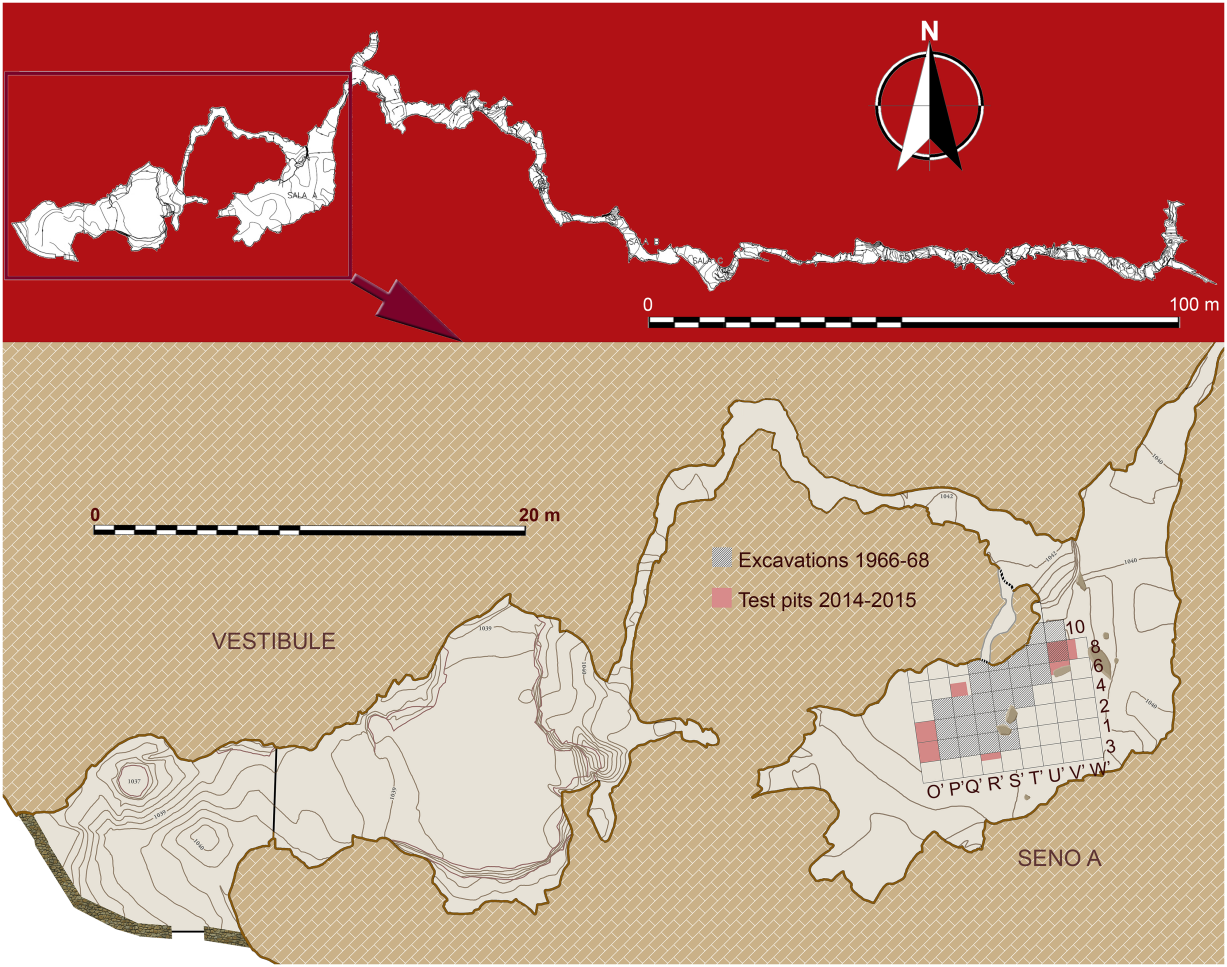
General plan of Los Casares cave showing Vestibule and Seno A areas.

Los Casares is a limestone cave located in the interior regions of the Iberian Peninsula (Spain). Its archeological potential is known since the late 19^th^ century, when first scientific explorations of the cavity pointed to the presence of bones and fossils in the floor and walls, and a historical site was discovered outdoors [10]. However, the relevance of this cave for the Paleolithic field became evident in the 1930’s, when its first Upper Paleolithic rock engravings were described by J. Cabré [11–13]. Later, between 1966 and 1968, a team directed by I. Barandiarán conducted the first systematic excavations in Los Casares, showing archeological deposits containing Middle Paleolithic assemblages in two different areas [14]. First deposit was located at the entrance hall of the cave, named *Vestíbulo* (Vestibule in Spanish), and it consisted of clayey sediments filling a short gallery at the bottom of this area (Fig 2). As reported by Barandiarán and recently observed by us, the presence of remnant sediments attached to the walls at different parts of this vestibule suggests that a now-destroyed larger deposit probably existed in this area. This is a very plausible hypothesis considering the long history of occupations and incursions documented both inside and outside the cavity from the Chalcolithic to Modern times, including its use as a sheep shelter during the 20^th^ century [14].

The second site was found in a deeper area of the cave, the so-called *Seno A*, an interior chamber where a larger deposit was discovered all along the place (Fig 2). Despite the area excavated here was of 21 square meters, Mousterian assemblages were scarcer, and recorded lithic artifacts were less than half in number than those found in the vestibule [15]. A Chalcolithic layer containing ceramics, lithics and faunal remains was also recorded at the top of the sequence of the Seno A site [14].

**Fig 3:**
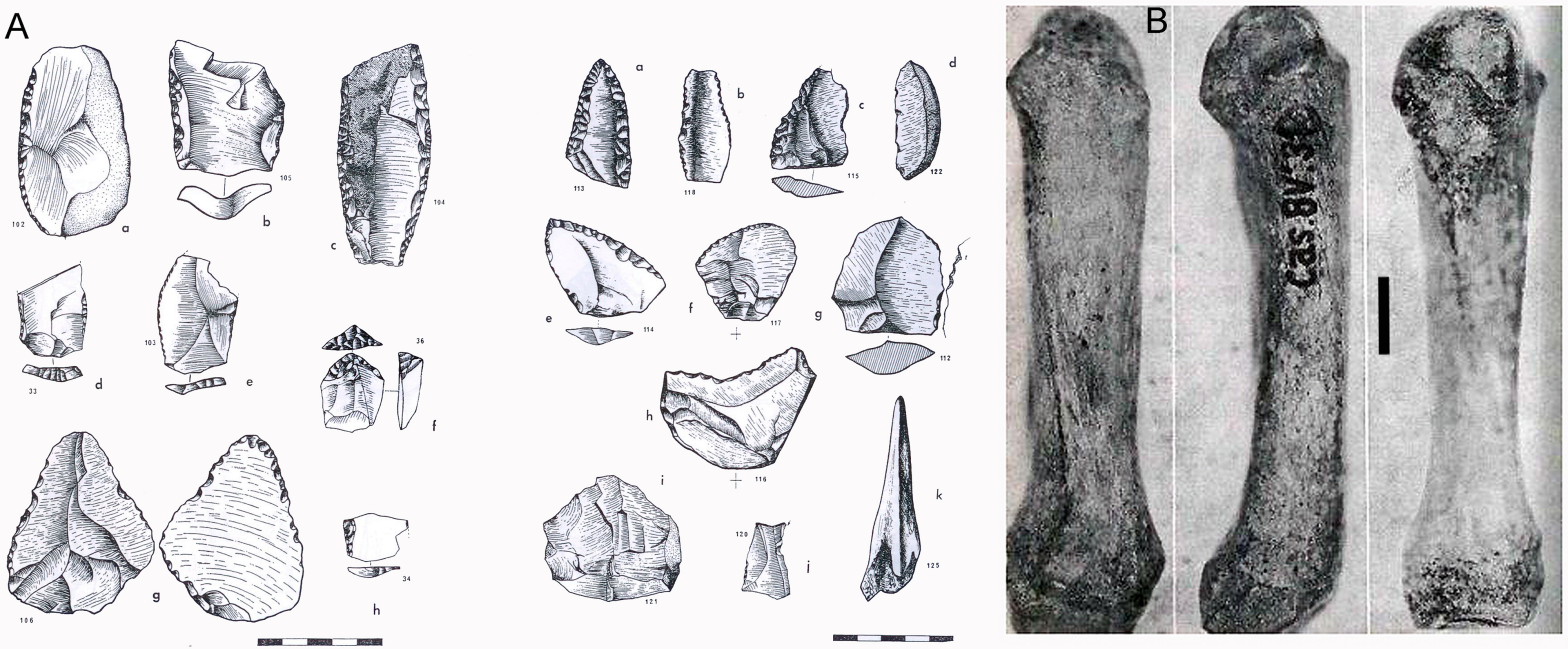
Main findings of the 1960’s excavations at Los Casares cave. A: Mousterian artefacts. All come from level c of Seno A except for numbers 33, 34 and 36 (modified after [14]). B: Neandertal metacarpal found in square 8V’ of Seno A (bar is 5 mm) (modified after [17]).

Archeological assemblages recovered at the two areas excavated in Los Casares not only included faunal and Mousterian lithic assemblages, but also a Neanderthal metacarpal bone found at the Seno A Middle Paleolithic layers (Fig 3B). This finding, together with the interesting nature of the lithic assemblages, composed of a high proportion of retouched tools, especially in the Seno A (Fig 3A), made Los Casares one of the most relevant sites for the study of the Middle Paleolithic in interior Iberia during the last quarter of the 20^th^ century. The scarcity of Late Pleistocene sites in these regions at the time, and the high quality of the monographic publication produced shortly after the excavations [14] were also key points stressing the relevance of this site for the study of the Iberian Middle Paleolithic. Furthermore, the presence of Upper Paleolithic rock art in such an interior region, far away from the classic Cantabrian and Mediterranean clusters, and including a striking proportion of anthropomorph figures [16], was also an indirect factor boosting the importance of Los Casares Middle Paleolithic site.

Despite this relevance, no scientific studies had been published on Los Casares Paleolithic record since the 1970’s, besides some partial analysis of the rock art [16], and some reviews of the faunal [18,19], and lithic assemblages excavated by Barandiarán [20]. Therefore, there was a significant scarcity of modern data hindering any attempt to integrate Los Casares evidence in current debates on the Middle Paleolithic settlement of Iberia and southwest Europe. Data on site formation processes were lacking, chronometric evidence was lacking, and paleoecological information was virtually absent. In sum, Los Casares Middle Paleolithic record was behind the times of current Paleolithic research.

In the summer of 2014 we started a new project aimed at the study of population dynamics and human-environment interactions during the Late Pleistocene in the central region of the Iberian Peninsula. A main factor driving this project was that record of this area was poorly known compared to the coastal regions, and in the case of the Middle Paleolithic this was especially evident concerning occupation of caves [21, 22]. Together with other two sites in the Guadalajara province (Spain), we selected Los Casares as a case study that could show relevant data on the Middle Paleolithic settlement of inland Iberia. It was our contention that Los Casares potential had been inexplicably neglected since the 1970’s, and therefore modern geoarcheological investigations could bring into light new insights for the understanding of Neandertal adaptations at this once key site of the Iberian Middle Paleolithic.

Overall, our main objective was to gain a better geoarcheological understanding of Los Casares Middle Paleolithic site in order to contribute to current debates on the Neanderthal settlement of inland Iberia. Among these debates, the long-claimed Mousterian late survival in the central and southern areas of the peninsula [23], and the nature of human adaptations to the harsh environments of the upland regions of the Spanish plateau [24], were the most relevant. Both are currently under dispute [4–6, 9, 25–31].

Here we publish results of an interdisciplinary geoarcheological investigation of Los Casares-Seno A site, where we conducted new field and laboratory works. We undertook micromorphological, sedimentological and taphonomic analyses aimed at deciphering site formation processes, we performed radiocarbon and U/Th dating for setting up a chronological framework, we conducted palynological, anthracological, micromammal, phytolith and sedimentological analyses for elucidating environmental and climatic settings, and we studied lithic and faunal assemblages for discussing Neandertal techno-economic behaviours. Integration of results obtained by all these methods depicts an ecological and chronological context for the last Neandertals living in this interior area of the Iberian Peninsula.

## Regional and local setting

**Fig 4:**
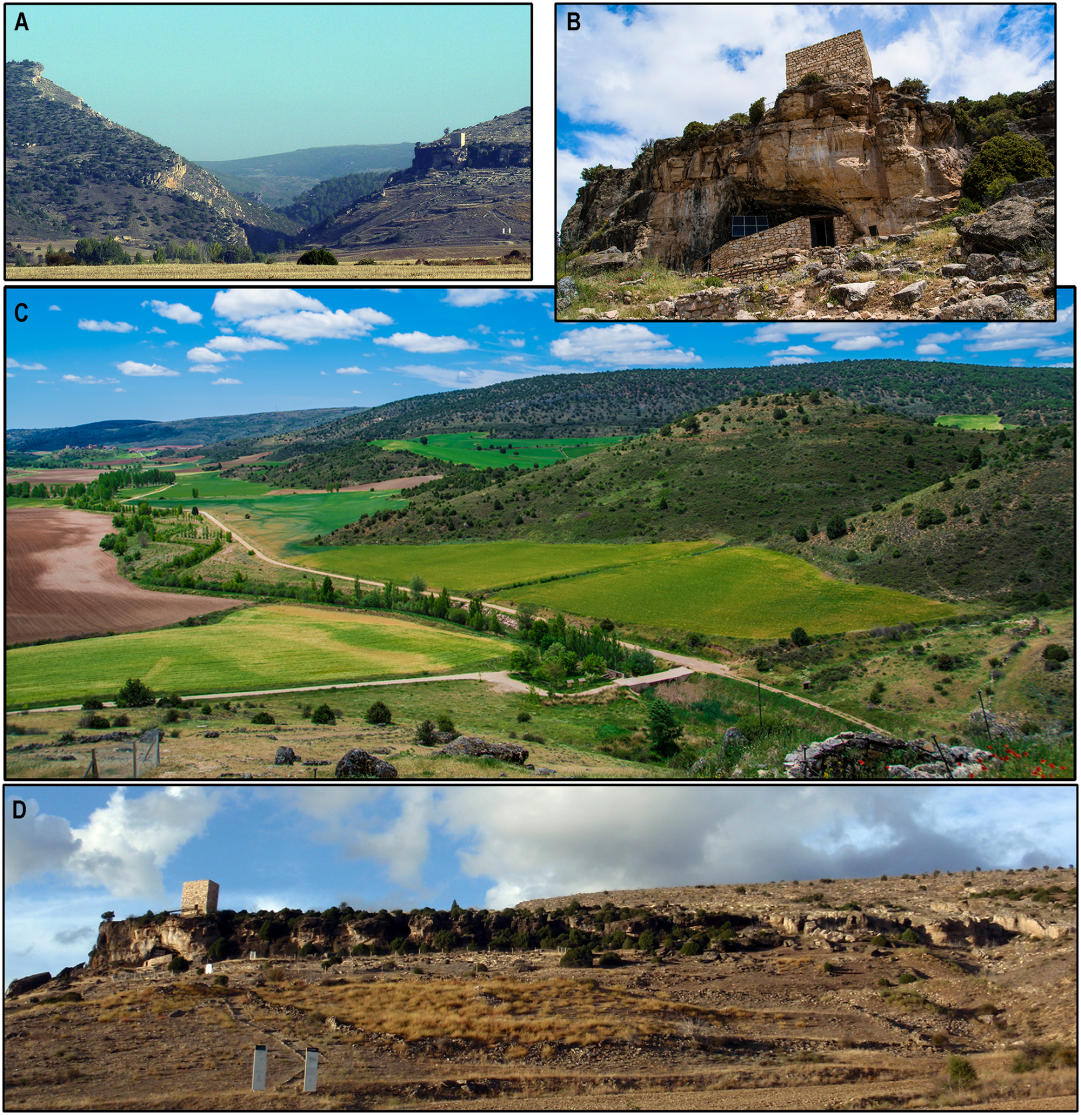
Local setting of Los Casares. A: Los Casares cave and the narrowing of the Linares River downstream of the ‘Milagros’ valley. B: Entrance to the cavity. C: General view of Los Casares cave from the south. D: View of the Linares River valley from the cave’s entrance.

**Fig 5:**
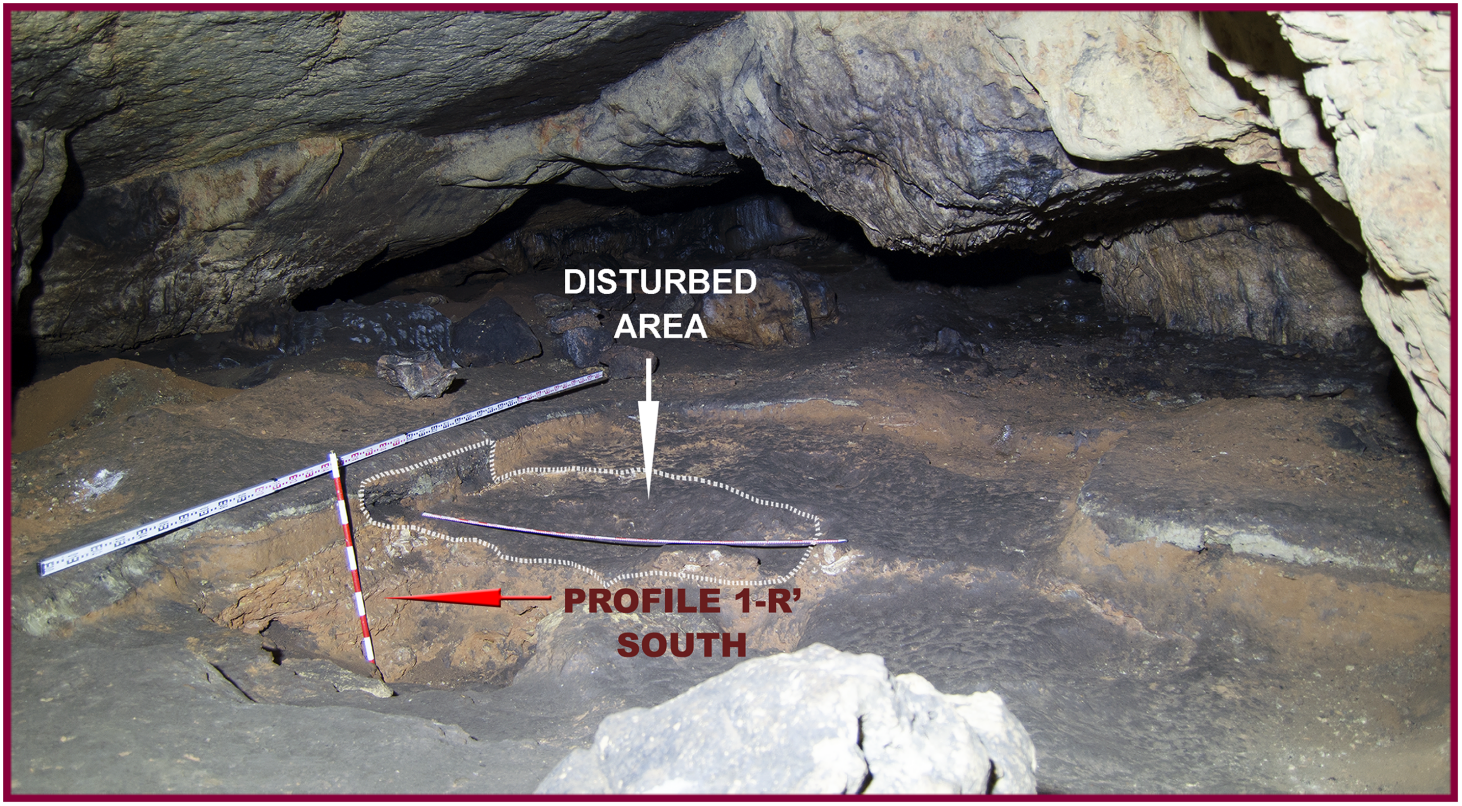
View of the Seno A chamber prior to our fieldwork. Profile 1R’ South produced by the 1960’s excavations and adjacent disturbed area are shown.

Los Casares is a southwest-oriented cave eroded in a limestone-dolomite cliff corresponding to the Muschelkalk lithostratigraphic unit (Middle Triassic) (Fig 1B). It is in the environs of La Riba de Saelices village (Guadalajara Province, Spain), located in the moorlands of Sigüenza and Molina de Aragón belonging to the Iberian Range, at the northern fringe of the Southern Meseta at about 1040–1060 m asl (40° 56' 22'' N, 2° 17' 31'' W, Datum ETRS89) (Fig 1A). The cave entrance and vestibule are situated at about 40 m above the southward widening valley floor of the Linares River (Upper Tagus basin), on its left bank (Figs 1C and 4). Los Casares is a diaclase cave, with few and small lateral galleries, and with a total length of about 264 m from West to East (Fig 1A). When studying cave art, J. Cabré [11] defined three different concavities within the main passage that he called, from outside to inside, “*Seno A*”, “*Seno B*” and “*Seno C*”. Seno A is found after leaving the vestibule and passing a narrow gallery about 20 m long (Fig 1B). It consists of an east-west trending cavity with a complex topography, about 20 m long, 10 m wide and up to 4 m high. The archeological deposit object of this study is found all along the Seno A chamber (Fig 5)

## Materials and methods

### Permits and repositories

All necessary permits were obtained for the described study, which complied with all relevant regulations. Field and laboratory works at Los Casares cave were authorized by the *Dirección General de Cultura de la Junta de Comunidades de Castilla–La Mancha* (Spain) (Exp.: 14.0955-P1 and Exp.: 14.0955-P3). Study of lithic and faunal remains curated at the *Museo Arqueológico Nacional* (Madrid, Spain) was authorized by the Prehistory Department of this museum.

The Los Casares lithic and faunal assemblages excavated in 2014–2015 are housed in the *Museo de Guadalajara* (Guadalajara, Spain). Assemblages from the 1960’s excavations are housed at *Museo Arqueológico Nacional* (Madrid, Spain). Both repositories are accessible for all researchers.

### Fieldwork: Excavation, stratigraphy and sampling

**Fig 6:**
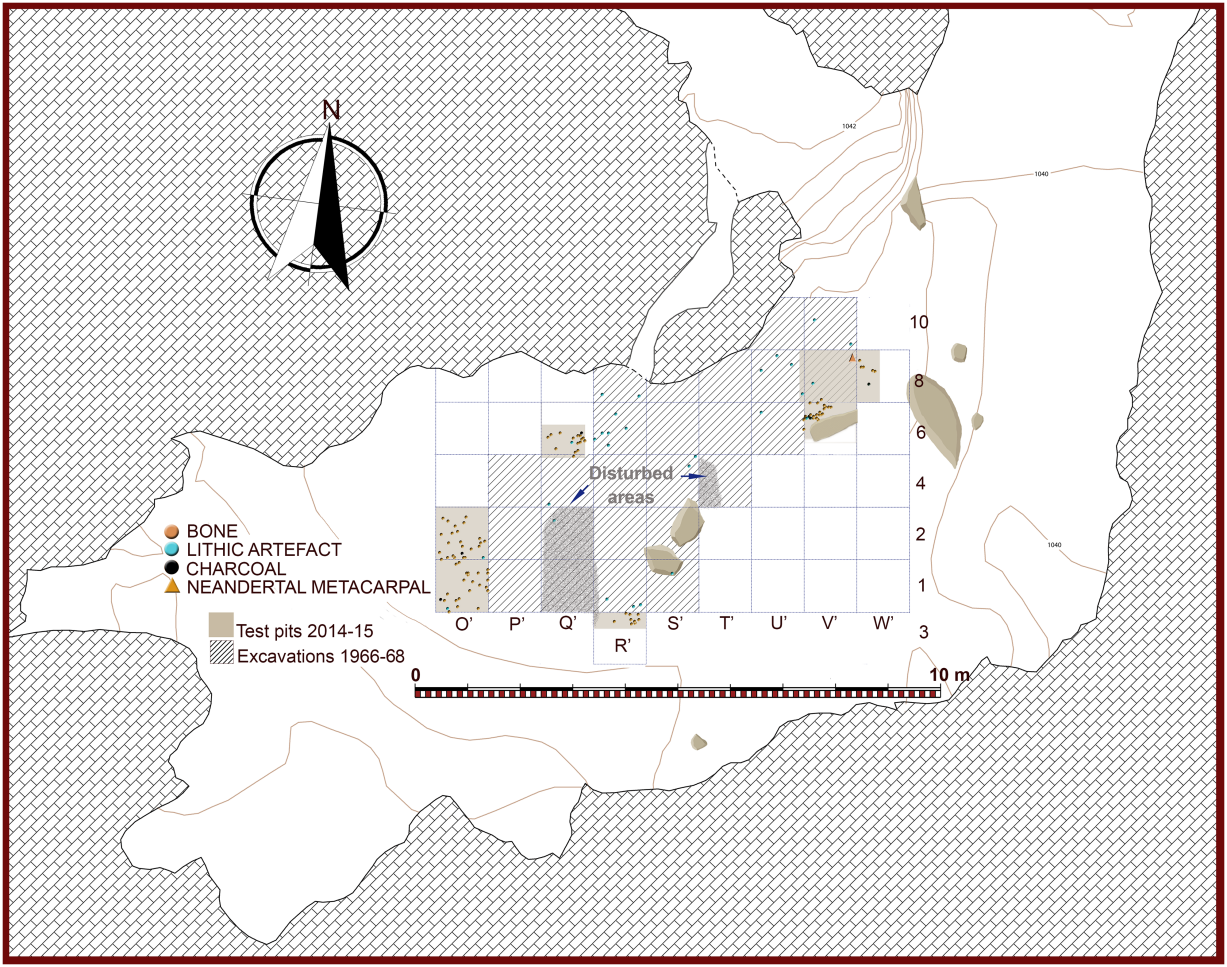
Plan of the Seno A showing excavated areas in the 1960’s and in 2014–2015. For the latter, archeological assemblages from level c are plotted. However, for the 1960’s excavations only lithic artefacts are plotted (after [14]), since no spatial recording of bone or charcoals were done.

**Fig 7:**
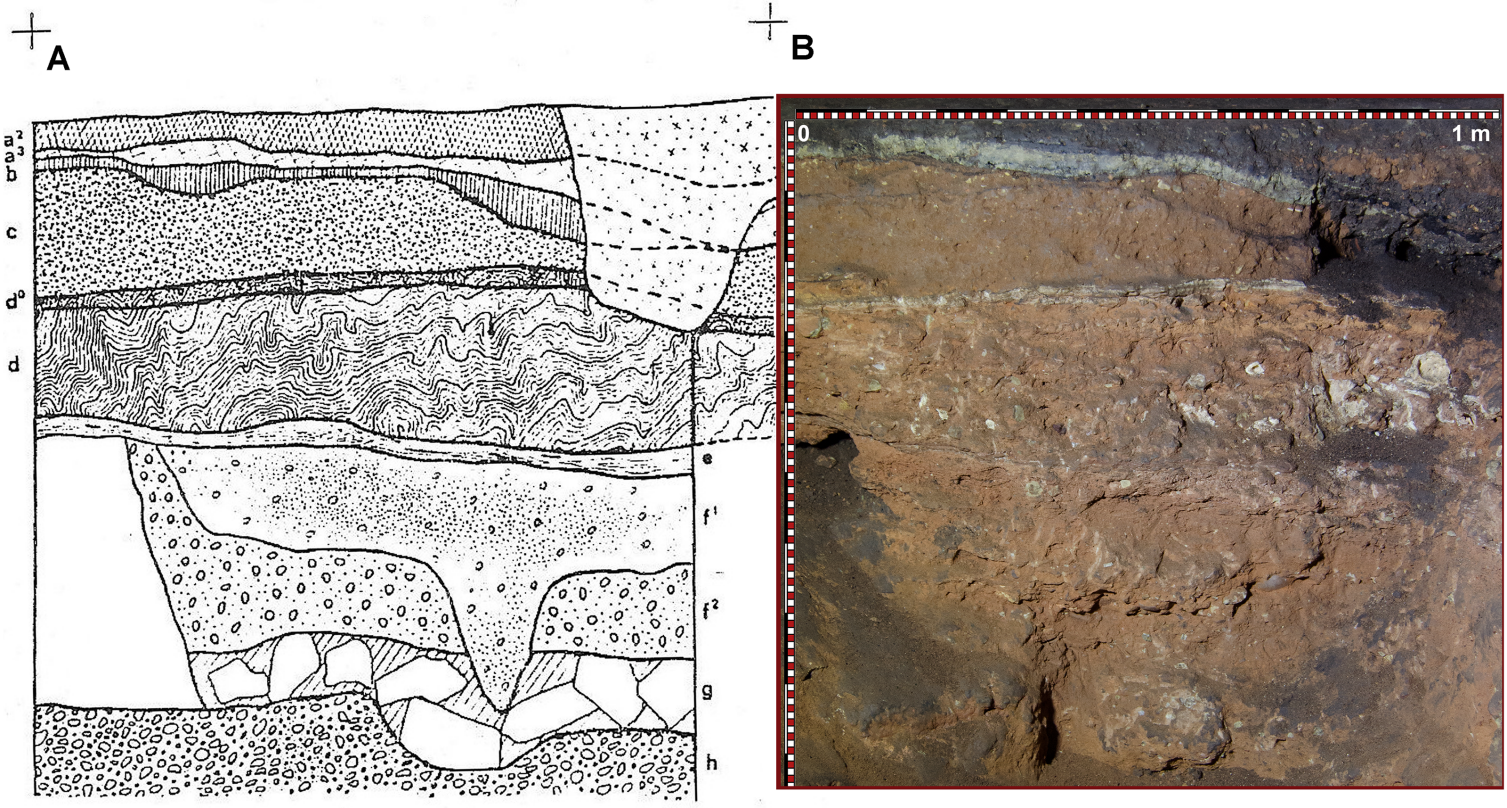
Stratigraphic sequence excavated in the 1960’s. A: Stratigraphy described by Barandiarán in profile 1-R’ South (modified after [14]). B: Uncleaned profile 1-R’ South prior to our fieldwork. Post-depositional disturbance at the upper western part was easily recognized.

Previous work at the Seno A conducted by I. Barandiarán in the 1960’s [14] consisted of the archeological excavation of 21 square meters (Fig 6). A stratigraphic sequence of grey-greenish and reddish-brown Holocene and Pleistocene sediments divided in eight sedimentary layers, from level “a” to level “h”, was described in a test pit reaching a total depth of about 1 m below the modern cave floor. However, in most of the excavated area only the first three layers, subsequently divided in different sub-levels in some places, were reached, at a total depth of 30–40 cm (Fig 5). Archeological assemblages were found at layer a3, where ceramics, lithics and faunal remains were assigned to the Chalcolithic and Early Bronze age. While layer b was described as sterile, Middle Paleolithic assemblages, including a Neandertal metacarpal, were identified at level c. Below this layer, a flowstone was identified as level d0, followed by a heavily cemented layer d, very rich in animal bones but lacking any artefacts. Layer e was identified as a stalagmitic crust, and lower layers f1, f2, g and h were considered archeologically sterile (Fig 7). Although Barandiarán judged that most of the deposit at the Seno A was preserved in situ, he acknowledged the presence of post-depositional disturbance at some areas, mainly related to clandestine excavations (Figs 5 and 6).

The southern profile produced by the above-mentioned test-pit, located in square 1-R’, was still preserved at the site in 2014, hence allowing a direct inspection. We thus identified the sequence published by Barandiarán, as well as a good example of one of the post-depositional disturbance features referred by this author: a clandestine pit in the western part of the profile (Fig 7). Since this profile offered an excellent platform for starting new excavation works, our first task consisted on its rejuvenation 25 cm to the south. We thus produced a new profile in square 3-R’, where the old sequence could be contrasted more in detail. In doing so, and in our general fieldwork at the site, we used grid and datum established by Barandiarán’s team in the 1960’s (Fig 6). During the two campaigns conducted in 2014 and 2015 we excavated a total extension of slightly more than 5 square meters, divided in four different areas located at the perimeter of the 1960’s excavation. The first area consisted of the rejuvenation of profile 3-R’ South, the second was square 6-Q’, the third included squares 1-O’ and 2-O’, and the fourth consisted of squares 6-V’, 8-V’ and 8W’. All these areas were relatively close and hence stratigraphic correlations between them were feasible (Fig 6).

Fieldwork methodology followed the excavation of natural levels, which were divided in artificial layers of 2–3 cm when needed. Both these layers and every archeological object or feature larger than 2 cm (lithics, bones, charcoal fragments and human-made structures) were three-dimensionally recorded using a Total Station, and orientation and dip of elongated lithic and bone products were registered. All excavation layers were digitally photographed before collecting archeological assemblages, which were also pictured in detail in special cases (i.e. concentration of objects or relevant lithic artefacts). Stratigraphic profiles and some excavation plans were also hand-drawn. Every square meter was divided in sectors of 33 sq cm and sediment was packed accordingly. This sediment was later floated and wet-screened at 2 and 1 mm mesh sieves at the Laboratory of Prehistory of the University of Alcalá, where most of the lagomorphs, micromammals, charcoals and lithic debris were acquired. Samples for micromorphology, pollen, phytolith and Uranium/Thorium dating of flowstone were also collected during excavation.

### Micromorphology

For micromorphology, five sediment monoliths were extracted from the upper part of the sequence at different excavation areas, covering layers r to c2. Sampling of the heavily cemented sediments of level d was not successful. Since sediments of the upper sequence were very brittle, the samples were reinforced with gypsum bandages before extraction. The monoliths 1 and 2 were taken from the south profile of square 3-R’, monoliths 3 and 4 from the south profile of square 1-O’ and monolith 5 from the north profile of 8-W’ (Fig 6). While monolith 3 was kept for reference, the other monoliths were used for preparation of three uncovered thin sections each (maximum size 60 mm x 80 mm), using methods described by Beckmann [32]. The thin sections were scanned with a flatbed scanner using polarizing foil for inspection at low magnification. A petrographic microscope was used to study sediment composition and fabric at magnifications of 12- to 500-fold using plane polarized light (PPL) or crossed polarizers. In addition, oblique incident light (OIL) was used in some cases. The description of thin sections followed terminology suggested by Stoops [33]. All lab works were conducted at the Institute of Geography of the University of Cologne.

### Uranium/Thorium dating

**Fig 8:**
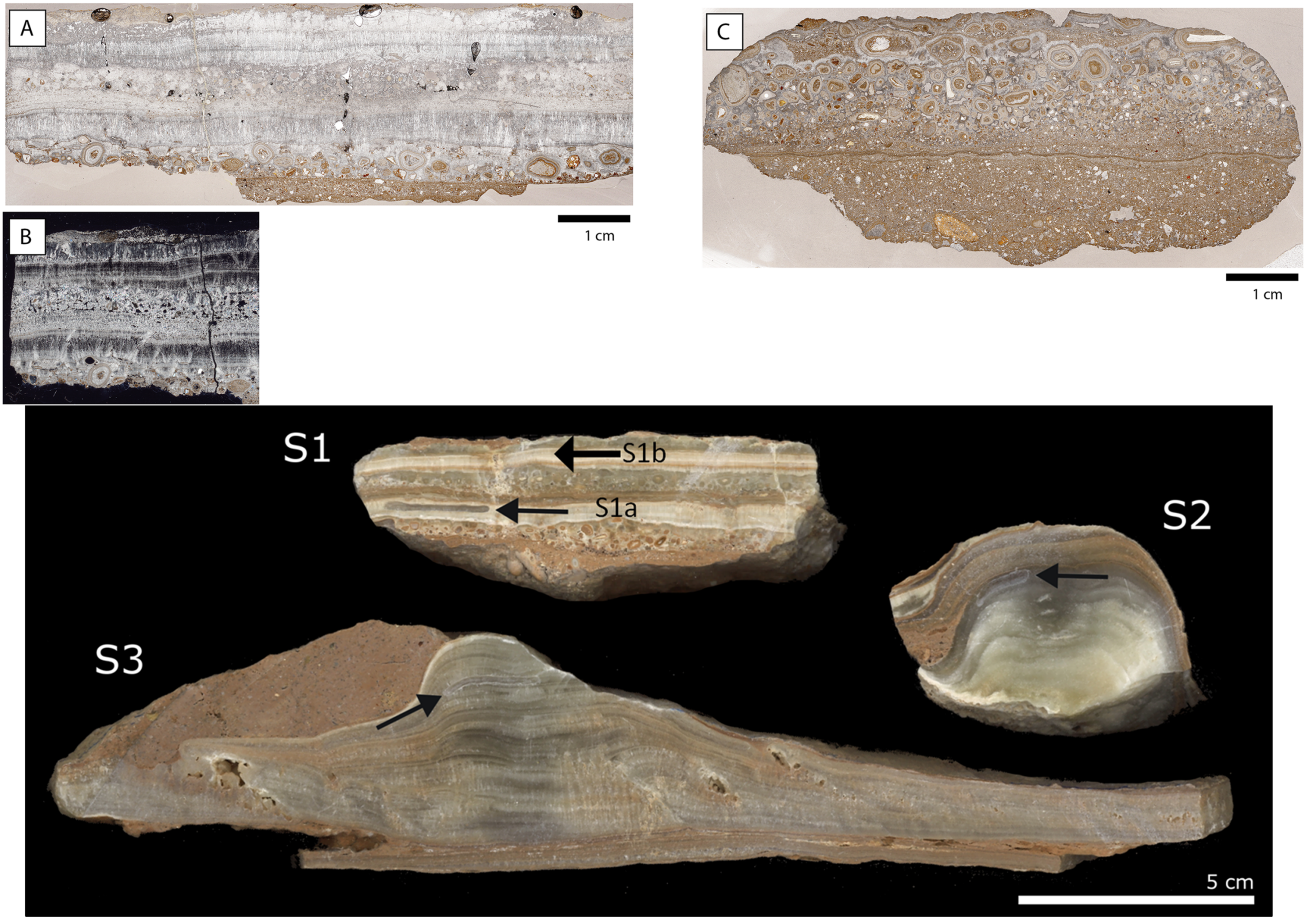
Thin section scans of the flowstone of d0 at profile 3R under plain polarized light (A) and crossed polarizers (B). C shows a scan of a calcite incrusted layer of d0 extracted from square 6-Q’. The lower image shows the three speleothem samples selected for Uranium/Thorium dating (D). Note the small sampling areas.

Speleothem samples from level d0 were extracted with hammer and chisel from different areas of the excavation. Two samples were extracted from square 3-R’, one from square 6-Q’ and two more from square 2-O’ (Fig 6). All samples were flowstones presenting a good degree of crystallisation except for that collected at square 6-Q’. The latter was better defined as a calcite incrusted layer or stalagmitic crust (Fig 8).

**Fig 9:**
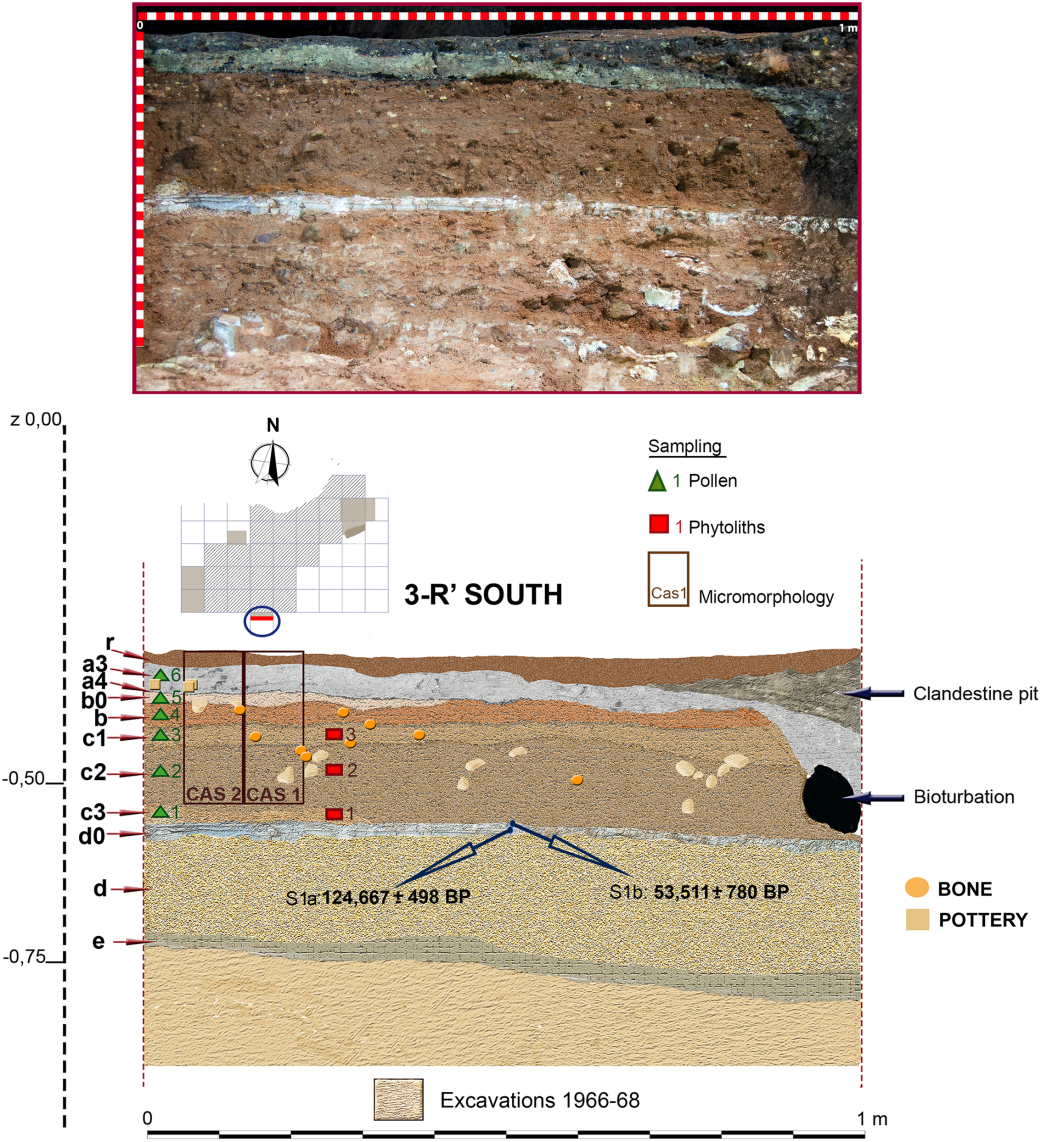
Stratigraphic sequence and vertical distribution of items recorded in profile 3-R’. Stratigraphic position of samples for pollen, phytolith, micromorphology and Uranium/Thorium dating is shown.

**Fig 10:**
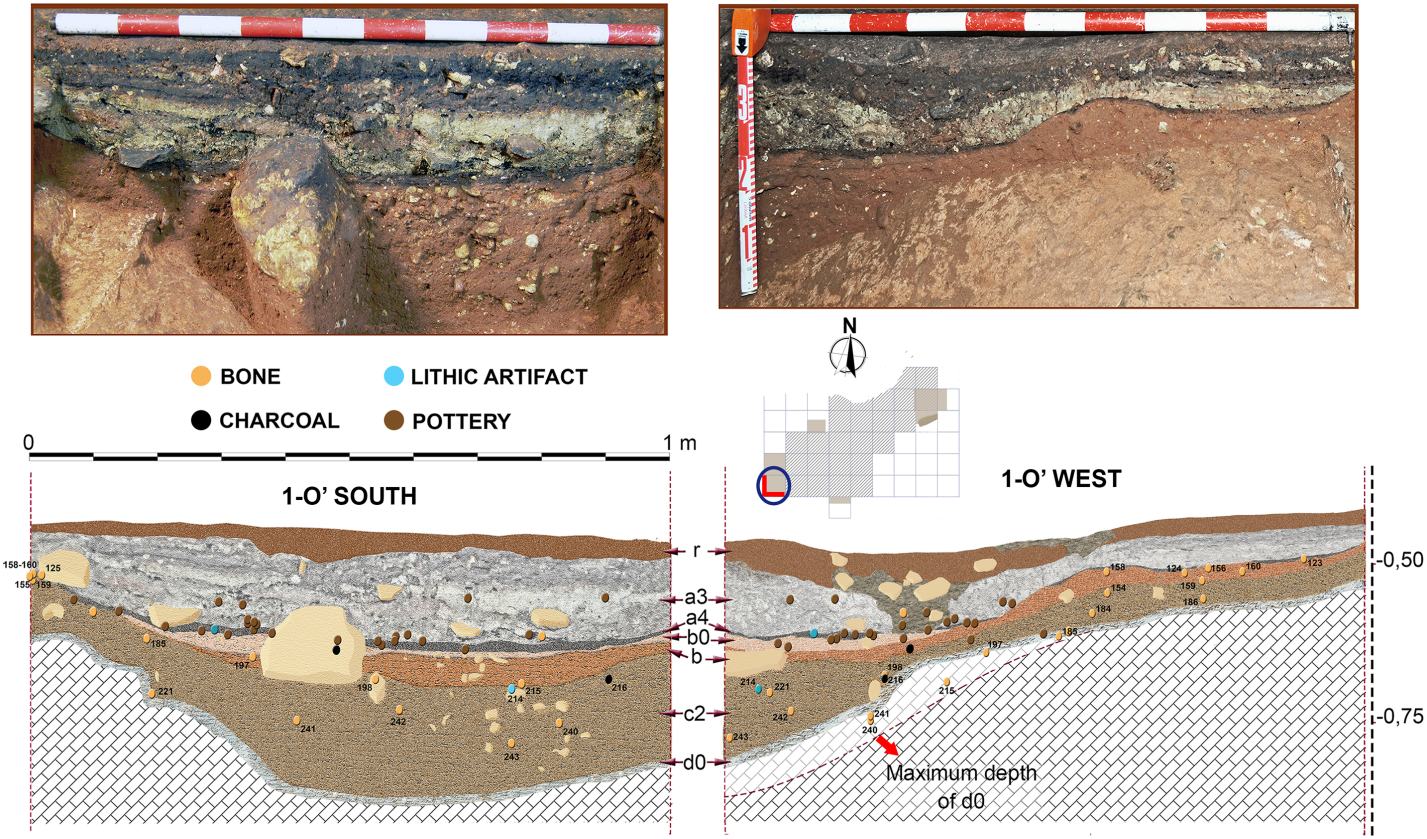
Stratigraphic sequence and vertical distribution of items recorded in profile 2-O’ West. Stratigraphic position of samples for radiocarbon and Uranium/Thorium dating is shown.

Three of these samples, S1, S2 and S3, were processed in the Laboratory of Uranium Series at the CENIEH (Burgos, Spain). S1 was taken at square 3-R’, while S2 and S3 were collected at 2-O’. Despite lateral variation of stratigraphic sequence all along the deposit, “z” values (deepness) of these samples were very similar (Figs 9 and 10). At the lab, smaller samples were extracted using a hand drill and 0.8mm tungsten carbide drill bits. From S1 two sub-samples were taken: S1a and S1b (Fig 8). After weighing, around 50mg precisely measured samples were dissolved in HNO_3_ and digested in several steps involving HNO_3_, H_2_O_2_ and HCl treatments. Uranium and thorium were then separated and their solutions purified by using two ion exchange resin column steps (AG1X8 and UTEVA) following [34 and 35] sample preparation protocols.

Uranium and thorium isotope measurements were performed using a Multicollector Inductively Coupled Plasma Mass Spectrometer (MC-ICPMS, Thermo NEPTUNE) with a membrane desolvator Aridus 2, X-cones and Jet interface pumping to increase the signal. Nebulizer consisted on a PFA microtip calibrated for 50μl/min. Measurement protocols followed can be found in [34 and 35]. Spike working solution used was made of a 1M ultrapure HNO_3_ gravimetrically produced solutions of NIST 4328c for ^229^Th, and IRMM3636a for ^236^U. Standard solutions of those reference materials were used for standard sample bracketing. Both standard solutions and samples were checked for U, Th elemental concentration by ICPOES (PerkinElmer 5300DV) or ICPHRMS (ELEMENT XR).

^230^Th/U ages were obtained by solving numerically or graphically the corresponding equation [36, 37]. Corrected ages were calculated assuming an initial isotope ratio ^230^Th/^232^Th = 4.4 x 10^−6^. Decay values considered for the equation were those found in [38].

Sample S1 showed some layers where oncolite accumulation is predominant. Since this is usually related to bioturbations and could indicate open system conditions, attempts to date those and surrounding areas would lead to obtain wrong U series dates. However, although the lower layer sampled here (S1a) showed a high concentration of ^232^Th (151ng·g^-1^), ^230^Th/^232^Th ratio was high and hence U/Th dating was performed. On the other hand, sample S1b was collected from a series of well laminated layers of calcite formed after the upper oncolite deposits at the top part of the speleothem (Fig 8). This area was wide enough to obtain enough sample amounts suitable for U series dating over the brown-reddish narrow layer. Concentration of ^232^Th (151ng·g^-1^) was lower in this sample.

### Radiocarbon dating

From level b, two charcoal fragments and two faunal bones were selected for radiocarbon dating. From level c, one charcoal fragment and three bones were selected. All these samples (n = 8) were submitted to the CologneAMS centre at the University of Cologne. None of the bones were anthropogenically modified, since no clear cut-marks or any other anthropogenic sign were identified in the faunal assemblages collected at the 2014–2015 excavations. Measurement of the five bones failed due to no collagen yield. Therefore, only three radiocarbon measurements on charcoals could be obtained out of the eight samples tried.

Charcoal samples were first identified to taxon and then AAA (Acid-Alkali-Acid extraction) processed according to sample preparation described by Rethemeyer et al. [39]. Dating results will be presented below in conventional radiocarbon years and as calibrated ages BP using OxCal 4.3 [40] and IntCal13 [41].

### Pollen analysis

Six sediment samples of 5 square cm were extracted from profile 3-R’ (Fig 9) during the 2014 excavation season following standardized techniques for archeological sites as described in [42 and 43]. Five of them (excluding that corresponding to the Chalcolithic a3 layer) were prepared for pollen analysis at the CSIC labs (Madrid) using standard HCl sieving, HF and density separation techniques (solution density 1.9) outlined in Burjachs et al. [42]. After processing, the samples were suspended in glycerin prior to being mounted on slides. Counting was undertaken using a Nikon Elipse 50i light microscope at x400 magnification until a sum of 300 total land pollen (TLP) had been achieved, excluding Aster type, Cardueae and Cichorioideae with possible zoophily [43]. Pollens were identified with the aid of the key in Moore et al. [44], photographs in Reille [45] and modern reference material. *Pinus nigra*-type pollen grains were categorised following measurements in Desprat et al. [46]. Pollen diagrams were constructed using TILIA 2.0 and TGView software [47] and percentages were based upon the TLP.

CONISS [48] was used to assist with the biostratigraphic zonation of the pollen diagram in Local Pollen Assemblage Zones (LPAZs) according to the dissimilarity matrix of Euclidean distances and squared root transformed of percentage data. Ordination by principal components analysis (PCA) was performed using CANOCO 4.5 software, as a linear interpretation method for the fossil dataset since a previously detrended correspondence analysis (DCA) pointed to a linear response of species data to environmental gradients [49]. Samples were square-root transformed for a better comparability. In the PCA scatter plot, pollen taxa are shown as distance biplot arrows, and the direction of the arrow indicates the direction in which the values of the corresponding taxa increases.

### Microfaunal analysis

Fossils of small vertebrates were collected by hand during fieldwork seasons of 2014 and 2015, and by wet-screening of sediments at 2 and 1 mm mesh sieves at the Laboratory of Prehistory of the University of Alcalá during 2015 and 2016. Microfaunal remains were found all through the excavated areas in the Seno A, and in all stratigraphic layers besides a2, a3 and a4.

A total of 109 plastic bags filled with sediments were wet-screened at the laboratory. The resulting concentrates were examined by naked eye as well as by optical microscopes. Microfauna and other small fragments of large fossils were separated by picking up the elements. The resulted 109 collections of fossils were then sent to the Department of Earth Sciences of the University of Zaragoza, where assemblages were examined, photographed and stored.

A total of 102 out of the 109 analyzed samples showed fossil remains that were classified to the species level. Additional washing with micro-mesh techniques and 10% HCl, and/or H_2_O_2_ was used when the surfaces of the molars, especially the enamel-dentine junction on the occlusal surface, was covered with particles of sediment that impeded the visual analysis. This anatomical region is needed pristine for the good classification and the morphometric analysis of small mammals. Drawings were made after photographs taken with an Olympus SZ61 microscope with a camera attached to it. Images and measurements were taken with the camera and the LCMicro software provided for the Olympus equipment.

Classification of small mammals into species was based on the morphology and biometry of the occlusal surface of the molars, following general criteria of systematic paleontology [50–54]. In each sample, we counted the number of skeletal elements, mainly dentition, and calculated the minimum number of individuals (MNI). The input for computing the MNI are the diagnostic skeletal parts that represents one individual of the species in a sample; i.e. two left lower first molars (m1) of a given species represents two individuals, whereas two m1, one left and one right represents one individual.

### Charcoal analysis

Charcoal remains were sampled by hand during fieldwork and by flotation in the Laboratory of Prehistory of the University of Alcalá. A total of 44 fragments of carbonized wood from levels b, c and a5 have been studied in the Archeological Analysis Service of the Autonomous University of Barcelona. Identification of taxa was carried out following standard procedures. The anatomical patterns of each wood species were observed along three sections (transversal, longitudinal tangential, and longitudinal radial) using a reflected light microscope equipped with light field/dark field and objectives of 4x, 10x, 20x and 40x. Archeological samples have been compared with modern woods as well as with wood anatomy atlases [55].

### Phytolith analysis

Three sediment samples from profile 3-R’ were collected during the 2014 excavation season for phytolith analyses. Samples were processed at the Laboratory for Palaeoecology and Plant Palaeoeconomy–BioGeoLab (IMF–CSIC, Barcelona). The samples corresponded to sub-layers c1, c2 and c3 of level c (Fig 9).

The extraction process follows the methods proposed by Madella *et al*., [56]. Samples were oven-dried at 80°C for 24 hours and screened with a 1 mm mesh to remove sands larger than 1000 μm. 15 millilitres of a 5% solution of hydrochloric acid (HCl) was added for 3 hours to dissolve carbonate minerals and after the reaction ceased the acid was removed by centrifuging the sample at 2000 RPM in 50 ml tubes. Samples were then de-flocculated with a 5% weight solution of sodium hexametaphosphate ((NaPO_3_)_6_) for 36 hours, in order to disaggregate and remove the clays, and centrifuged at 2000 rpm with distillate water. 30% solution of hydrogen peroxide was added to samples for 3 hours to remove organic material. The resulting sediment, what is the Acid Insoluble Fraction (AIF) [57], was then oven-dried at 60°C. A 10 ml of sodium polytungstate solution (SPT) (Na_6_(H_2_W_12_O_40_)H_2_O) with a density of 2.35 g/cm^3^ was added to separate siliceous minerals by density, vortexed and centrifuged for 3 min at 2000 rpm. The supernatant, where phytoliths are, was recovered with a Pasteur pipette discarding the heaviest fraction and oven-dried at 40°C for 72 hours. 5μg per sample were finally placed on microscope slides, mixed with *Entellan* and covered with 20 x 20 mm cover slips for examination under the petrographic microscope (Olympus BX43) at 400x. The analysis of phytoliths was conducted during 2016 in the Archeological Analysis Service of the Autonomous University of Barcelona.

Morphological and taxonomical identification of phytoliths was based on standard literature [58–62], including the PhytCore online data base [63]. Other references from paleoclimatic and ecological analogues, such as the areas from the Mediterranean-Alpine climatic zones [64], as well as references from sedimentological and paleoenvironmental contexts from the Iberian Peninsula albeit from different chronologies (MIS 4–5), were also consulted [65]. Special attention was paid to the presence of weathered phytoliths due to postdepositional processes [66, 67].

### Archeozoology and taphonomy

52 faunal remains from level b, 1,318 from level c and 85 from level d were subject to zooarcheological and taphonomic analyses at the Prehistory Department of the Complutense University of Madrid. No human remains where identified despite close inspection by paleoanthropologists. While all remains from levels b and d corresponded to the 2014–2015 fieldworks, assemblages from level c included 515 remains from the 1960’s excavations [19, 68], housed at the Museo Arqueológico Nacional (Madrid), and 803 from the recent ones. Since it was hypothesized that an archeological selection of bone fragments could have be done during the 1960’s fieldworks [19], most probably due to a lack of wet-screening, data from the two assemblages were recorded separately in a first stage of research.

Studied remains included both identifiable and unidentifiable fragments and the taxonomical identification was based on reference materials. When the identification was not feasible, epiphyses, axial and shaft fragments were assigned to three animal weight/size classes: 1) small-sized carcasses, <100 kg (e.g. *Capra pyrenaica*, *Rupicapra rupicapra*), 2) medium-sized carcasses, >100–300 kg (e.g. *Cervus elaphus*) and 3) large-sized carcasses, >300 kg (e.g. Equus ferus, Bos primigenius).

The estimation of NISP (Number of Identified Specimens) and MNI (Minimum Number of Individuals) was used to determine the most appropriate features of the faunal taxonomic distribution. NISP follows Lyman’s synthesis [69] and MNI is based on Brain’s model [70] that includes bone laterality -right/left- and animal age. Furthermore, skeletal profiles and MNI consider shaft thickness, section shape and medullar surface properties [71]. In this way, bones were divided into four anatomical regions: 1) cranial (antlers-horn, skull, mandible and dentition), 2) axial (vertebrae, ribs, pelvis and scapula, sensu [72]), 3) upper appendicular limbs (humerus, radius, ulna, femur, patella and tibia) and 4) lower appendicular limbs (metapodial, carpals, tarsals, phalanges and sesamoideal).

Mortality patterns were divided into (1) infants (individuals dead before the first six months of life, as shown by the absence of the first permanent molar), (2) juvenile-prime adults (individuals showing the second permanent molar and deciduous p4) and (3) adults (those with all permanent teeth). Age profiles were estimated from tooth crown wear and the emergence of the teeth according to Stelle [73] for deer, Pérez Ripoll [74] for ibex and Levine [75] for *Equus*.

A systematic observation of bone surfaces to explore the presence of cut, percussion and tooth marks was also carried out with 10X-20X hand lenses and different lighting [76]. Our diagnostic criteria for cut, tooth and percussion marks are the ones defined respectively by Bunn [77] and Potts and Shipman [78], Blumenschine [76], and Blumenschine and Selvaggio [79]. For comparative purposes, observation of bone surfaces includes the observation of epiphysis and shafts [76]. Modifications of bone surfaces were also quantified by types of fragments and bone sections [80, 81] based on NISP values. The presence of tooth, percussion and cut marks was recorded for the whole assemblages, and percentages of tooth, percussion and cut marks included only bones with a good surface preservation. Weathering stages were also observed following Behrensmeyer [82] to estimate the bone subaerial time exposure. Water effects on bone surfaces were estimated according to the presence of abrasion, polishing, rounded bones, and carbonates following Parson and Brett [83], Cáceres [84] and Yravedra [85]. Signs of polishing, rounding or abrasion are to be expected in transported assemblages, but also in non-transported assemblages exposed to circulating water and mobile sediments, such as those encased in sand strata [86]. Biochemical alterations were estimated according to Domínguez-Rodrigo & Barba [87]. To differentiate between green and dry fractures on long bones we analyzed shafts larger than 30 mm following Villa & Mahieu's [88] criteria.

### Lithic technology

**Fig 11:**
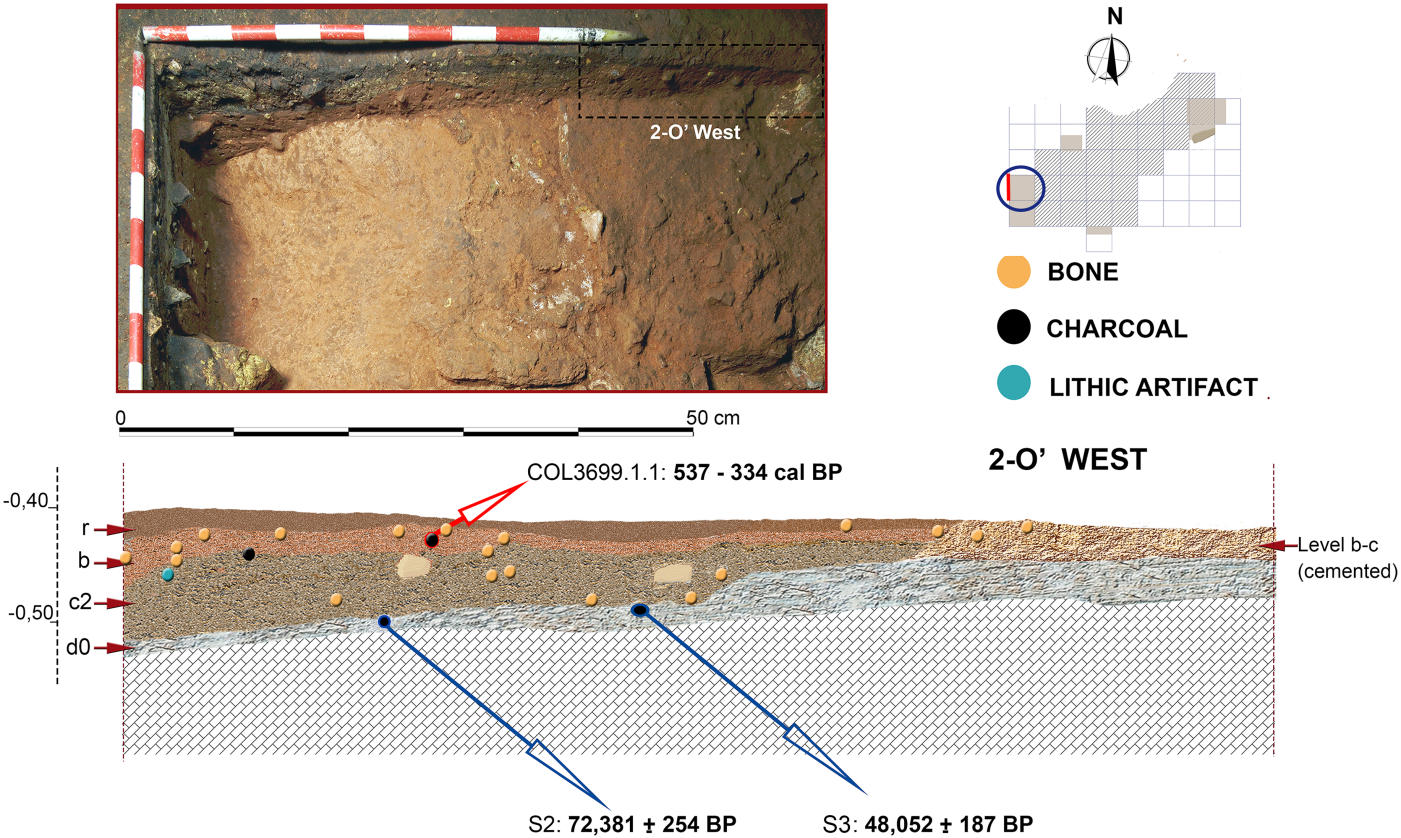
Stratigraphic sequence and vertical distribution of items recorded in profile 1-O’ South and 1-O’ West. Since some items could not be plotted in both views due to stratigraphic dip, topographic numbering of items has been included.

**Fig 12:**
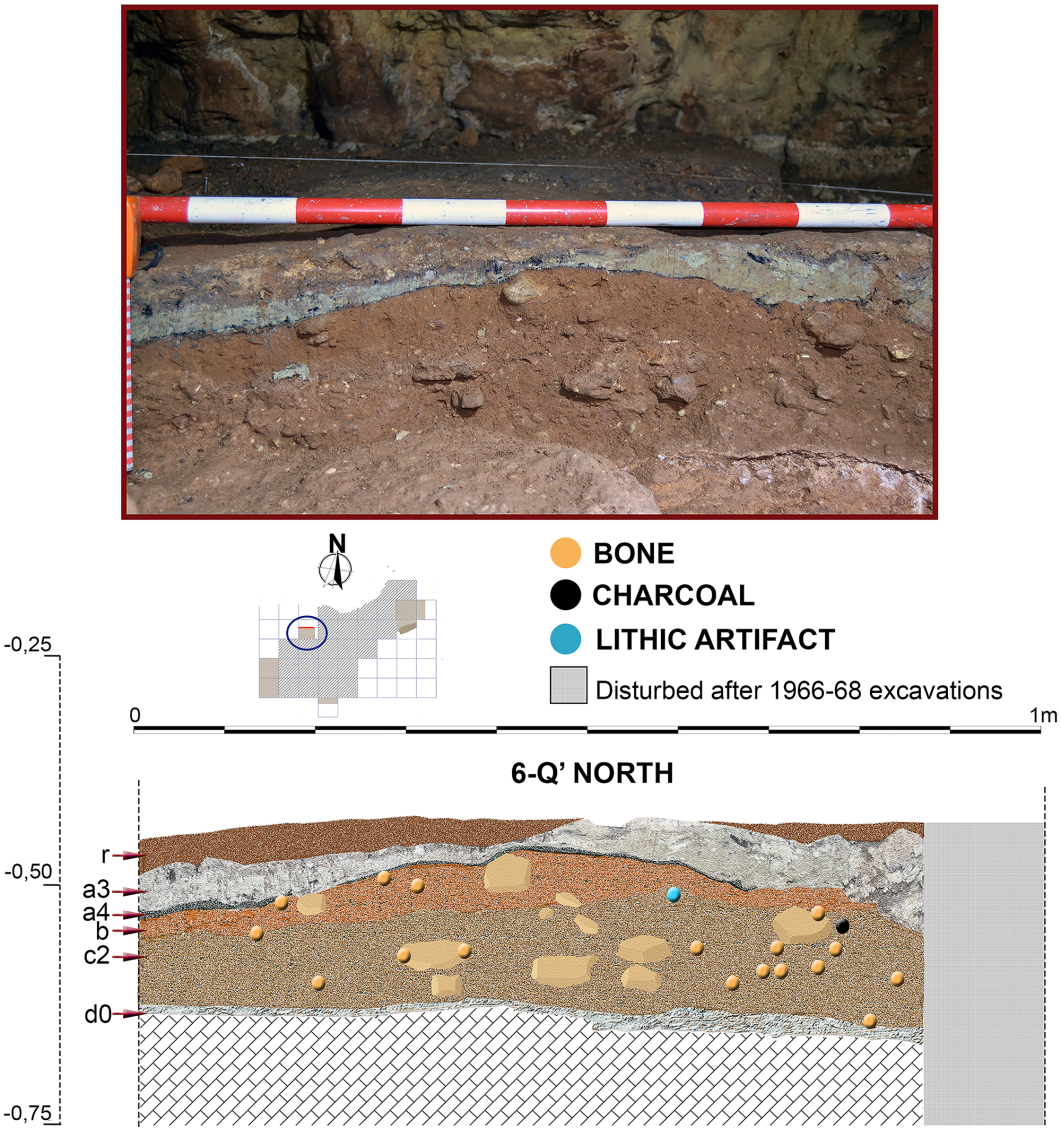
Stratigraphic sequence and vertical distribution of items recorded in profile 6-Q’ North.

**Fig 13:**
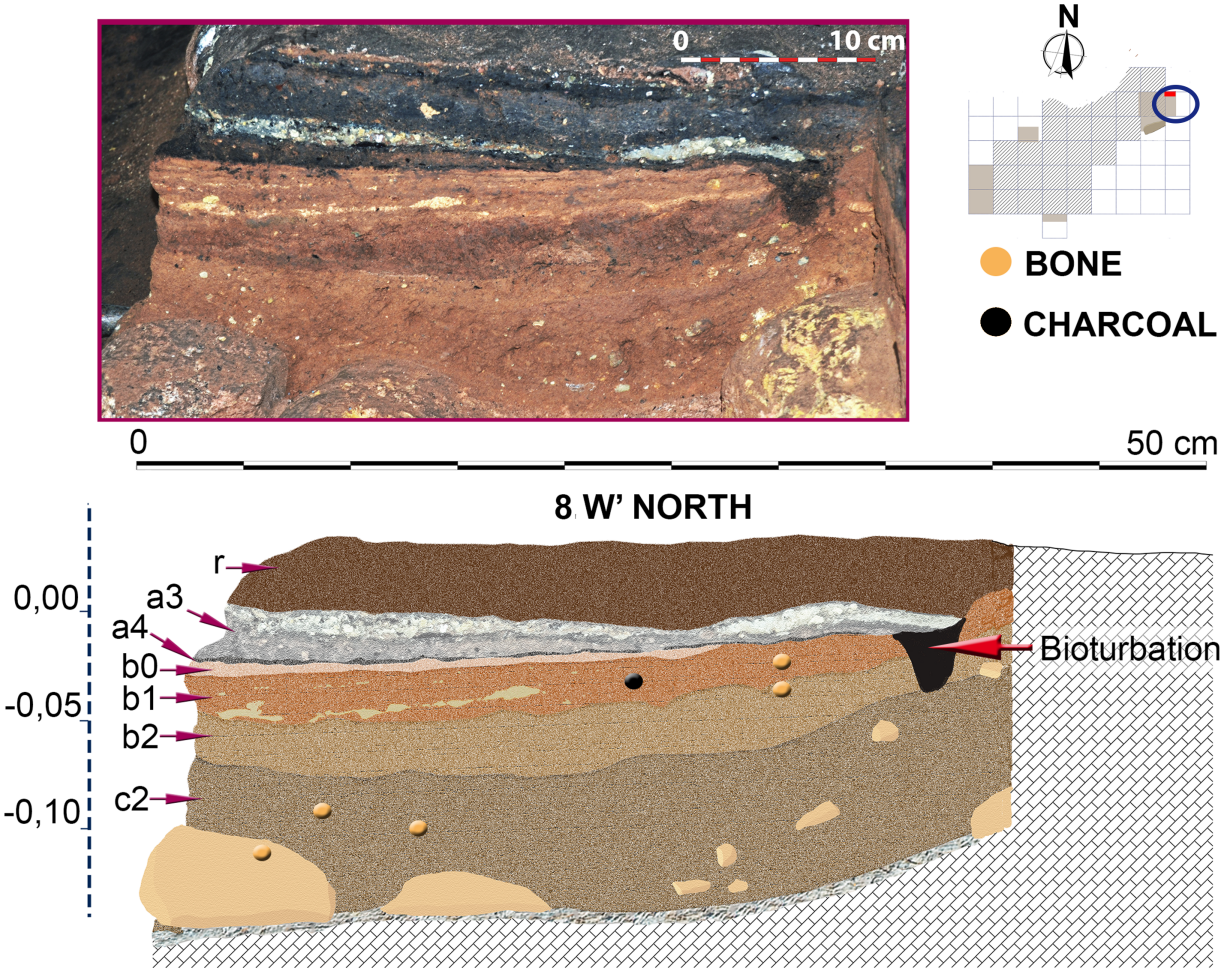
Stratigraphic sequence and vertical distribution of items recorded in profile 8-W’ North.

**Fig 14:**
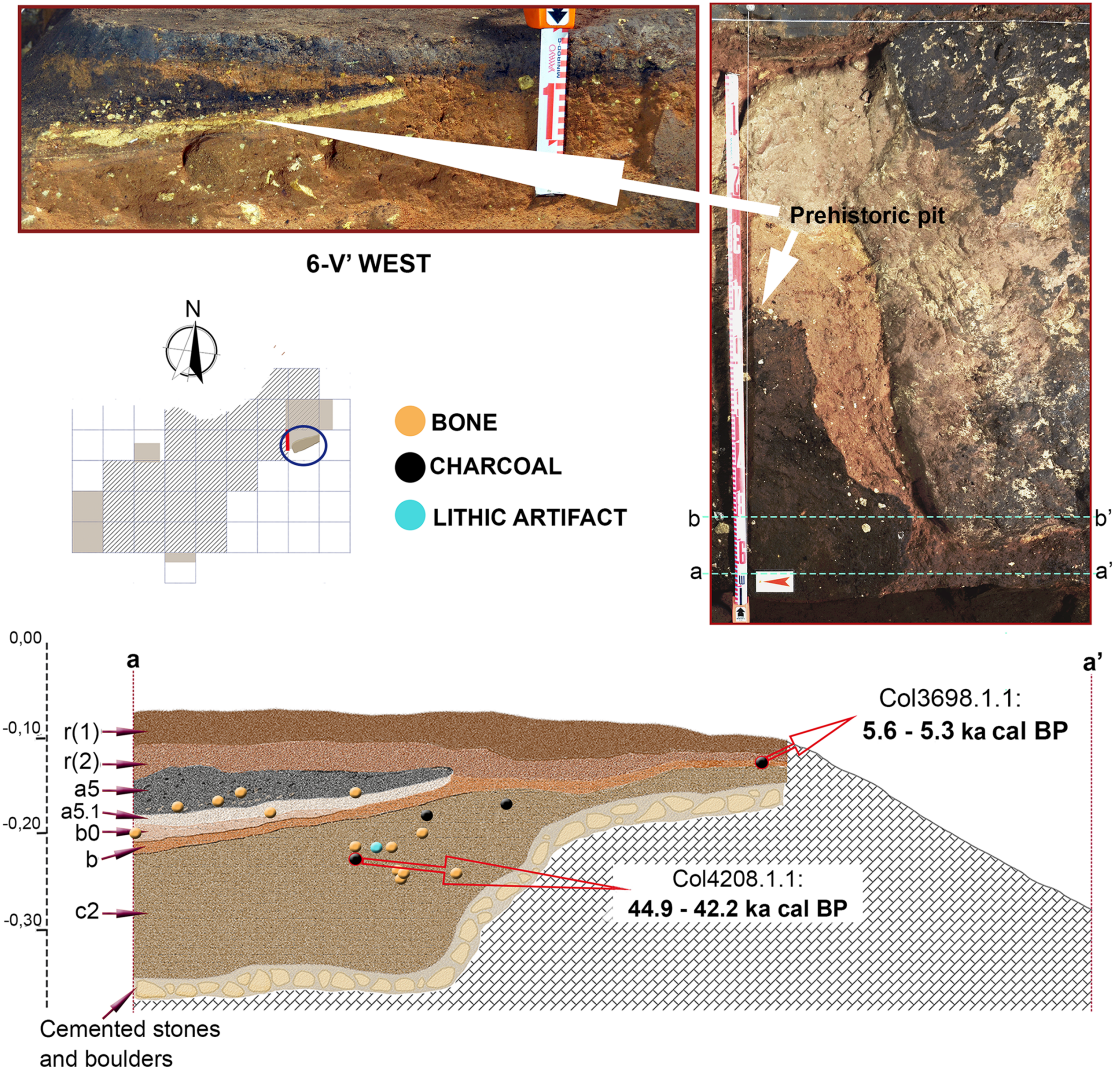
Stratigraphic sequence and vertical distribution of items recorded in profile 6-V’. Stratigraphic position of two dated charcoals is shown.

**Fig 15:**
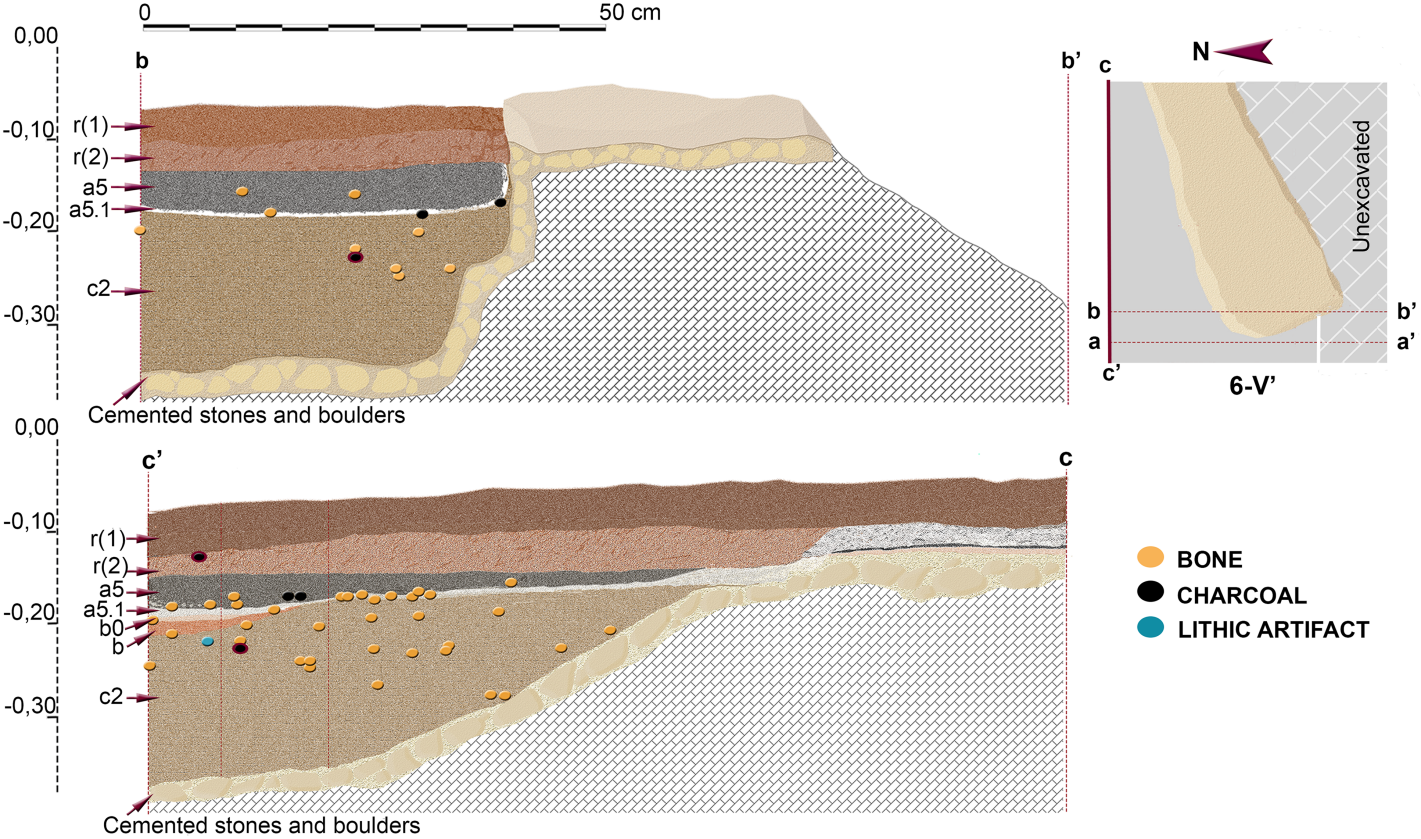
Additional views of stratigraphic sequence and vertical distribution of items recorded in profile 6-V’.

Besides one single flint flake collected in layer b, all lithic artefacts recovered at Los Casares-Seno A come from level c. In consonance with those recorded by Barandiarán [14] in the same level during the 1960’s excavations, they represent a scarce sample. In the *circa* 5 square meters excavated, we collected just 7 lithic artefacts, including two debris recovered after wet-sieving. Assemblages recovered at the Seno A during the 1960’, summing up to 38 lithic artefacts, were studied at the *Museo Arqueológico Nacional* (Madrid), where they are currently curated. However, we considered only 37 items, since one blade, found in mostly disturbed square 2-Q’ as reported by this scholar (Fig 6), must be conceived as a very likely intrusion from above [15]. Therefore, we analyzed a total of 44 lithic artefacts, 32 of them produced on flint (72.7%), and 12 on quartzite (27.3%). A spatial distribution of the whole assemblage was possible due to the spatial recording of objects during the old excavations [14]. Plotting of artefacts, both horizontal and vertical, is shown in Figs 6 and 9–15. Density of artefacts is very low, only reaching 1.76 artefacts per square meter.

Lithic artefacts were studied at the Prehistory Laboratory of the University of Alcalá under the chaîne opératoire or ‘operational sequence’ approach as described by Inizan et al. [89] and discussed in Bar-Yosef and Van Peer [90]. We assigned each lithic artefact to one of the three chaîne opératoire stages commonly recognized in the literature [90]. Thus, cortical flakes, preparation products and tested cores were assigned to the initialization stage or phase I, raw blanks, core maintenance by-products and rejuvenation flakes to the exploitation stage or phase II, and retouched blanks and exhausted cores to the consumption and abandonment stage or phase III. We also analyzed artefacts in terms of formal recycling and reuse processes as discussed by Amick [91] and Baena et al. [30].

## Results

### Stratigraphic sequence and micromorphology

Stratigraphic sequence documented in the Seno A deposit during 2014–2015, preliminarily published in [15], was first described after rejuvenation of profile 1-R’ South previously excavated by Barandiarán in the 1960’s. Main stratigraphic layers and sub-layers recognized at the deposit were first identified at this profile, corresponding to the northern sector of square 3-R’ (hereafter, just ‘profile 3-R’). Also, this is the only area where we excavated level d, while in other test pits we only reached the top of level d0. As previously mentioned, profile 3-R' showed a similar stratigraphy as documented in the 1960’s, but it was less affected by bioturbation, and disturbance reflected in a pit infilling at the western side of the profile was less severe than in the original profile 1-R' [15] (Fig 9). The top 2 to 3 cm of 3R', denominated as "r", consisted of compacted, dark brown and black sandy loam, containing weathered rock debris, pieces of charcoal and few artefacts. It represents a reworked surface layer probably compacted by trampling in the recent past. Layer "a2", defined by Barandiarán as a thin discontinuous black band with concentrations of charcoal, although partially visible in some areas, was integrated by us in layer r. Layer "a3" (about 5cm) consists of a light grey to greenish-yellow, densely packed silty clay loam, with inclusions of charcoal, weathered rock fragments and few Chalcolithic ceramics. At the base of this layer, another thin black band (a4) is found, very rich in charcoal and Chalcolithic pottery. Layers a3 and a4 are rich in fine silt and clay displaying a textural contrast to the underlying reddish brown sandy loams of layers b0 and b. The latter layer contains few gravel and shows some yellowish patches and several discontinuous black laminae, the lowermost one forming the lower boundary of layer b (about 4 cm thick). In contrast to the stratigraphy of Barandiarán (Fig 7A), we identified three sublevels for the sandy loams of layer c (up to 10 cm thick) as based on differences in colour and degree of compaction. Sublevel c3 was characterized by an orange colour and was only locally preserved. Below layer c, flowstone of level d0, up to 3 cm thick, was found. It covered heavily cemented reddish loams of level d, the latter being very rich in animal bone, but lacking any artefacts. Excavation at 3-R' stopped after reaching another stalagmitic crust correlating with layer e as defined by Barandiarán (Fig 7A).

Although presenting some minor differences concerning sub-layering of stratigraphic levels, sequence described in 3-R’ was also recognized in the other areas excavated in the Seno A (Figs 9–15). An important difference was however registered in square 6-V’, where a prehistoric pit, most probably Neolithic or Chalcolithic, and recorded as layers a5 and a5.1, penetrated the clays of layer b (Fig 14).

**Fig 16:**
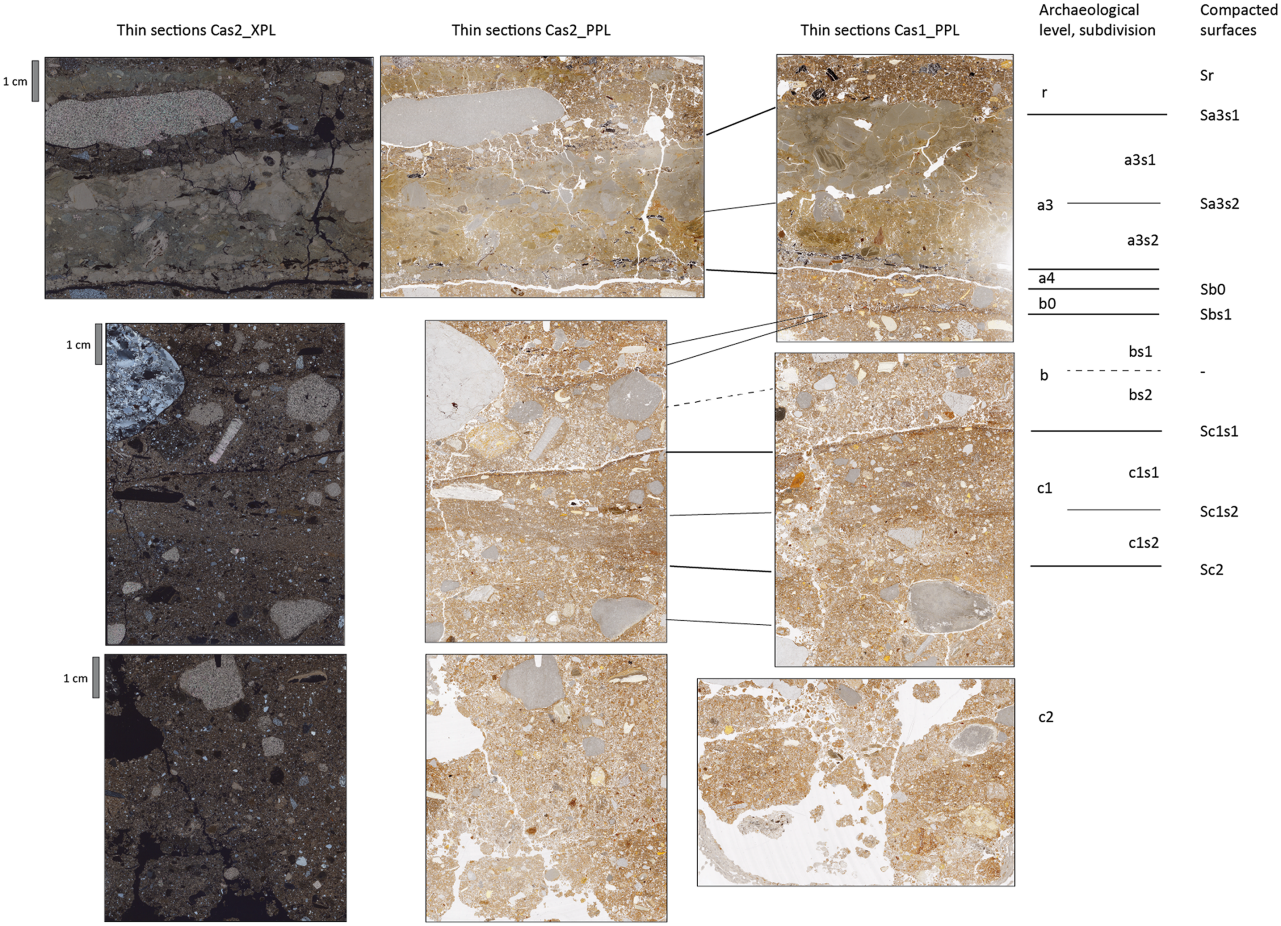
Thin section scans from profile 3R including microstratigraphic subdivision of archeological levels and presumed former surfaces of the cave floor, compacted by trampling. Thin sections on the left side were scanned using two polarization foils at 90° angle similar to crossed polarizers (XPL), while the other six were scanned without polarization foil (PPL).

**Fig 17:**
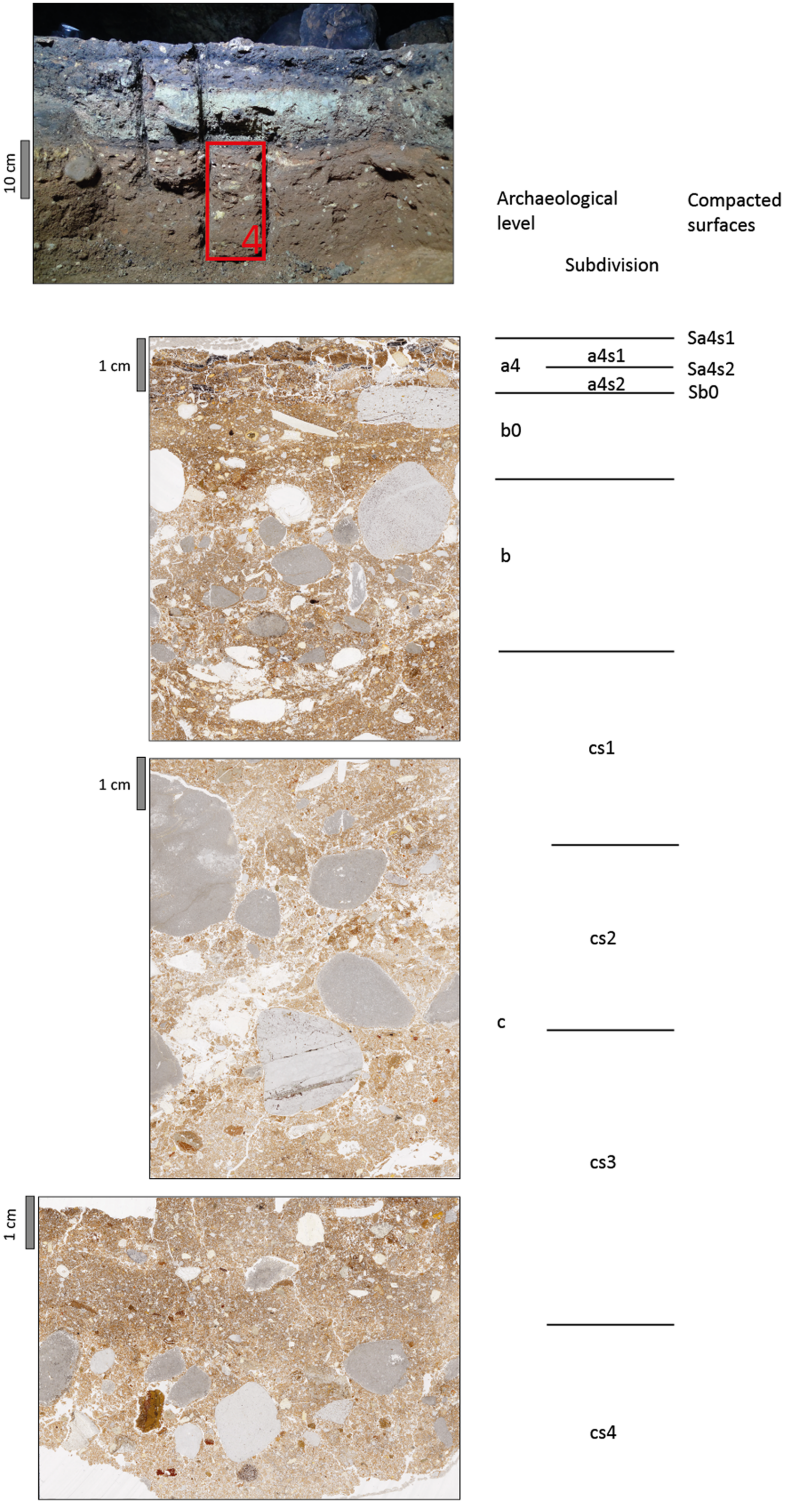
The profile of 1-O’ with location of sampling for micromorphology and scans of three thin sections including stratigraphy and compacted surfaces.

**Fig 18:**
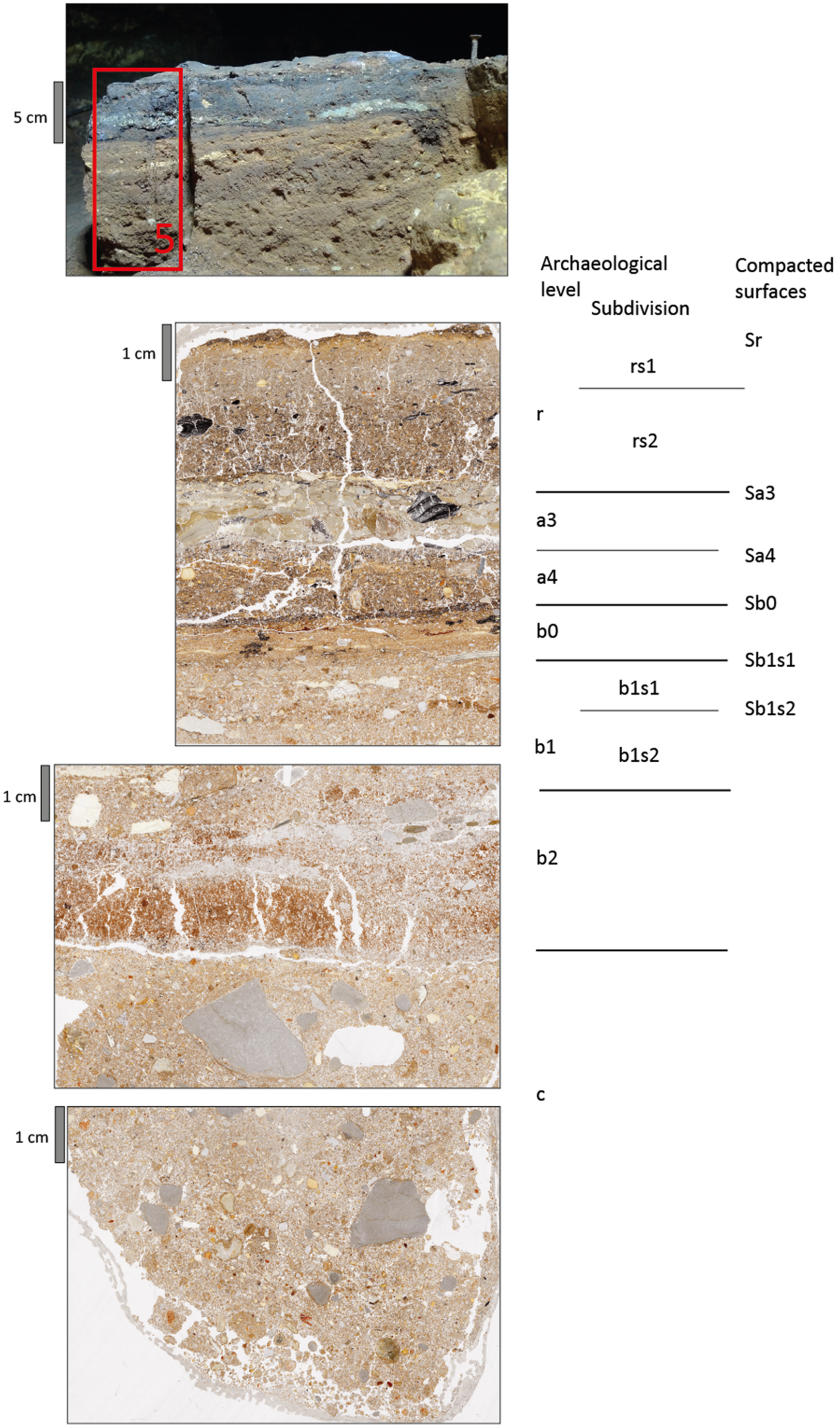
The profile of 8-W’ with location of sampling for micromorphology and scans of three thin sections including stratigraphy and compacted surfaces.

Detailed micromorphological descriptions of three different profiles are provided in tables A to C (S1 Appendix) and illustrations of the thin sections including microstratigraphic subdivision of archeological levels are shown in Figs 16–18. In some cases, archeological levels defined in the field included several sediment layers in thin section, which have been characterised separately, where appropriate. Monoliths 1, 2 and 5 cover most of the sequence including layers r to c, whereas monolith 4 covers the lower part of the sequence only, starting with a4. The profiles show slight differences in stratigraphy as indicated below.

Archeological level r consists of sandy loams rich in charcoal and bone fragments. The sediment layers detected in thin section are densely compacted. In profile 8-W’, level r can be subdivided based on color and composition of coarse materials. At the top, an orange microlayer is visible, which owes its color to phosphate accumulation probably from bat guano. Level a3 is rich in silt and clay and contains many partly disintegrating limestone fragments in its upper part, forming a light grey layer, which is found in all profiles sampled. This layer includes admixtures of charcoal and siliceous fines, best visible in thin section 5.1, and, in profile 3-R’, it can be subdivided into two sublayers, the lower of which containing considerably less rock fragments.

Level a3 overlies the dark coloured level a4. In profile 3-R’, a4 represents a black microlayer, few mm in thickness, while in the other two profiles sampled it is about 1 cm thick. Level a4 is mainly composed of fine pieces of charcoal and amorphous burned organic materials, concentrated in two different microlayers in profile 1-O’. In profile 8-W’, a4 is particularly rich in small phosphatic coprolites of bat guano.

**Fig 19:**
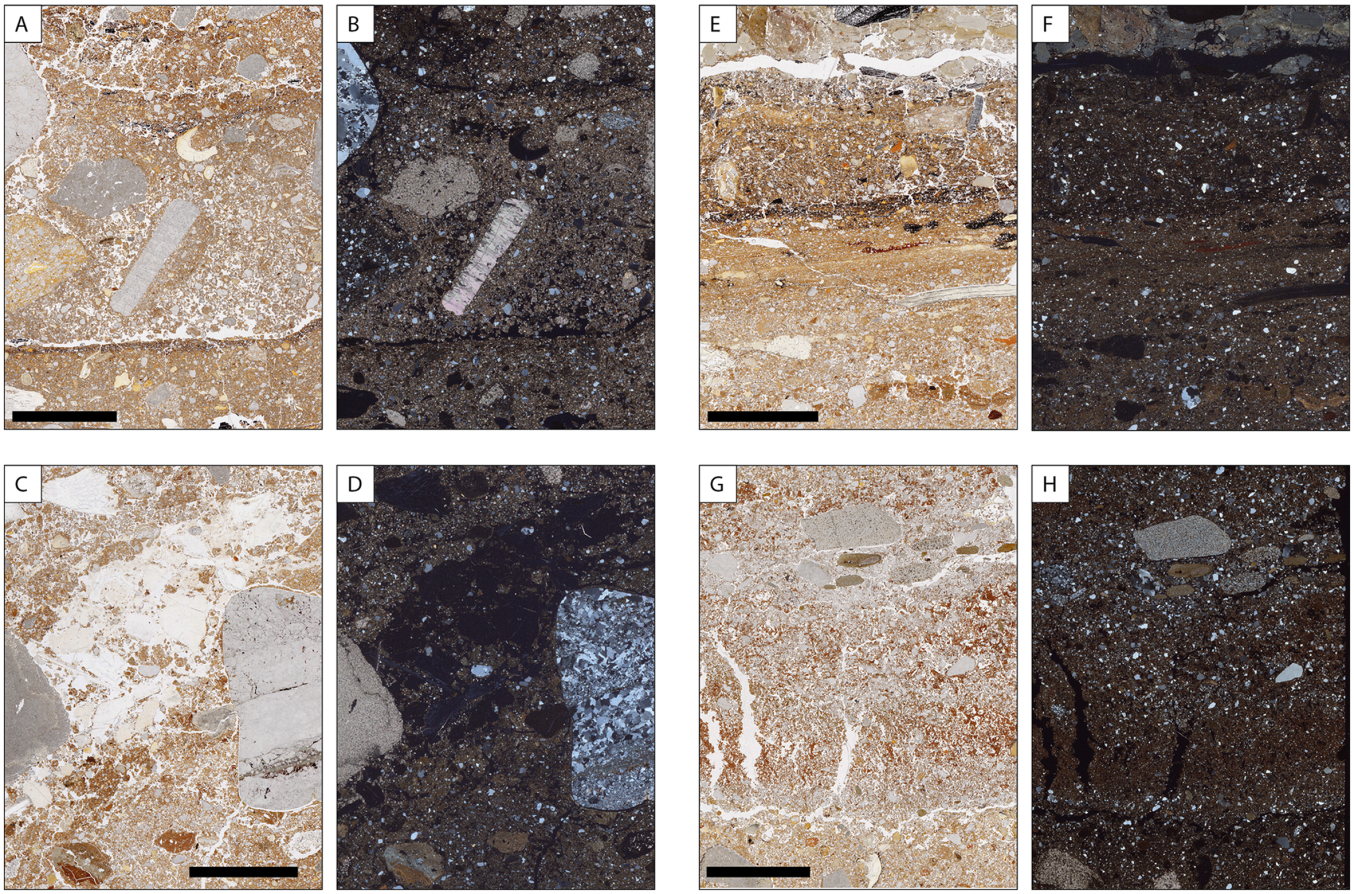
Selected areas of thin sections scanned under PPL and XPL conditions for illustration of some stratigraphic details on a larger scale. The scale bar represents 10 mm. A and B show the prominent dark colored interface between levels b and c1 in profile 3R. Note the low degree of compaction above the interface and the high packings density below it. In the upper part, linear accumulation of charcoal and manganese indicate former surfaces. C and D include a local accumulation of bone fragments with abundant phosphate infillings. Phosphate also precipitated in the outer part of the limestone gravel on the left. E and F include the sequence from level a3 at the top over a4, b0 to b at the bottom with sublayers of thin section 5/1. Note the strong compaction and linear lamination of the central layer (b0) and the remnants of textural surface crusts near the bottom. G and H is a close up of the boundary between b2 (top 3 cm) and c (bottom cm) in profile 8W. Under crossed polarization foils, the intercalation between clay rich and sandy layers with small gravel is visible.

The underlying sediments have a more reddish or orange brown groundmass. Level b0 is about 1 cm to 1.5 cm thick, shows a high degree of compaction and is moderately to strongly enriched in phosphate. In profiles 1-O’ and 8-W’, level b0 has a well-developed subhorizontally-laminated fabric (Fig 19E and 19F). Underlying layers of level b (or b1 in profile 8-W’) are much less compacted and less enriched in phosphate (e.g. Fig 19A and 19B). In profile 8-W’, this level was subdivided into two layers: b1 consists of sandy loam with bones and coprolites, while b2 represents an intercalation of several clayey or sandy to gravelly microlayers in subhorizontal orientation (Fig 19G and 19H). Layer b2 appears to be a locally preserved water-laid deposit almost free of coprolites, bones or charcoal.

Levels b1 and b2 both contrast in composition and fabric with the underlying sediments, belonging to level c. Sublevel c1 has been defined in the 3-R’ profile based on field evidence. It shows a high degree of compaction and can be further subdivided into two layers (c1s1/c1s2), where the lower part is rich in fines. Level c2 is similar to c1, but rich in coprolites and showing various pedofeatures as described below. Besides c1 in profile 3-R’ all other sediments of level c are quite similar in composition, while changes in gravel content and bone may occur, leading to distinction of separate layers.

The micromass of most sediments has a brown or reddish-brown color and dotted limpidity with black stains in particular in layers of levels a4 and r. Layers rich in carbonate showed crystallitic b-fabric, whereas stipple-speckled to mosaic-speckled and more rarely undifferentiated b-fabrics were present in other layers.

The pore space consists of many different types of voids, including vughs, complex packing voids, planes and burrows. Layers with high packing density show few vughs and a massive microstructure, while in those exhibiting lower packing density complex packing voids between granules and burrows or chambers can be found besides vughs, which, in combination, yield a granular or vughy microstructure.

The different amount of micromass is reflected by the presence of diverse coarse/fine related distribution patterns including close, single-spaced, double-spaced and open porphyric types.

Corrosion and mechanical disintegration of limestone fragments is relatively strong in layers of level c and in level a3. Carbonate leaching is indicated by local presence of undifferentiated b-fabrics, as well. Locally, heavily corroded carbonate grains are found in sand-size pores. Calcite pedofeatures including infillings and coatings indicate precipitation of secondary carbonates in several layers. Besides calcitic pedofeatures, few other types were detected. Locally, iron hydroxide or manganese oxide nodules, such as in level b0 of profile 8-W’ were found or animal burrows detected. In addition, phosphate infillings and phosphatic rims of limestone fragments (Fig 19C and 19D) were found.

A prominent feature in the studied thin sections is the presence of sharp boundaries between levels or layers delineated by enrichment with small charcoal fragments (e.g., level b to c1 in profile 3-R’) or manganese precipitates or by remnants of small sedimentary crusts of fine materials (e.g., between b1s1 and b1s2 of profile 8-W’) (Figs 18, 19E and 19F). In several cases, the degree of compaction is high directly underneath these sediment interfaces, and packing density decreases with depth before another boundary is reached (Fig 19A and 19B). For instance, this is visible across the boundary between levels b0 and b in profile 3-R’. The various degrees of compaction within one layer and the generally high degree of compaction in levels r, a3 and b0 most probably relate to trampling. This kind of compacted surfaces with sharp upper boundaries is found down to level c1 (Figs 16–18).

### Uranium /Thorium dates

U/Th dates obtained for Los Casares-Seno A flowstone samples are shown in Table 1. Since all samples were collected all along the same speleothem formation in different areas of the Seno A deposit (Figs 9 and 11), internal layering of samples must be considered for chronological control. The most recent dates were obtained for the upper layers of S1 (sample S1b) and S3, corresponding to Marine Isotopic Stage (MIS) 3. This time frame (*c*. 53–48 ka BP) can be taken as the period of last speleothem formation in the cave. The older date was obtained in S1a, thus proposing a minimum age for the beginning of speleothem growth at early MIS 5. Between these dates (*c*. 124–48 ka BP), accumulation of the oncolites layer present in S1 was produced, most probably after *c*. 72 ka BP if we consider the date obtained in S2. In sum, these data suggest that speleothem formation at Los Casares-Seno A occurred mainly during the wet and warm interglacial periods of MIS 5 and MIS 3. A hiatus in this process occurred during most of MIS 4, when accumulation of oncolites is registered at some parts of the deposit. Similar processes of long and discontinuous speleothem growth have been recently recorded at other caves in the Iberian Peninsula [92].

**Table 1:** Uranium/Thorium dates obtained on speleothem samples collected at Los Casares cave—Seno A.

Sample	^238^U	^232^Th	^230^Th/^232^Th	δ^234^U	^230^Th/^238^U	^230^Th age	^230^Th age corrected
	ng g^-1^	ng g^-1^	Atomic ratio (x10^-6^)		Activity Ratio	years BP	years BP
**S1a**	53 728 ± 537	150.82 ± 3.02	4291 ± 300	92.5 ± 2.7	0.756 ± 0.004	125 151 ± 500	124 667 ± 498
**S2**	4 214 ± 42	10.698 ± 0.214	3502 ± 280	133.5 ± 2.8	0.558 ± 0.003	72 509 ± 254	72 381 ± 254
**S3**	4 127 ± 41	2.957 ± 0.059	8830 ± 1766	102.6 ± 2.8	0.397 ± 0.002	48 083 ± 187	48 052 ± 187
**S1b**	6 131 ± 61	10.596 ± 0.208	3921 ± 196	74.2 ± 2.2	0.422 ± 0.008	53 768 ± 785	53 511 ± 780

Since obtained ages are in stratigraphic agreement within the speleothem deposit (layer d0), the most recent date, corresponding to sample S3, can be taken as a *terminus post quem* for the Mousterian assemblages of layer c.

### Radiocarbon dates

Table 2 compiles radiocarbon ages for Los Casares-Seno A sequence. Since all attempted bones failed, only charcoal dates are available. The two samples collected in layer b yielded Holocene ages, while that collected in layer c falls within MIS 3. Although no other chronological markers are available for layer b, where few bones were recorded and just one lithic flake was found, both dated charcoal for this level are best explained as the result of intrusions coming from the upper part of the sequence. COL3699.1.1 (537–334 cal BP or 1,413–1,617 AD) was collected in square 2-O, where layer b was found immediately underneath the surface layer (r) and part of the sequence was cemented and probably disturbed. Exact position of dated sample was in fact just 3 cm below the surface layer (Fig 11). Although no micromorphological sample was taken at this square, it seems quite evident that the dated charcoal reflects a modern incursion into the cave. This hypothesis is consistent with the presence of both archeological materials on the surface, and *graffiti* on the walls, ranging from the Middle Ages to the 20^th^ century [14–16].

**Table 2:** Radiocarbon dates obtained on charcoal samples collected at of Los Casares cave-Seno A deposit.

Layer	Sample	Lab-ID	F^14^C	C^14^ BP	δ13C (‰)	C (μg)	Age cal BC/AD (95,4%)	Age cal BP (95,4%)
**b**	Charcoal (*Pinus nigra*)	COL3698.1.1	0.560 ± 0.003	4,653 ± 44	-25.5	999	3,626–3,353 BC	5,575–5,302
**b**	Charcoal (*Pinus nigra*)	COL3699.1.1	0.947 ± 0.004	439 ± 36	-24.4	997	1,413–1,617 AD	537–334
**c**	Charcoal (*Coniferae*)	COL4208.1.1	0.007 ± 0.001	39,494 ± 850	-27.7	899	42,950–40,226 BC	44,899–42,175

As for COL3698.1.1 (5.6–5.3 ka cal BP), its location in square 6-V is more complex. This square was affected by a prehistoric pit, probably produced during Chalcolithic or Neolithic times, penetrating the northern area of the sequence up to layer b0 (Fig 14). Charcoal was not collected in the disturbed area as identified during fieldwork, but at the base of level b, 26 cm southward of the pit’s edge. However, considering its proximity to the disturbed area, the most parsimonious interpretation of the age obtained is that it reflects a Neolithic intrusion of charcoal into the clays of layer b, thus suggesting that this layer was also disturbed at the southern area of the square. A counterhypothesis is that layer b is in fact Neolithic, but this is very unlikely given its sedimentological and geochemical composition, very similar to that of layer c. Further evidence based on micromammal assemblages found in this layer also points to a Late Pleistocene age, as it will be discussed below.

**Fig 20:**
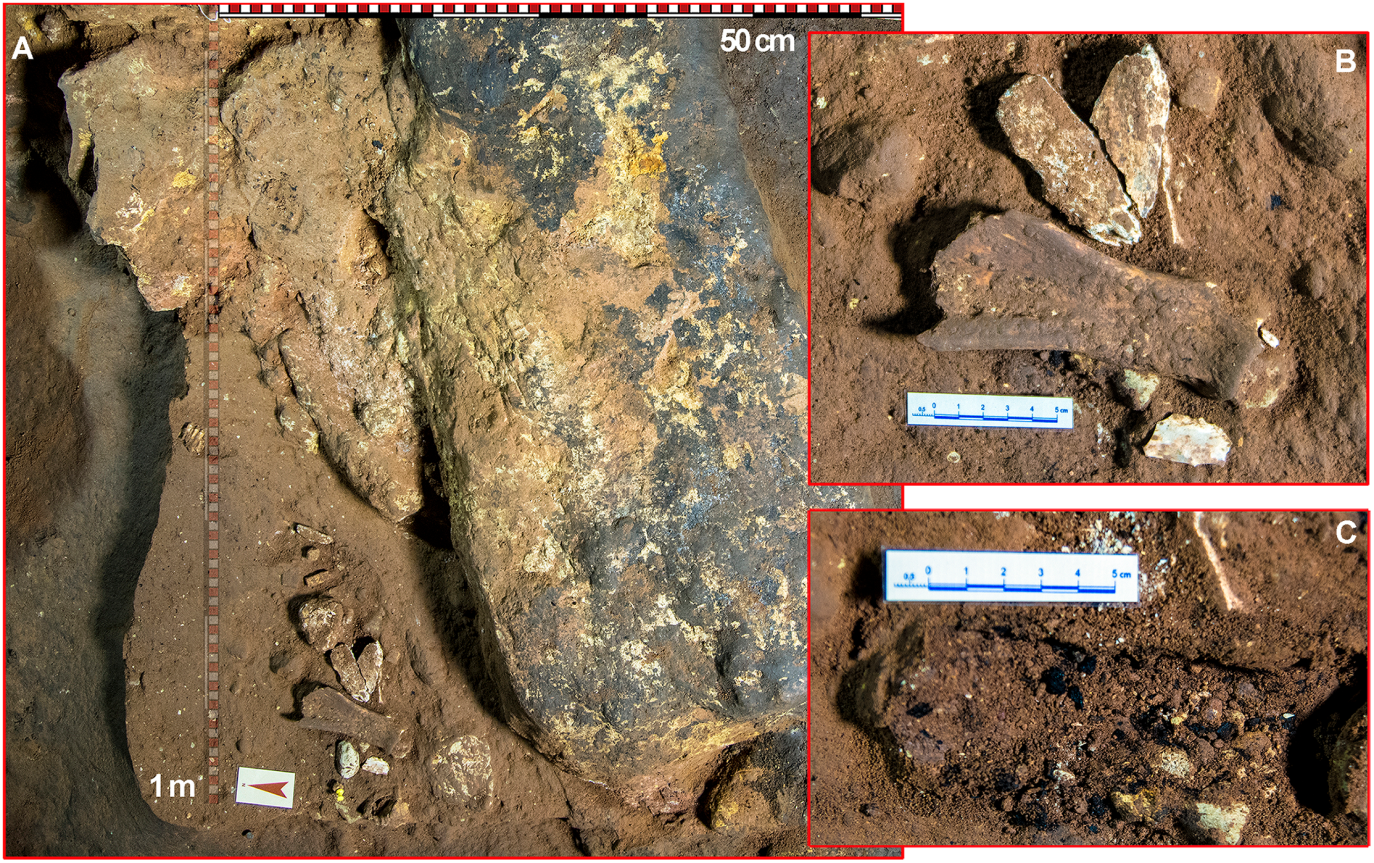
Location of charcoal sample COL4208.1.1. in square 6-V’. A: General view of the excavation of level c2 in square 6V’. B: Detailed view of a deer scapula and associated faunal and lithic remains. C: Charcoal remains collected for radiocarbon dating below the deer scapula.

COL4208.1.1 (44.9–42.2 ka cal BP) is the only sample collected in layer c and therefore the only potentially related to the Mousterian occupation of Los Casares-Seno A. Besides this sample was also taken at square 6-V and no micromorphological analysis was conducted here, the stratigraphic position and archeological context of this sample ensure its reliability as a chronological marker of the Mousterian occupation for three main reasons. First, it was found more than 5 cm below the pit, whose lower limits were accurately identified. Second, compacted surfaces, as defined by micromorphological evidence in other profiles, were found above the dated sample separating layers b0, b and c2, thus suggesting that this part of the sequence was preserved *in situ*. And third, the charcoal fragment was not only collected in an area with a high density of items including a flake (i.e. associated to human activity), but also it was completely covered by a deer scapula found in horizontal position, thus making very difficult its putative contamination or intrusion from above. In Fig 20 we illustrate the exact location of this charcoal sample. Unfortunately, dating of the scapula covering the charcoal was unsuccessful due to no collagen yield.

### Pollen

**Fig 21:**
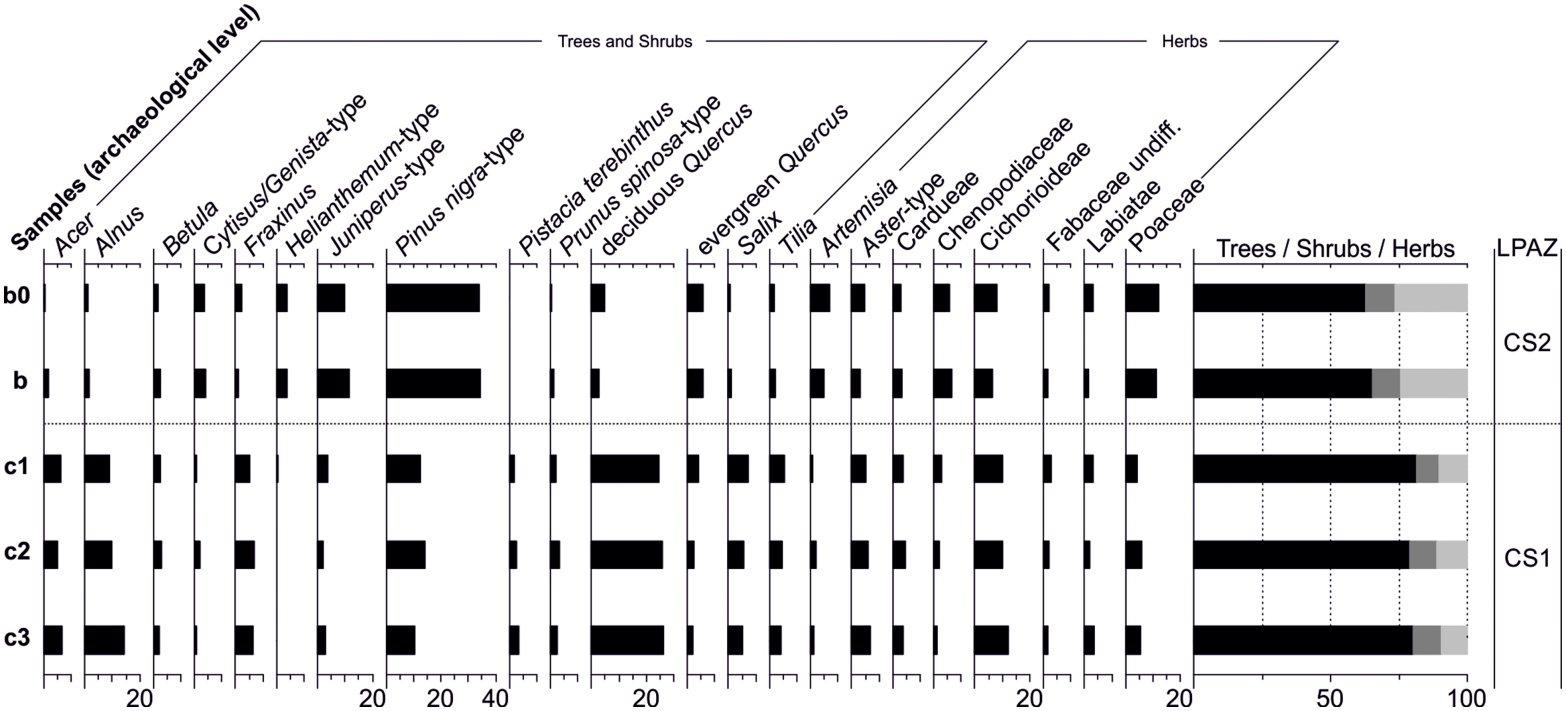
Percentage pollen diagram from Los Casares cave-Seno A site.

**Fig 22:**
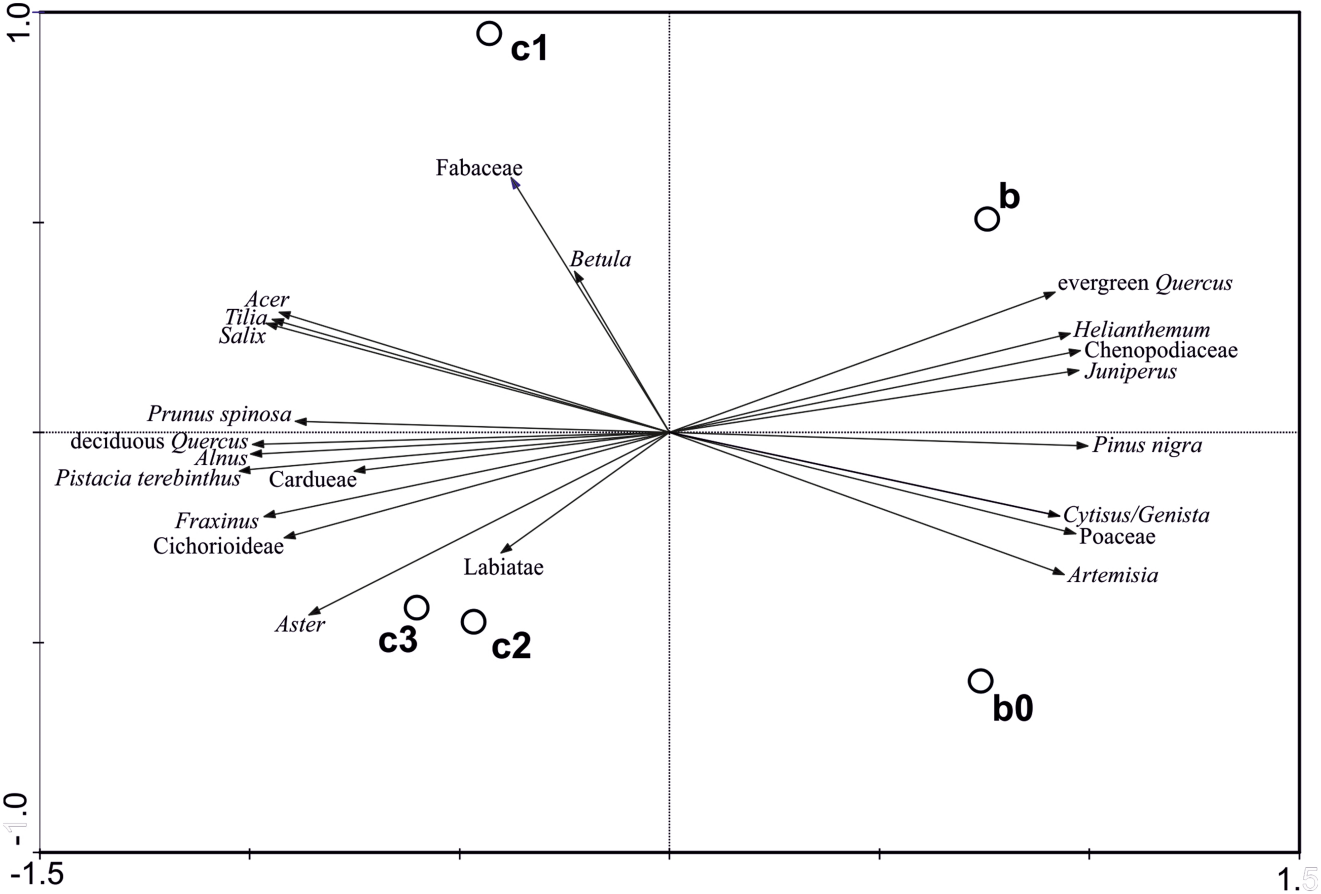
PCA biplot from Los Casares cave-Seno A site showing ordination of samples and pollen taxa.

For Los Casares-Seno A sequence, 5 pollen spectra were analyzed and 22 taxa were identified. To facilitate description and interpretation of the pollen diagram with respect to vegetational changes, two Local Pollen Assemblage Zones (LPAZs) were established (Fig 21). These zones denote significant changes in the pollen composition and represent major changes in vegetation. LPAZ CS1 is dominated by deciduous *Quercus* (24.8–26.2%), *Pinus nigra* (10.3–12.6%), *Alnus* (8.9–14.3%) and Cichorioideae (10.2–12.3%), as well as by other mesophilous trees (*Tilia*, *Fraxinus*, *Acer*). As for LPAZ CS2, it is dominated by *Pinus nigra* (34.1–34.6%), *Juniperus* (9.8–11.5%), Poaceae (11.3–12%), *Artemisia* (5.1–6.9%), Chenopodiaceae (5.7–6.7%) and evergreen *Quercus* (5.7–5.9%). A PCA biplot of the sample scores of individual spectra and loading (eigenvectors) for the pollen types of Los Casares-Seno A record is shown in Fig 22. The PCA biplot shows a clear separation between samples of both LPAZs. The first two axes explain 96% of the variance in the dataset. Evergreen *Quercus*, *Helianthemum*, Chenopodiaceae, *Juniperus*, *Pinus nigra*, *Cytisus*/*Genista*, Poaceae and *Artemisia* have high positive values on PCA axis 1, which explains 93% of the variance in the dataset, while deciduous *Quercus*, *Acer*, *Tilia*, *Salix*, *Prunus spinosa*, *Alnus*, *Pistacia terebinthus* and *Fraxinus* are found on the negative side of PCA-1. These data suggest that the first component discriminates between mesophilous forests (negative values; LPAZ CS1) and pollen spectra representing black pine woodlands and shrublands (*Juniperus*, *Cytisus*/*Genista*) (positive values; LPAZ CS2). The second axis (PCA-2: 0.3%) does not show a clear discrimination between pollen taxa.

### Microvertebrates

**Fig 23:**
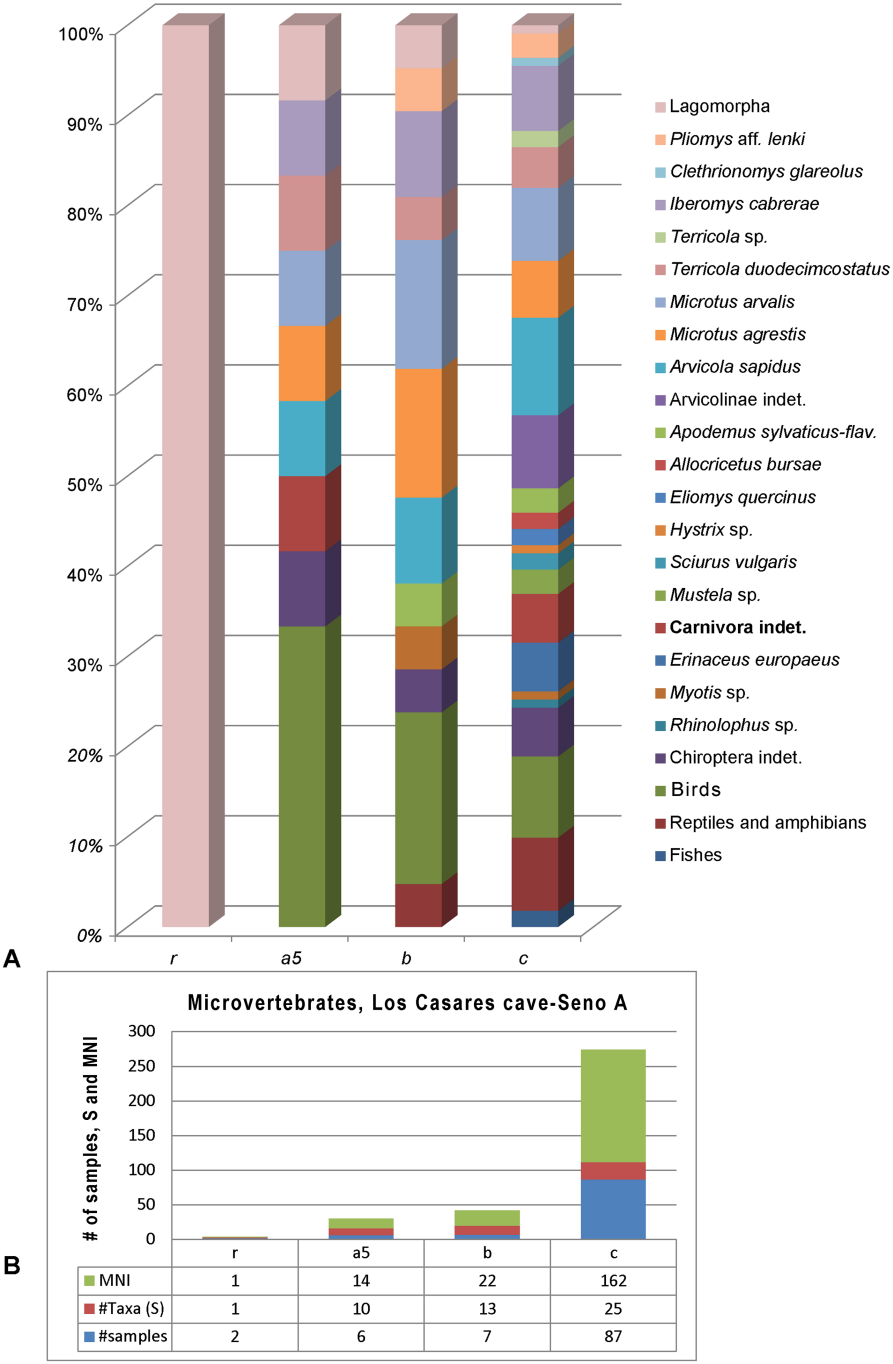
Distribution of small vertebrates identified in Los Casares cave-Seno A. A: Percentage of identified taxa per stratigraphic layer. B: Distribution (#) of analyzed samples, identified taxa (S) and Minimum Number of individuals (MNI).

After analysis of the 102 samples containing microfaunal remains, assemblages identified at Los Casares-Seno A are mainly composed of small mammals, although some bone fragments of fish, amphibian, reptile, and bird are present, albeit scarce and bad preserved. The small mammals are species of the orders Chiroptera (bats), Eulipotyphla (insectivores), Lagomorpha (rabbits) and Rodentia (mice), although the three first Orders are very poorly represented. Note that some medium-sized rodents are described in the large mammal section (see 4.8.). Table 3 and Fig 23 show the stratigraphic distribution of remains and MNI corresponding to these species in Los Casares-Seno A. Specimens are grouped in 25 taxa (S), the majority of which were determined to the species level.

**Table 3:** Microfaunal remains identified in Los Casares cave-Seno A.

Level	r	a5	b	c
**Fishes**	0	0	0	2
**Reptiles and amphibians**	0	0	1	9
**Birds**	0	4	4	10
**Chiroptera indet.**	0	1	1	6
***Rhinolophus* sp.**	0	0	0	1
***Myotis* sp.**	0	0	1	1
***Erinaceus europaeus***	0	0	0	6
**Carnivora indet.**	0	1	0	6
***Mustela* sp.**	0	0	0	3
**Rodentia indet.**	0	2	1	9
***Sciurus vulgaris***	0	0	0	2
***Hystrix* sp.**	0	0	0	1
***Eliomys quercinus***	0	0	0	2
***Allocricetus bursae***	0	0	0	2
***Apodemus sylvaticus-flavicollis***	0	0	1	3
**Arvicolinae indet.**	0	0	0	9
***Arvicola sapidus***	0	1	2	12
***Microtus agrestis***	0	1	3	7
***Microtus arvalis***	0	1	3	9
***Terricola duodecimcostatus***	0	1	1	5
***Terricola* sp.**	0	0	0	2
***Iberomys cabrerae***	0	1	2	8
***Clethrionomys glareolus***	0	0	0	1
***Pliomys aff*. *lenki***	0	0	1	3
**Lagomorpha**	1	4	5	63
**#samples**	2	6	7	87
**#Taxa (S)**	1	10	13	25
**MNI**	1	14	22	162

“#samples” refers to the number of plastic bags collected for the small vertebrate study.

**Fig 24:**
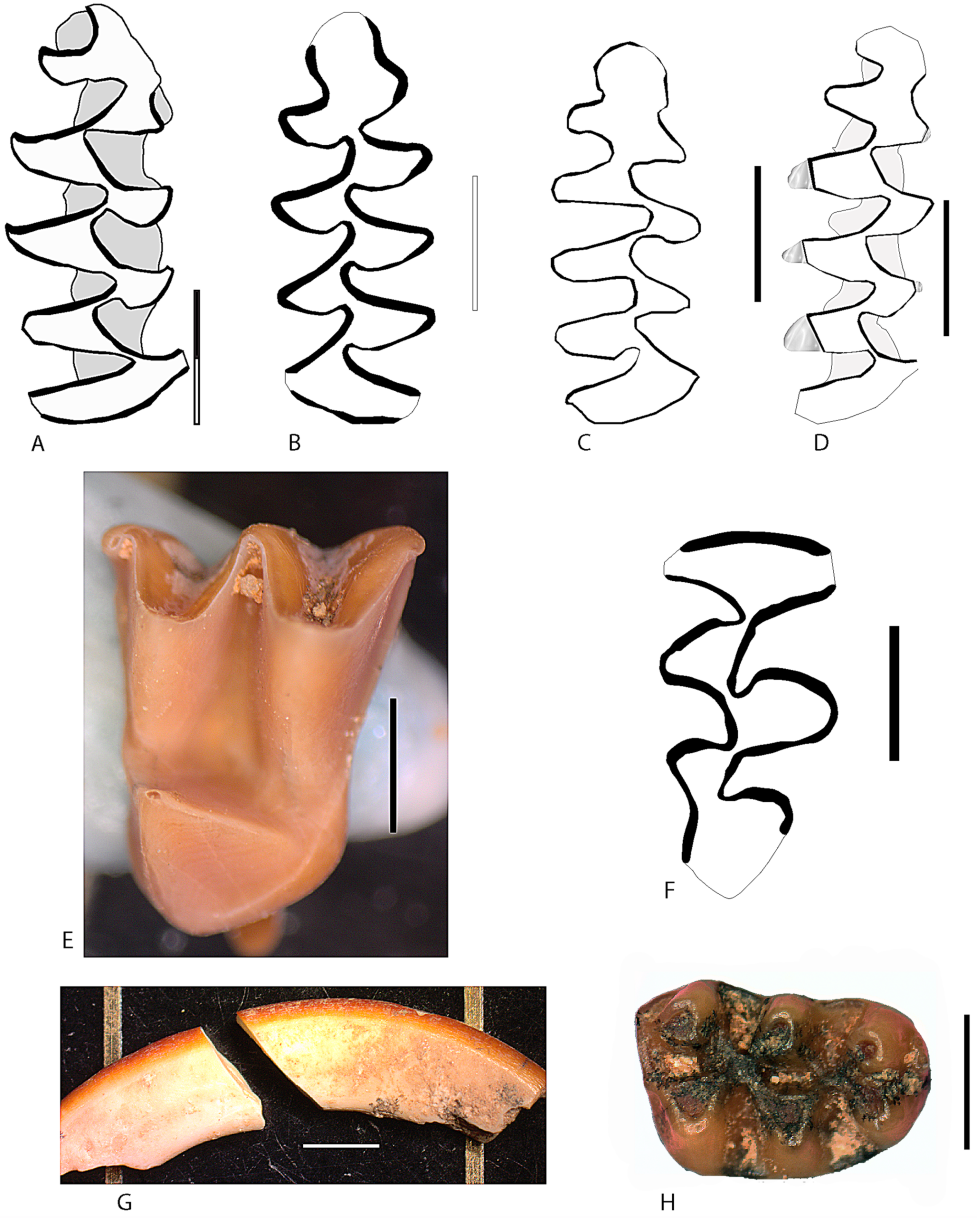
Selected specimens from level c of Los Casares cave-Seno A. A: m1d of *Iberomys cabrerae*. B: m1s of *Pliomys lenki*. C: m1d of *Microtus arvalis* juvenile (cement not drawn). D: m1d of *Microtus agrestis* (the lingual points of the salient angles are digested) E: M1s of *Myotis* sp. F: M3s of *Arvicola sapidus*. G: lower incisor of *Sciurus vulgaris*. H: M1d of *Allocricetus bursae*. The scale bar in each figure represents 1mm, except for figure G, where it is 2mm.

The species of bats is *Myotis Myotis* gr. *myotis/blythii* and it is represented by only one molar (Fig 24) and one canine in the layer b. Insectivores are also represented by a single species, the hedgehog *Erinaceus uropaeus*, a molar of which was found in layer c. Rodents are the most represent taxa, both in species and in MNI. There are 12 species, three of which are extinct: *Pliomys lenki*, *Allocricetus bursae*, and *Hystrix* sp. The rest, including *Eliomys quercinus*, *Apodemus* sp., *Arvicola sapidus*, *Microtus agrestis*, *M*. *arvalis*, *Terricola duodecimcostatus*, *Terricola* sp. and *Iberomys cabrerae*, are living today in the Iberian Peninsula (Figs 23 and 24).

The upper molar (M1d) of *Allocricetus bursae* has the same size (length = 2,14 mm, width = 1,48 mm) as recorded in other Iberian Middle and Late Pleistocene sites [93]. Note that some authors classify *A*. *bursae* as *Cricetulus*. We consider that the species *C*. *migratorius*, the extant Asiatic hamster, is smaller and exhibits more simple traits on the occlusal surface of the molars [77]. The same applies to m1 specimens of *Pliomys lenki* (length = 3,3 mm, width = 1,3 mm), which are similar in morphology and size to those recovered in the Late Pleistocene sequence of El Mirón cave (Cantabria) [94].

### Wood charcoal

Charcoal preserved at the site is scarce and remains were scattered throughout the excavated area in levels b and c. Only a relevant concentration of charcoal fragments, collected both by hand and flotation, was recorded at square 6-V’. However, most of these charcoals come from layer a5, defined as a Holocene intrusion, and hence they have no relevance for studying Middle Paleolithic environments (Fig 14). In general terms, since amount of charcoal remains recovered at this site is very low, results, compiled in Table 4, should be considered carefully.

**Table 4:** Wood charcoal identified in Los Casares cave-Seno A.

Layer	c	b	a5
Unidentifiable angiosperm			1
Coniferae	6		3
*Fraxinus sp*.			1
Leguminosae			1
*Pinus* t. sylvestris-nigra	7	7	14
*Quercus* sp. deciduous			3
*Quercus* sp. evergreen			1
**TOTAL**	13	7	24

Samples of levels c and b show a very low taxonomic diversity, since Scots pine type (*Pinus* t. *sylvestris-nigra*) was the only identified species. In level c some remains could only be assigned to Coniferae due to the small size of the remains. Concerning level a5, in addition to pine, several angiosperms have been identified: ash (*Fraxinus* sp.), oak (*Quercus* sp. Deciduous), holm-kermes oak (*Quercus* sp. Evergreen), Leguminosae and an unidentifiable angiosperm.

### Phytoliths

A total of 384 phytoliths corresponding to 20 different morphotypes were identified in the samples, of which 167 phytoliths corresponded to c1, 210 to c2 and only 7 to c3. Due to the low amounts of phytoliths found in the latter, this sample was not included in the study. Weathering of phytoliths represented around 20% of the total phytolith assemblage (Table 5).

**Table 5:** Main phytolith results.

Sample ID	AIF %	# of Phytolith morphologically identified	Dissolution %
**c1**	82.35	167	21.56
**c2**	73.72	210	18.57
**c3**	80.80	7	

% of Acid Insoluble Fraction (AIF), number of phytoliths morphologically identified and percentage dissolution of phytoliths.

**Fig 25:**
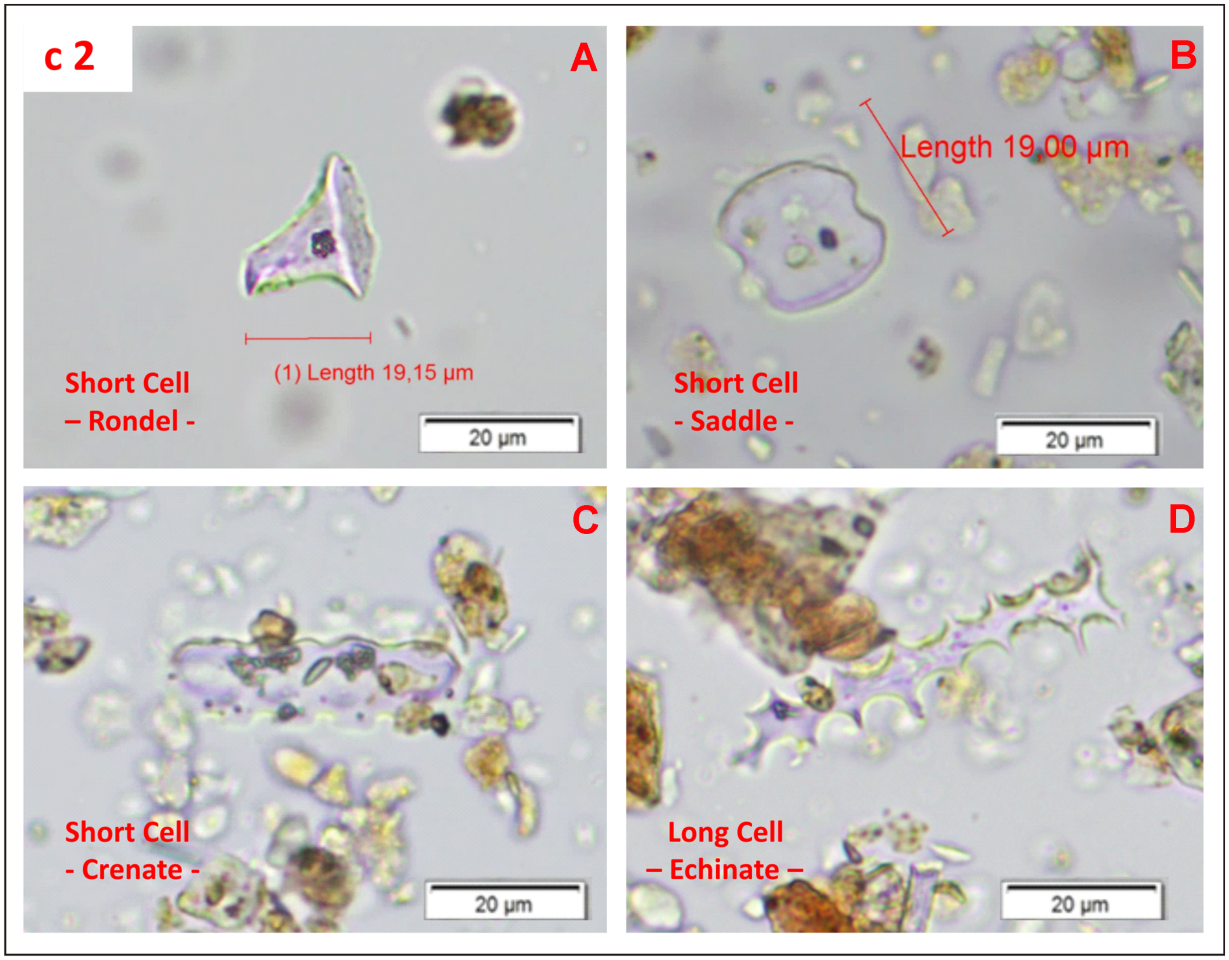
Microphotographs of phytoliths identified at level c Los Casares cave-Seno A. Pictures were taken at 400x. A) Rondel short cell; characteristic of *Pooideae* grass subfamily; B) Saddle short cell characteristic of the *Chlorodoideae* grass subfamily; C) Crenate; phytolith characteristic of *Pooideae* grass subfamily (*Gramineae*); D) Elongate echinate from inflorescence of *Poaceae* (*Gramineae*).

Samples c1 and c2 showed an abundant presence of phytoliths from the Poaceae (grass) family with 53.3% in c1 and 43% in c2. Within this family, short cells of the rondel type represented 19,8% in c1 and 11.9% in c2 of the total phytolith counting (Fig 25A) and (Table 6). Short cell rondels are common in the C_3_ photosynthetic Pooideae grass subfamily, which is common in the Mediterranean area and characteristic of a temperate and humid climate (Twiss *et al*., 1992, Piperno 2006). Short cell saddles (Fig 25B) characteristic of the C_4_ Chloridoid grass subfamily and common in drier and warmer environments were also noticed but in lower amounts (around 2.4%). Other grass morphotypes representing different plants parts such as leaves (bulliforms, crenates, etc.) (Fig 25C) as well as the inflorescence (elongates echinate, dendritic, papillae, etc.) (Fig 25D) were also recognized in the samples. Characteristic sedge (Cyperaceae) phytoliths were noted and represented between 1.2 and 2.4% of the total phytolith counting [59, 60, 95].

**Table 6:** Phytolith morphotypes identified in level c of Los Casares cave—Seno A.

Phytolith Morphotypes	c1%	c2%	Plant attribution
Blocky	3.0	10.0	Dicotyledon / Pinophyta
Tracheid	0.0	1.4	
Spheroid	1.8	1.0	Dicotyledon
Hair	11.4	8.1	
Elongate Psilate	21.6	25.2	Monocotyledon
Silica Skeleton (ElongatePsilate)	1.2	0.5	
Cone	1.2	1.9	Cyperaceae (Sedge)
Hat Shaped	0.0	0.5	
Polylobate	9.6	4.8	Poaceae
Trapeziform	9.0	4.3	
Elongate Echinate	4.2	4.3	Poaceae (Inflorescence)
Elongate Dendritic	0.6	1.9	
Papillae	0.0	2.9	
Elongate Sinuate	3.6	1.9	Poaceae (Leaf/Culm)
Bulliform	4.2	5.7	
Bilobate Trapeziform	0.6	1.0	Poaceae (Panicoideae/Pooideae)
Rondel short cell	19.2	11.9	Poaceae (Pooideae C_3_ subfamily)
Crenate	0.0	1.9	
Saddle short cell	2.4	2.4	Poaceae (Chloridoideae C_4_ subfamily)
Phytolith Unidentified	6.6	8.6	Indeterminate

Attribution of phytolith morphotypes to plant taxa, plant parts and types of vegetation is shown.

Phytoliths from arboreal woody taxa, most probably dicotyledonous plants, were identified in both samples (16.2% in c1 and 20.5% in c2). Nevertheless we cannot disregard the presence of conifers, since some morphotypes such as blocky and tracheids may be found in both groups [56] (Table 6).

In general terms, both phytolith assemblages are very similar and show a predominance of grasses, especially of the *Pooideae* grass subfamily (C_3_) and to a lesser extent of the Chloridoideae grass subfamily (C_4_). Morphological identification also shows the presence of plants belonging to the Cyperaceae family, as well as woody plants (Dicotyledonous and possibly Coniferae). This arboreal presence is even more significant considering that phytolith production in grasses is substantially higher than in woody taxa (up to twenty times higher), especially in Mediterranean-Alpine environments [61, 96, 97].

### Macrovertebrates

A detailed taxonomic and taphonomic analysis of macrofaunal remains has only been possible for level c. Level b has yielded just 52 remains, of which only 8 could be identified as *Oryctolagus cuniculus*. Remains from level d, only excavated in square 3-R’, have been also included in this study, although they represent a very uninformative sample composed of few remains of herbivores and carnivores (Tables 7 and 8), where bear is the most represented species both in NISP and MNI.

**Table 7:** Taxonomical representation of Los Casares cave-Seno A faunal assemblages.

Level	C					D	
	1960’s	2014–2015	All				
	NISP	NISP	NISP	%	% partial	NISP	%
*Rhinocerontidae*	17		17	1,3	6,7		
*Bos / Bison*	9		9	0,7	3,5	2	2,4
*Equus ferus*	42	1	43	3,3	16,9	1	1,2
*Equus hydruntinus*	3		3	0,2	1,2		
*Cervus elaphus*	36	4	40	3	15,7		
*Dama sp*	1		1	0,1	0,4		
*Capreolus capreolus*	1		1	0,1	0,4		
*Capra pyrenaica*	85	14	99	7,5	38,8	3	3,5
*Rupicapra rupicapra*	32		32	2,4	12,5		
*Sus scrofa*	10		10	0,8	3,9		
*Ursus spelaeus*	20	2	22	1,7	19,3	32	37,6
*Crocuta sp*	24	8	32	2,4	28,1		
*Canis lupus*	12	4	16	1,2	14	1	1,2
*Panthera pardus*	4		4	0,3	3,5	6	7,1
*Lynx pardinus*	5	2	7	0,5	6,1		
*Felix silvestris*	10	1	11	0,8	9,6		
*Cuon alpinus*	3	1	4	0,3	3,5		
*Meles meles*	0	1	1	0,1	0,9		
*Vulpes Vulpes*	13	4	17	1,3	14,9		
Carnivore indet.	5	3	8	0,6			
Castor fiber	4		4	0,3			
Oryctolagus cuniculus	105	151	256	19,4		5	5,9
Birds indet.	6		6	0,5			
Indet. large size	15	32	47	3,6		8	9,4
Indet. medium size	1	3	4	0,3			
Indet. small Size	6	56	62	4,7		2	2,4
Indetermined	46	516	562	42,6		25	29,4
Total	515	803	1318			85	

Faunal assemblages from level c include remains from both old (1960’s) and recent (2014–2015) excavations. “% partial” refers to total of carnivores or herbivores.

**Table 8:** Taxonomical representation of Los Casares cave-Seno A according to MNI.

Level	c				d	
	MNI	MNI	%	% Partial	MNI	%
	A/J/I				A/J/I	
*Rhinocerontidae*	1	1	2.1	4.3		
*Bos / Bison*	1/1/0	2	4.2	8.7	1	12.5
*Equus ferus*	2	2	4.2	8.7	1	12.5
*Equus hydruntinus*	1	1	2.1	4.3		
*Cervus elaphus*	3/1/0	4	8.3	17.4		
*Dama sp*	1	1	2.1	4.3		
*Capreolus capreolus*	1	1	2.1	4.3		
*Capra pyrenaica*	6/1/0	7	14.6	30.4	1	12.5
*Rupicapra rupicapra*	2/1/0	3	6.3	13		
*Sus scrofa*	1	1	2.1	4.3		
*Ursus spelaeus*	3/1/0	4	8.3	33.3	1/1/0	25.0
*Crocuta sp*	1	1	2.1	8.3		
*Canis lupus*	1	1	2.1	8.3	1	12.5
*Panthera pardus*	1	1	2.1	8.3	1	12.5
*Lynx pardinus*	1	1	2.1	8.3		
*Felix silvestris*	1	1	2.1	8.3		
*Cuon alpinus*	1	1	2.1	8.3		
*Meles meles*	1	1	2.1	8.3		
*Vulpes vulpes*	1	1	2.1	8.3		
*Castor fiber*	1	1	2.1			
*Oryctolagus cuniculus*	10	10	20.8		1	12.5
Aves indet.	2	2	4.2			
Indet. large size						
Indet. medium size						
Indet. small Size						
Indet						
Total		48			8	

Faunal assemblages from level c include remains from both 1960’s and recent excavations. “% partial” refers to total of carnivores or herbivores. A: Adult, J: Juvenile and prime adult; I: Infant.

At level c, the study of bone assemblages excavated in the 2014 and 2015 seasons has confirmed previous suggestions [19] pointing out a bias in the recording of bone fragments during the 1960’s excavations. The low presence of undetermined material in the latter compared to the new assemblages (Table 7) suggest that an artificial selection was made during the excavation process, most probably due to an absence of sediment wet-screening. However, the higher diversity of herbivores in the 1960’s assemblage (Table 7) is best explained by the larger size of the excavated area. Concerning carnivores, both assemblages show an equivalent abundant sample (Table 7). Together with a higher presence of cranial remains in the old assemblage (see below), this evidence suggests that data from the two assemblages should be presented separately (Table 7 and Table F in S1 Appendix). However, since it is clear that both assemblages come from the same stratigraphic context and they both present similar taxonomic and taphonomic profiles, these limited recording biases did not prevent us of considering results together.

Considering both assemblages from the 1960’s and recent excavations, level c has yielded more than 1,300 faunal remains and a minimum number of 48 individuals. It shows a high taxonomic diversity, with Iberian ibex as the most represented herbivore species, but also including large bovids, horses, wild asses, deers, roe deers, chamois and wild boars. Among carnivores, the most abundant groups are hyenids and ursids, but canids and felids are also well represented (Table 7). Considering MNI, animals typical of rocky environments, such as Iberian ibex and chamois, are the most relevant, followed by deer. As for carnivores, bear is the most represented with 4 individuals (Table 8). Mortality patterns show that adult individuals dominate the faunal assemblage, both for carnivores and herbivores. Infant or juvenile-prime adults individuals have only been identified for Iberian ibex, chamois, deer and large bovid (Table 8).

Skeletal profiles for level c are biased by the low number of remains recorded for most animal species, with only lagomorphs being above 100 remains. Cranial elements are the most represented, ant teeth sum up to more than 50% of the sample for all taxa (Tables D and E in S1 Appendix). This preeminence of cranial elements, including teeth, is more striking when considering only the 1960’s assemblage (Table F in S1 Appendix), and hence could be related to a recording bias during the old excavations, as described above.

Among lagomorphs, all anatomical portions are found, but metapodials and phalanges account for 50% of remains (Table D in S1 Appendix). Concerning level d, skeletal profiles, dominated by teeth, are not considered representative due to the low amount of available faunal remains (Table G in S1 Appendix).

Taphonomic analysis of level c depicts a well-preserved assemblage, but showing an important skeletal bias towards the denser bones, especially cranial remains (Tables D and E in S1 Appendix), probably related to the bias recording of the 1960’s fieldworks (Table F in S1 Appendix). Weathering is only slightly recorded in bone surfaces, and the incidence of biochemical alteration is documented in less than 15% of the bones. Trampling affects to 2.5% of the bones, while hydric modification as showed by polishing, abrasion or carbonates, has been documented in less than 5%. Regarding types of breakage, only 10% of bones larger than 30 mm shows dry pattern, while green fractures have been recorded in 20%. The remaining 70% of bones show indeterminate breakage patterns. Therefore, impact of these processes on the faunal representation is not relevant.

Carnivore action has been also recorded in level c, but not in a prominent way considering that bones recording tooth marks are scarce in most taxa (Table 9). However, it is very likely that carnivores were responsible for the disappearance of several osseous portions as showed by (1) the mentioned teeth marks, (2) the presence of corrosion marks caused by digestive processes in some lagomorph bones, (3) the relative high amount of carnivores in the assemblage and (4) the absence of axial bones, such as ribs and vertebrae, coupled with the predominance of dense bones, such as teeth or lower appendicular limb bones. In the bone assemblage of layer d no carnivore alterations have been observed.

**Table 9:** Main bone alterations documented in level c.

Level c	%CM	%TM
*Equus hydruntinus*	50	
*Cervus elaphus*		8
*Capra pyrenaica*	3.2	13
*Rupicapra rupicapra*		20
*Sus scropha*		50
*Ursus spelaeus*		10
*Canis lupus*		40
*Felix silvestris*		1.1
*Carnivore indet*		50
*Oryctolagus cuniculus*	0.4	5.2
Indet.large size	4.3	2.1
Indet. medium size		10
**Total bone remains**		3.2

CM: Cut marks, TM: tooth Marks.

Concerning human activity, although no percussion marks have been observed in layer c, a limited number of cut marks has been recorded on remains corresponding to Iberian ibex, rabbit and wild ass (Table 9). While these marks denote some kind of human action on some animal species, they are not abundant enough to propose any conclusion in terms of economic behavior or subsistence strategies. No evidence of human action has been recorded on the faunal assemblage of layer d.

In sum, faunal assemblages of Los Casares-Seno A can be considered as produced basically by carnivore action, and only sporadically by humans in layer c.

### Lithic assemblage

**Fig 26:**
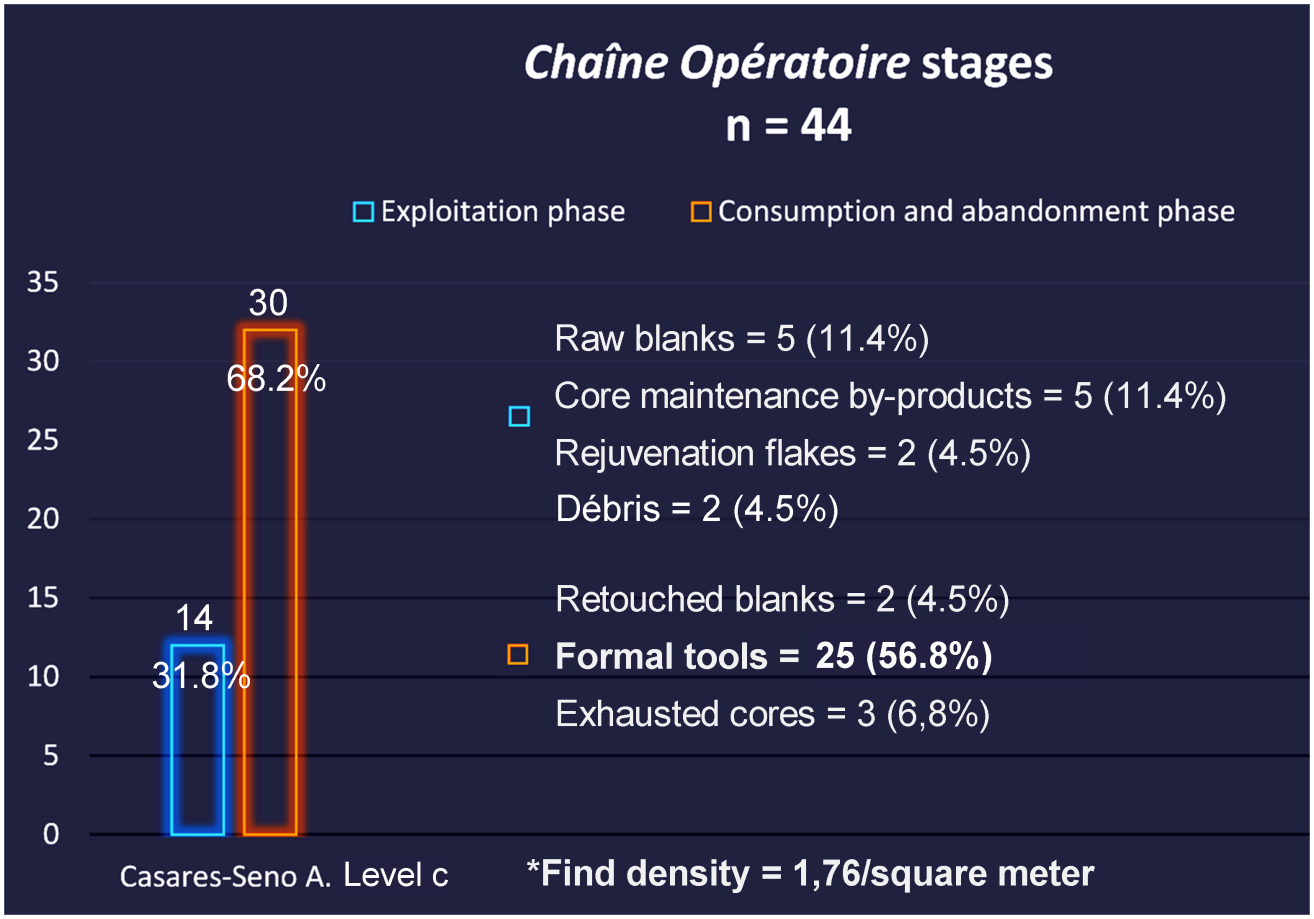
Mousterian lithic artefacts recorded in level c of Los Casares cave-Seno A according to the *chaîne opératoire stages*. Note that stage I (Initialization) is totally absent.

In Table 10 we show all technological categories described for the lithic assemblage of Los Casares-Seno A level c, and in Fig 26 we present the *chaîne opératoire* stages identified. Despite the low quantity of artefacts found at the site, it is noteworthy the high proportion of products corresponding to the consumption and abandonment stage (68.2%), being the rest assigned to the exploitation phase (31.8%). No elements have been related to the initialization phase. This predominance of consumption products is even higher when considering only artefacts made on flint (71.8%), while in the case of quartzite is significantly lower but still high (58.3%). Furthermore, most of these products are not simply retouched flakes, but formal tools, especially sidescrapers. All blanks are flakes except for one sidescraper on blade and one raw blade, both produced on flint.

**Table 10:** Technological categories with respect to lithic raw materials identified at level c of Los Casares cave-Seno A.

Technological categories	Flint	Quartzite	Total
Raw flake	4		4 (9.1%)
Raw blade	1		1 (2.3%)
*Débris*	1	1	2 (4.5%)
Core-maintenance by-product	1	4	5 (11.4%)
Rejuvenaton flake	2		2 (4.5%)
Retouched flake		2	2 (4.5%)
Sidescraper on flake	15	3	18 (40.9%)
Sidescraper on blade	1		1 (2.3%)
Retouched point	3		3 (6.8%)
Denticulate on flake	2		2 (4.5%)
Truncation on flake	1		1 (2.3%)
Exhausted Levallois core	1		1 (2.3%)
Core on flake		2	2 (4.5%)
**Total**	32 (72.7%)	12 (27.3%)	44 (100%)

**Fig 27:**
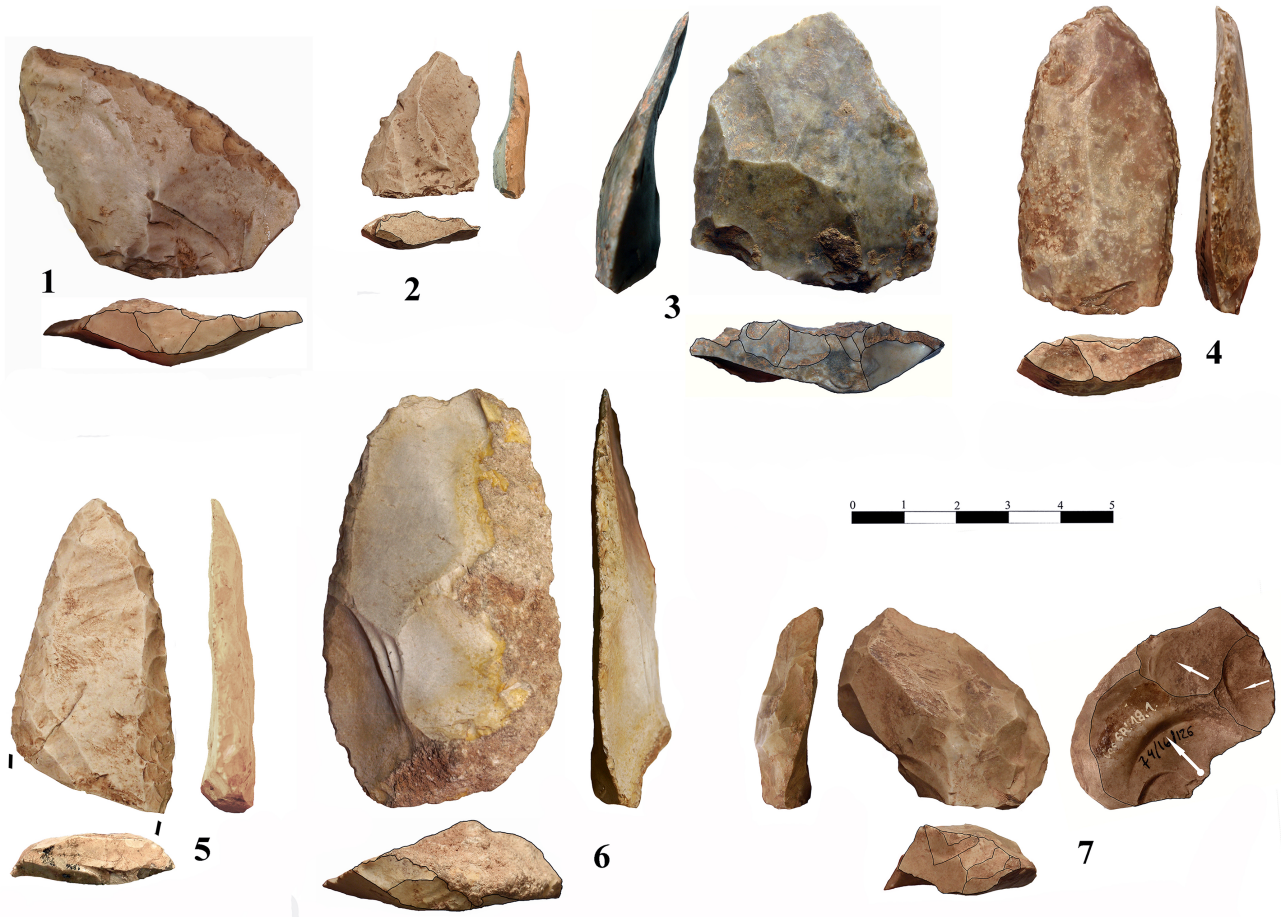
Mousterian lithic artefacts from level c of Los Casares cave-Seno A. Sidescrapers (1,3,4 6–7), denticulate (2) and point (5). All artefacts come from the 1960’s excavations (curated in the *Museo Arqueológico Nacional*, Madrid) except 3, which was recovered in our recent excavations. Item 7 is a sidescraper recycled into a core.

Retouched products, highly dominated by sidescrapers (Table 10), are mostly configured on blanks produced by recurrent centripetal Levallois methods as shown by centripetal scars on dorsal surfaces and facetted platforms (Fig 27). Some of these tools present evidences of recycling processes, such as exploitation of ventral surfaces, thus generating ‘core on tools’ pieces (Fig 27.7). A low number of small rejuvenation flakes produced during the resharpening of sidescrapers’ edges has been also found.

All these features suggest that the lithic assemblage preserved in level c of Los Casares-Seno A, undoubtedly reflecting a typical Middle Paleolithic technology, is mostly related to consumption activities. No knapping processes, besides some recycling or maintenance tasks, were developed at this part of the cave, which was perhaps focused on specialized activities as shown by the high presence of sidescrapers and other domestic tools.

Concerning level b, only one flint flake was recovered during fieldworks. Although this finding suggests that previous contentions that this layer was sterile [14] were probably wrong, it does not suffice to make any chrono-cultural assignment for it.

## Discussion

### Site formation processes

Micromorphological analyses have shown compelling evidence for site formation processes of the Seno A deposit. Level d0 represents differentially crystallised stalagmitic crusts and flowstones several cm thick and accumulated by chemical precipitation over a long period of time. Although the lateral continuity of the well-crystallised crust in profile 3-R’ is limited, level d0 represents a good stratigraphic marker for the unconsolidated overlying deposits.

Sediment composition and fabric of levels c and b suggest that they originate from an interplay of different transport and deposition processes within Seno A. Subsurface flow in the vadose zone of the cave system and possibly infiltration of fines through cracks has accumulated diverse carbonate and siliclastic mineral grains including well-rounded limestone boulders, siliceous gravel or phyllosilicate clay. In-situ remnants of water-laid deposits are preserved as intercalation of sand/fine gravel with silt/clay in level b2 of profile 8-W’. Although local concentration of well-rounded gravel, such as in layers b and cs4 of profile 1-O’, also reflect changes in flow velocity, these gravel do not appear in extended pockets or beds. Hence, in all layers except of b2, subaqueous deposition is not clearly indicated.

The contribution of roof-fall during accumulation of levels c to r was probably limited, because few angular to subangular limestone fragments were found. In the grey layers of level a3 limited roof-fall is included. The small rock fragments disintegrate leaving a clayey loam with reprecipitated calcite grains behind. This weathering product probably formed in water-filled basins of the cave floor.

During sediment accumulation of levels c, b0 and r, considerable zoogenic inputs of bat guano and carnivore coprolites occurred. In addition, bone constitutes a major component of most levels, but its origin may be related to both animals and humans. Charcoal is related to anthropogenic input, while the low numbers of charcoal in sediments from sediments below level a4 may at least partly be related to microbial degradation.

Postdepositional processes include corrosion and mechanical disintegration of limestone fragments and calcite grains. Carbonate leaching is indicated by local presence of undifferentiated b-fabrics, as well. Locally, heavily corroded carbonate grains are found in sand-size pores. Calcite pedofeatures including infillings and coatings indicate precipitation of secondary carbonates in several layers. This shows, that both partial leaching of carbonates and precipitation of secondary carbonates occurred, with stronger intensities in the lower part of the sequence, probably related to a longer period of time encompassed with sediments of level c. Besides calcitic pedofeatures, few other types of pedofeatures were detected. Locally, iron hydroxide or manganese oxide nodules, such as in level b0 of profile 8-W’ were found. Overall, few indicators of post-depositional mixing by sediment dwelling animals were detected in thin section.

A prominent feature in the studied thin section is the preservation of sediment boundaries showing accumulation of small charcoal fragments or manganese precipitates at the former surface or remnants of small sedimentary crusts of fine materials. The often high degree of compaction directly underneath these sediment interfaces or in whole layers clearly point to trampling effects during or after the accumulation of the layers. This is very obvious in sediments from the current cave floor down to level c1. The sequence thus clearly shows good preservation of layering, except of in its lower part (archeological level c) which neither shows clear evidence of mixing such as burrows nor of preservation of former surfaces, compacted parts or primary deposition by running water.

In sum, micromorphological features support the field distinction between dark or light grey sediments of levels r to a4, and the reddish-brown deposits of levels b0 to c. The sediment sequence in Seno A is well-stratified, particularly in the upper part down to above level c where remnants of several former trampled cave floors are preserved as indicated by characteristic sediment features. Mixing across boundaries between archeological levels was therefore very limited and hence the deposit can be considered as mostly *in situ*, at least in analyzed samples. The intensity of post-depositional changes including carbonate leaching and precipitation as well as precipitation of phosphate is higher in level c and b0 than in the upper levels, probably related to a longer time of exposure to this kind of diagenetic changes.

### Chronological and paleoenvironmental framework

Despite problems experienced with collagen-depleted bones, two independent chronometric methods place the Neandertal occupation of the Seno A within the middle-advanced stages of MIS 3. As the U-series ages obtained for layer d0 flowstone provide a *terminus post quem* of *c*. 48 ka BP (sample S3) for layer c, radiocarbon date of 44.9–42.2 ka cal BP obtained in this layer can be taken as a reliable approach to its age. This chronology, which is also consistent with biostratigraphic data provided by micromammal analysis, places the Middle Paleolithic occupation of Los Casares cave-Seno A within the final stages of the Neandertal presence in interior Iberia as currently documented. Although no reliable chronometric evidence is available for layer b, and its archeological content is uncertain and non-diagnostic, paleoenvironmental data gathered at this layer provide useful insights into its potential age and implications, as it will be discussed below.

Paleobotanical and microfaunal evidence presented in this study has substantially improved previous knowledge of the environmental and climatic framework where Los Casares’ Neandertals lived. Taken together, pollen, microvertebrates, charcoal and phyotlith data firmly point to a relatively temperate and humid interval within MIS 3 for level c. The presence of taxa such as *Acer*, *Tilia*, *Salix*, *Alnus*, *Pistacia terebinthus*, *Fraxinus* and deciduous *Quercus* in pollen samples collected in this level indicates a relatively forested Pyrenean oak landscape enriched in mesophilous trees and shrubs with some black pines [98]. The contention that central Iberia contained deciduous oak populations during glacial stages [99] is supported by our results, at least for MIS 3. In this sense, the study area can be considered as a glacial refuge for deciduous oaks and other Late Pleistocene temperate taxa, probably associated with higher water availability along river valleys, as has been reported for other nearby sites during MIS 2 [100].

Concerning the microvertebrates, the presence of forest-dwelling taxa, such as *Sciurus vulgaris* and *Apodemus*, Mediterranean species such as *Eliomys quercinus* and *Hystrix sp*., as well as species adapted to humid habitats such as *Castor Fiber*, *Arvicola sapidus* and *Iberomys cabrerae*, also suggest a warm and humid environment for level c [54, 101–102]. The absence of cold-indicator taxa in this level, such as the snow and tundra voles, is also of relevance here.

Evidence shown by proxies reflecting a more anthropogenic input into the site is in agreement with the pollen and microvertebrate results. Phytolith data gathered at level c also point to humid and warm environments, as shown by the high presence of Pooideae and Chloridoideae grass subfamilies and woody plants such as dicotyledonous, which are indicative of woodland landscapes and grassland or shrubs areas [59–60, 65, 96]. Although charcoal data have been limited to the presence of *Pinus* t. *sylvestris-nigra*, this is best described as evidence reflecting the trees supplying the fuel collected by Neandertals around the cave, as documented in many sites in Iberia during MIS 3 [103, 104].

Despite the scarcity of archeological or paleontological sites yielding paleoenvironmental data assigned to MIS 3 in interior Iberia, a good parallel for the paleoecological framework reconstructed at Los Casares can be found at Zarzamora Cave (Segovia). This site, very close to the northern foothills of the Central System range, is also dominated by *Quercus* and presents a micromammal assemblage reflecting temperate and humid conditions [105]. In the southern part of the Central System range, the MIS 5 site of Camino, in Pinilla del Valle [52, 106] also shows similar micromammal assemblages, albeit including some cold-indicators taxa which are absent in Los Casares-Seno A level c. Beyond the Meseta, but still in an interior region of Andalucía, microfaunal evidence from Carihuela cave (Granada) correlates well with Los Casares-Seno A assemblage, as reflected in the presence of *Allocricetus bursae*, the arvicolines *Iberomys cabrerae*, *Pliomys lenki* and the water vole *Arvicola sapidus* [107].

**Fig 28:**
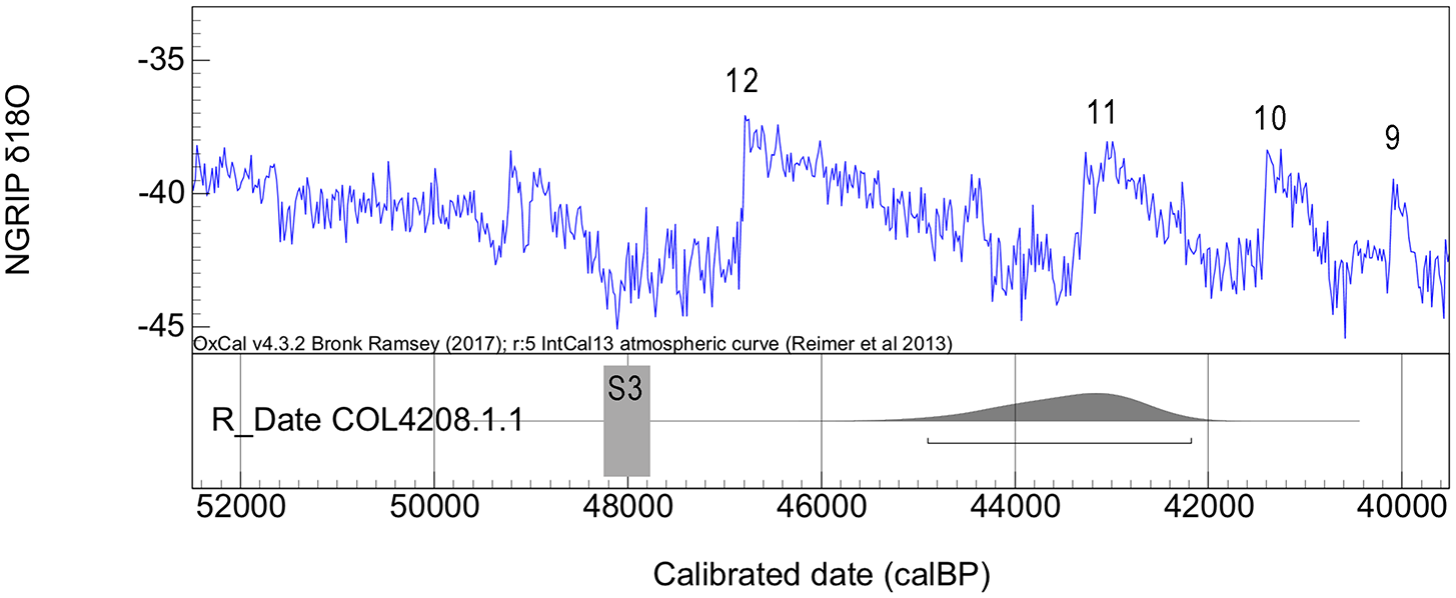
Correlation of radiocarbon calibrated date COL 4208.1.1 with Greenland Interstadials against the NGRIP δ18O record [111–112]. U/Th sample 3 is shown as a *terminus post quem* for the Middle Paleolithic layers.

Taking together paleoenvironmental and chronometric evidence, layer c of Los Casares cave-Seno A is most probably correlated with Greenland Interstadial 11, starting at 43.3 ka BP on the NGRIP δ18O timescale [108] (Fig 28). However, overlying layer b shows a very different paleoenvironment composition, pointing to a later phase probably correlated with subsequent stadial phases. LPAZ CS2, corresponding to level b, reflects a cold and arid climatic period dominated by *Pinus nigra*, evergreen *Quercus*, *Helianthemum*, *Juniperus*, *Cytisus*/*Genista*, Poaceae and *Artemisia*. It thus demonstrates the climatic variability within the MIS 3 in inland Iberia, and suggests the existence of relatively open black pine woodlands with some holm oak stands, grasslands and an abundant shrub cover of broom communities and juniper [109, 110]. This is consistent with microfaunal evidence, as seen in the reduction in the number of taxa identified in level b with respect to c. Also, the disappearance of forest-dwelling taxa that were present in level c, such as *Sciurus vulgaris* and *Apodemus* species, and of Mediterranean indicators such as the dormice, *Eliomys quercinus*, or the wood mouse, most probably record an increasingly colder climate in layer b. In this context, survival of species such as *Arvicola sapidus* and *Iberomys cabrerae*, both related to humid habitats [101], is best explained considering that they were probably less affected by climatic stress than the Woodland-Mediterranean indicators [54, 102].

Despite four radiocarbon attempts, there is no reliable chronometric evidence for layer b, which yielded only 52 macrofaunal remains and a single non-diagnostic flake. However, given chronometric results for levels c and d0, coupled with the paleoenvironmental and geochemical evidence described above, it is very likely that level b corresponds to a stadial phase following GI 11 as recorded in layer c. On this matter, the presence in layer b of the rodent species *Pliomys lenki*, which went extinct during the Late Pleistocene, points to a MIS 3 chronology. While the last appearance datum (LAD) of this species in northern Iberia is around 14 ka cal BP [113], its presence in the central and southern regions of the peninsula, although not radiometrically dated at the sites of Cova Negra [114] and Carihuela [115], suggest a LAD within MIS 3 [113]. Therefore, the most parsimonious interpretation is that layer b is of MIS 3 age, and most probably not much more recent than layer c, as also supported by sedimentological and geochemical data. Thus, GS 11, GS 10 or even Heinrich Stadial 4 (H4), spanning from *c*. 42 to 38 ka years ago [108, 116], are the most plausible correlations for layer b, and hence for a tentative phase of occupation at Los Casares reflecting a very scarce presence in the cave, at least for the interior area of Seno A. A subsequent hypothesis is that layer b indeed reflects the final stages of Neandertal presence in Los Casares, occurring sometime between *c*. 42 and 38 ka cal BP, and perhaps also the very last occupation of this high area of the Iberian interior due to climatic deterioration. However, given the scarce archeological content of layer b, some further reflections on this hypothesis will be made below.

## The last Neandertals of interior Iberia

Since the late 1980’s, the center and south of the Iberian Peninsula has been considered a sort of refuge where the last Neandertals persisted long after the first Modern Humans arrived to the north of the Peninsula and the rest of Europe [2, 23, 107, 117–124]. More recently, some authors have argued for a Neandertal survival south of the Ebro basin until at least *c*. 36.7–34.5 ka cal BP [5, 31], while others propose dates of *c*. 32–28 ka cal BP for the extreme southern regions of Iberia [4, 7]. Considering that dates for the appearance of the Proto-Aurignacian in the north of Iberia are well established around 42 ka cal BP [8, 125], a millennial coexistence between Neandertals and Modern Humans at the peninsular scale was accepted by most researchers until recently. However, in the very last years, new research focusing on the chronometric evidence [9, 28], and especially on new radiocarbon-dating projects based on ultra-filtration pretreatment of bone samples [6, 29], have questioned the late Neandertal survival model, thus supporting previous criticisms already raised by some scholars [126–128]. After refuting previously accepted late chronologies at the sites of Zarafarraya (Málaga) and Jarama VI (Guadalajara), and questioning the dates obtained in Gorham’s cave (Gibraltar), Carihuela (Granada), Gruta da Oliveira (Portugal) and Sima de las Palomas (Murcia), Wood et al. [6] have proposed a new probable scenario whereby Neandertal and Modern Human populations in Iberia did not co-exist and Middle Paleolithic sites do not occur after 42 ka cal BP. This is a relevant proposal, since it contradicts decades of acceptance of the late Neandertal survival hypothesis as the paradigmatic model.

However, the hypothesis of a not-so-late Neandertal population breakdown south of the Ebro basin has already received some criticism [26–27]. Both in the Mediterranean and Atlantic southern coasts of Iberia, some sites still suggest a post-42 ka cal BP chronology for the last Neandertal presence at the peninsula. Gorham’s cave (Gibraltar) [4, 7], Oliveira (Portugal) [25], Carihuela (Granada) [27, 129], Sima de las Palomas (Murcia) [130] and Cueva Antón (Murcia) [31] provide both chronometric and paleoecological data suggesting a persistence of Mousterian contexts after 42 ka cal BP. Despite the cases of Gorham and Carihuela have received strong criticism [6, 23, 131], dates obtained for Oliveira, Cueva Antón and Sima de las Palomas, although not without problems [6], remain unchallenged by means of new chronometric results. If these late survival cases are accepted, it would imply that Neandertals were present in the southern Iberian coasts at least until *c*. 37 ka cal BP, correlating with Greenland Interstadial 8. Since this chronology contradicts current trend suggested by the last chronometric investigations, research on this topic should be kept in the realm of hypothesis and theory building for now.

**Fig 29:**
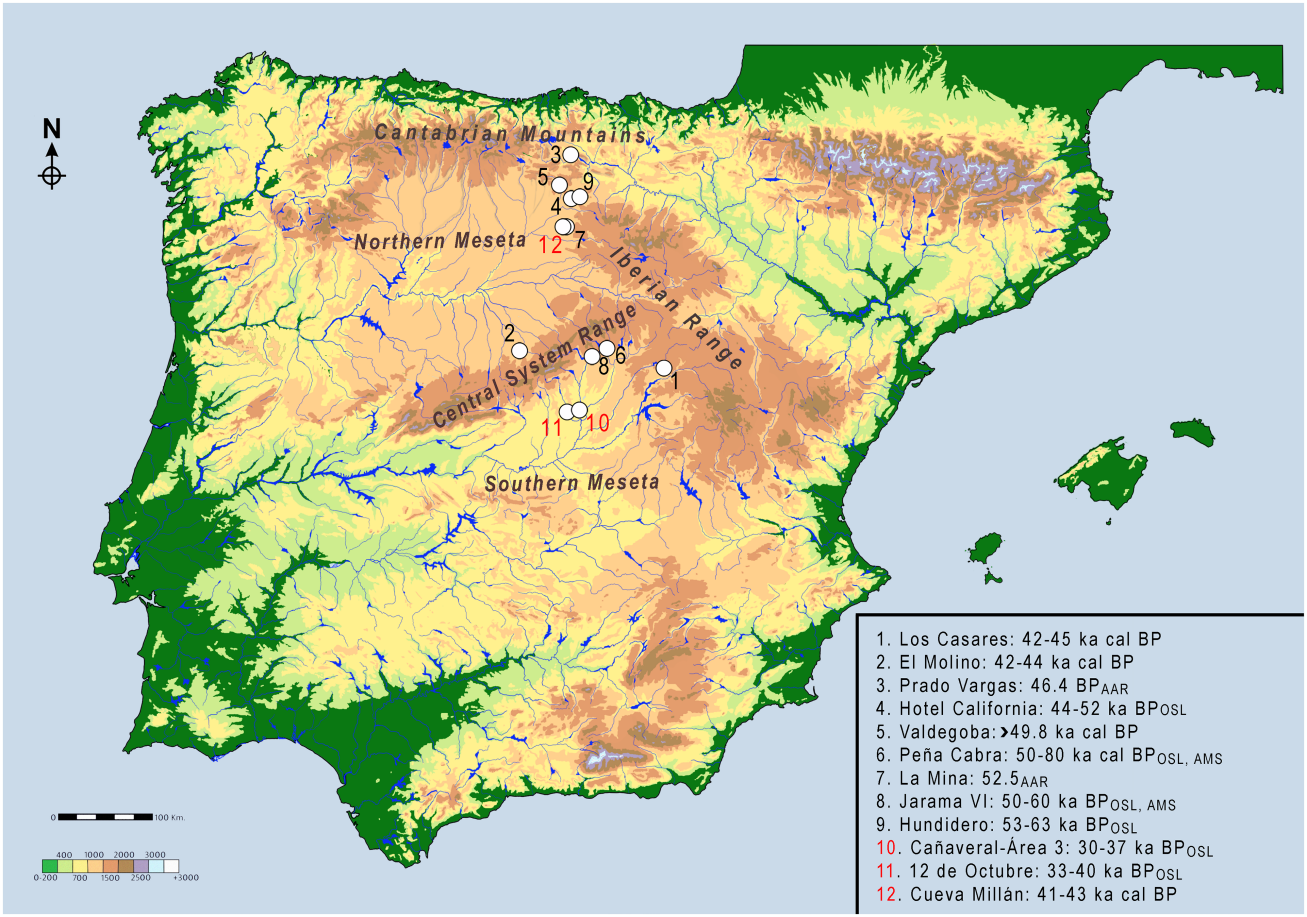
Middle Paleolithic sites in interior Iberia dated to MIS 3. Sites having yielded reliable chronometric dates are shown in black. Sites with uncertain results are numbered in red. For complete dating results and methods see [6, 9, 30, 132, 134–141, 145, 146]. Radiocarbon dates were calibrated using OxCal 4.3 [40] and IntCal13 [41]. OSL: Optically Stimulated Luminiscence. AAR: Aminoacid Racemization. AMS: Accelerator mass spectrometry.

Considering the Iberian interior territories, the strongest evidences supporting a late Neandertal survival have been unquestionably refuted. At La Ermita cave (Burgos), dates obtained by Aminoacid Racemization and Uranium/Thorium techniques have reassigned level 5a to MIS 5 [132], previously radiocarbon dated in the range of c. 36.6–34.7 ka cal BP [133]. At Jarama VI rockshelter (Guadalajara), the latest Mousterian occupation, previously radiocarbon dated between c. 41 and 30 ka cal BP [124], have been re-dated by new chronometric analyses, including radiocarbon measurements of bone samples pre-treated with ultrafiltration [6] and luminescence dating (post-IR IRSL) of associated sediments [9], to between c. 60 and 50 ka cal BP. Other interior Middle Paleolithic sites having yielded reliable chronometric dates within MIS 3 are Abrigo del Molino [134, 135], Prado Vargas [136], Hotel California [137], Valdegoba [138], Peña Cabra [139], La Mina [140] and Hundidero [141]. Since none of these sites have provided any date younger than 42 ka cal BP (Fig 29), the hypothesis of a not-so-late breakdown of Neandertal populations in Iberia remains unchallenged in the interior regions of the peninsula.

However, an important shortcoming faced by any study dealing with population dynamics in the Late Pleistocene of interior Iberia is the poor quantity and quality of the geoarcheological, paleoenvironmental and chronometric data available. This issue has been acknowledged in recent chronometric research [6, 9], and is most probably due to (1) a lack of research projects in interior Iberia compared to the coastal regions, and (2) the difficulties of locating open-air sites, potentially much more common than cave archives in the Spanish Meseta [16, 142–144]. In fact, there are three sites that could still suggest a post-42 ka cal BP chronology for Middle Paleolithic contexts in the Spanish plateau. In Cueva Millán (Burgos), two radiocarbon dates on bone obtained in the 1980’s ranged from *c*. 41 to 43 ka cal BP [140, 145]. However, these measurements were obtained by the conventional radiocarbon method, and hence a new chronometric program is required before the proposed dates can be considered to be reliable. In the Madrid basin, open-air sites of 12 de Octubre and Cañaveral-Área 3 have produced luminescence dates younger than 40 ka BP. In the 12 de Octubre deposit, a typical Mousterian assemblage is associated to a series of OSL dates between 40 and 33 ka BP. However, the excavators of this site cast doubt on these results suggesting that the proposed dates, which contradict geomorphological data, are most probably underestimates [146]. As for Cañaveral-Área 3, a TL date of 33 + 4.0/-3.5 ka BP was obtained at the top of a layer containing Levallois industries [30]. However, in addition to the high standard deviation of this measurement, and the fact that the date must be considered a *terminus ante quem* for human activity, a full discussion of methods and results of chronometric research conducted at this site is still to be published.

In short, although some uncertainties must be acknowledged when dealing with the Iberian interior territories, no strong chronometric evidence supporting a post-42 ka cal BP survival can be currently attested in them. In fact, Neandertals occupying the deep interior of Los Casares cave at *c*. 44.9–42.2 ka cal BP, must be considered among the last of their kind living in the interior lands of the Iberian Peninsula prior to their final disappearance (Fig 29). This evidence does not support a late survival of Neandertals in the Iberian interior, but rather suggests a not-so-late disappearance of this human group from these territories, roughly coincident with the proposed chronology for this process in northern Iberia [6, 8, 125].

However, although limited to a single flake and 52 faunal remains with no signs of human action, evidence gathered from layer b of Los Casares-Seno A must be also considered in this discussion. Paleoenvironmental data recorded in this layer show a cold and arid environment most probably correlating with GS 11, GS 10 or H4 (*c*. 42–38 ka years ago), thus suggesting a possible late and scarce presence of Neandertals at the cave. Since this could be interpreted as reflecting the near-abandonment of this high area (˃1,000 m asl) of the Iberian interior due to climatic deterioration, it could even be hypothesized that this layer also correlates with the final disappearance of this human species from inland Iberia due to climatic stress. Yet, since this is a hypothesis based on scarce empirical evidence, it cannot be used to support a late survival of Neandertals in interior Iberia. Given the still poor record gathered at this layer, and in general the scarce data available for discussing human-environment interactions in the Iberian interior during the Late Pleistocene, these reflections should be taken as working hypotheses to be tested with future research.

In any case, independent of whether layer b represents a late Neandertal presence at Los Casares or just an arid and cold episode devoid of human occupation (a question that remains open given that only one flake was recorded at this layer), the current record in interior Iberia shows a pattern in which little or no evidence for a Middle Paleolithic presence is registered after 42 ka cal BP. If we accept the late persistence of Neandertals in at least some of the southern coastal sites that are currently claimed to reflect Middle Paleolithic occupations until at least *c*. 37 ka years ago, a parsimonious corollary is that populations living in the highlands of the Spanish Meseta abandoned these potentially risky environments [147] and moved to the coastal areas of southern Iberia during some of the cold stadials following GI 11. The exact timing of this potential population movement is a question that needs further research. Notwithstanding, since no Upper Paleolithic occupations have been attested in inland Iberia until *c*. 25.5 ka cal BP [148], no action by Modern Humans could be invoked as triggering or even affecting this process. The breakdown of Neandertal populations in the Iberian interior is best explained as an abandonment of the area due to climatic deterioration or some other internal factor. This suggest that climate change could have been an important factor contributing to the final demise of the Neandertals [147, 149–152].

## Final remarks

Los Casares cave is a classic site for the study of the Middle Paleolithic settlement of inland Iberia. Despite its relevance in the last quarter of the 20^th^ century, data on this site was of little use for current research due to a prolonged period of scientific inactivity. New stratigraphic, micromorphological, chronometric, paleoenvironmental, archeozoological and technological data provided in this study have changed this situation. Los Casares cave has emerged as a relevant multi-proxy archive for studying human-environment interactions and population dynamics at the end of the Middle Paleolithic in the Iberian interior. Evidence discussed in this paper supports a breakdown of the Neandertal settlement system in inland Iberia around 42 ka cal BP or slightly later, and suggests that this could be related to an abandonment of the interior highlands of the Meseta due to climate deterioration. The last Neandertals of Iberia are thus only found in the southern coastal areas of the peninsula, where a post-42 ka cal BP survival of Middle Paleolithic contexts has not been falsified. Although evidence discussed in this paper represents a significant advance on these topics, the geoarcheological, paleoecological and chronometric record in the Iberian interior are still too weak to allow for theory building at the regional level, despite significant progress in the recent past. It is our contention that further fieldwork on the under-investigated interior regions of the Iberian Peninsula will substantially change–again–models on population dynamics in Iberia and southwest Europe during this critical period of human prehistory. Until then, unbiased data gathering and hypothesis testing remain crucial.

On epistemic grounds, far from the classic Kuhnian scenario of rapid and definitive paradigmatic shift–which is rarely verified–, it is our contention that the current scientific situation on the problem of Neandertal disappearance in Iberia should be best considered as a not-so-fast process of data accumulation and hypotheses proposal that should eventually lead to a new big picture on the issue. Whether this picture will be totally different to previously accepted one, slightly different, or even in consonance, is a question that remains open despite great advances in the last years. Only more fieldwork (including excavation of new sites), data gathering (not only chronometric, but also stratigraphic, paleoenvironmental and archeological), and problem-oriented research, will eventually lead to still not definitive, but increasingly better, scientific answers. Ongoing investigations in a handful of sites in the interior regions of Iberia, albeit limited to the foothills of the Central System range, the Madrid basin and the Atapuerca area, will hopefully contribute to that end [135, 139, 144, 153, 154].

## Supporting information

S1 Appendix Supporting tables on micromorphology and archeozoology and taphonomy. (PDF)
