# Supplementary material for: A context for the last Neandertals of interior Iberia: Los Casares cave revisited
Source: PLoS One. 2017 Jul 19;12(7):e0180823. doi: 10.1371/journal.pone.0180823 (PMC5516997; doi:10.1371/journal.pone.0180823)
Supplement: S1 Appendix — (PDF) [file pone.0180823.s001.pdf]

Supporting Information

A Context for the Last Neandertals of Interior Iberia: Los Casares Cave Revisited

Manuel Alcaraz-Castaño*, Javier Alcolea-González, Martin Kehl, Rosa-María Albert, Javier Baena-Preysler, Rodrigo de Balbín-Behrmann, Felipe Cuartero, Gloria Cuenca-Bescós, Fernando Jiménez-Barredo, José-Antonio López-Sáez, Raquel Piqué, David Rodríguez-Antón, José Yravedra, Gerd-Christian Weniger

* Corresponding author. Email: manuel.alcaraz@uah.es (MAC)

S1 Appendix

Supporting Tables (Micromorphology)

Table A. Micromorphological description of thin sections from the south profile of square 3-R'.

Table B. Micromorphological description of thin sections from monolith 4 from the south profile of square 1-O'.

Table C. Micromorphological description of thin sections from monolith 5 from the south profile of square 8-W'.

Supporting Tables (Archeozoology and taphonomy)

Table D. Skeletal profiles (NISP) of level c for herbivores.

Table E. Skeletal profiles (NISP) of level c for carnivores.

Table F. Skeletal sections of bones for level c.

Table G. Skeletal profiles (NISP) of level d.

Table A. Micromorphological description of thin sections from the south profile of square 3-R'.

Arch. level	Sub-level	TS	Color, texture	Coarse materials	Micromass*	Voids, microstructure (ms), packing density (pd); notes on fabric	Upper boundary	Pedofeatures	Remarks
r		1.1/2.1	dark brown sandy loam, very few small gravel	Abundant medium to coarse silt and sand grains of carbonates, quartz, limestone, siltstone; many pieces of bone and charcoal; many small (<1mm) bat and some lager carnivore coprolites containing silt grains and few vesicular pores	Ssp to dsp; cryst;	Few chambers and burrows (planes*); massive and pt. fine subangular blocky ms; high pd	Dense		Rich in anthropogenic compounds, compaction by trampling, weak bioturbation
a3	a3s1	1.1/2.1	light grey silty clay loam, many gravel	Dominated by carbonate silt and sand and limestone gravel, very few quartz; very small pieces of charcoal	Op; cryst	Few burrows, (planes*); massive ms; very high pd; microlaminated aggregates inclined at diverse angles	Sharp	Few, small typic Fe-hydroxide nodules, few incomplete infillings of micrite	Limited mechanical disintegration and corrosion of limestone gravel, compaction by trampling
	a3s2	1.1/2.1	yellowish brown silty clay loam, few gravel	Dominated by carbonate fines but including fine gravel and coarse sand of siltstone as well as siliceous fines; limestone rock frags; few charcoal	Op; cryst	Many small vughs (planes*); massive partly spongy ms; high pd in the upper part, moderate density below	Sharp		More intense corrosion of limestone gravel than above, compaction by trampling
a4		1.1/2.1	light and dark grey silty clay loam, many small gravel	Limestone gravel intermixed with charcoal and very few coarse sand grains of siltstone	Cp; n.a.	Many compound packing voids and vughs; vughy ms; moderate pd	Clear	Few incomplete infillings of micrite	Accumulation of charcoal at former surface, no clear compaction by trampling
b0		1.1/2.1	brown sandy loam, few	Abundant medium to coarse silt and sand grains of quartz and	Cp to ssp;	Few planes and vughs; massive, partly	Sharp		Erosional contact with overlying strata,

			gravel	carbonate; smaller grains mainly consist of carbonate and siliceous clay; few gravel of quartzite and limestone; many bone and charcoal fragments; some bat coprolites; very few gravel size carnivore coprolites	st-sp	subangular blocky ms; high pd			compaction by trampling, weak bioturbation
b	bs1	1.1/2.1 to 1.2/2.2	brown sandy loam, few gravel	Similar to b0, but many bone fragments of fine gravel size	Cp to ssp; st-sp	Vughs and complex packing voids; massive and granular ms; moderate to high pd; textural coatings on large grains	Sharp		Compaction by trampling at surface, rolling of rounded grains and mixing probably by bioturbation and recompaction
	bs2	1.2/2.2	brown sandy loam, few gravel	Similar to above, but less bone and coprolites	Cp to ssp; st-sp	Complex packing voids; granular ms; low pd; textural coatings on large grains	Diffuse		Similar to above, but without surface layer; mixing was not followed by compaction
c1	c1s1	1.2/2.2	brown sandy loam, slightly darker than above, few gravel	Similar to bs2, some large and many small bone fragments, very few pieces of charcoal	Cp to ssp; st-sp	Few planes and vughs, occasional chambers; massiv ms; high pd	Sharp	Vertical burrow	Deposit rich in small bone frags, compaction by trampling, very limited bioturbation
	c1s2	1.2/2.2	brown sandy loam, few gravel	Slightly better granulometric sorting than above with lower amounts of coarse fragments and bone, no charcoal	Cp to ssp; st-sp	Few vughs and planes; massive ms, high pd; several microlayers indicated by differential degree of compaction	Sharp	Vertical burrow	Internal sediment interfaces indicate several events of trampling, limited bioturbation after burial by c1s1
c2		1.2/2.2 to 1.3/2.3	brown sandy loam, few gravel	Similar to C1.1, less well sorted than layer above; many carnivore coprolites	Cp to ssp; st-sp	Vughs, complex packing voids, chambers, planes; massive pt. granular	Clear	P in limestone rims; P infillings in large pores; incomplete microsparitic and	Strong corrosion of limestone fragments, local bioturbation, phosphatization of

ms; moderate to high pd	micritic calcite infillings and typic calcite nodules; vertical burrows	groundmass increasing with depth, calcite precipitation, limited bioturbation

Key: TS - thin section; * Coarse-fine related distribution pattern: clp - closed porphyric, ssp - single spaced porphyric, dsp - double spaced porphyric, op - open porphyric; birefringence fabric: crys - crystallitic, undiff. - undifferentiated, st-sp - stipple- speckled, mo-sp - mosaic-speckled, gra-str - grano-striated.

1) For description of micromass see text.

* Planes probably represent artefacts due to drying

Table B. Micromorphological description of thin sections from monolith 4 from the south profile of square 1-O'.

Arch. level	Sub-level	TS	Color, texture	Coarse materials	Micromass*	Voids; microstructure (ms); packing density (pd)	Upper boundary	Pedofeatures	Remarks
a4	a4s1	4/1	dark brown silty clay loam	Few silt- and sand-size carbonate and quartz grains, many large charcoal frags, few light colored bone and many small orange bone frags, well sorted microlayer of siliceous silt and clay including fine pieces of charcoal, few small orange coprolites	Dsp; st-sp	Few vughs, massive ms (*), high pd	Dense	-	Compaction due to trampling
	a4s2	4/1	dark brown sandy loam, very few gravel	Few sand-size carbonate and quartz grains; many charcoal frags and very few coarse sand grains of siltstone; small orange coprolites	Dsp; st-sp	Few vughs, massive ms (*), moderate pd	Sharp	-	

b0		4/1	yellowish brown sandy loam, few gravel	Many sand-size carbonate and quartz grains; many rounded sand-size coprolites, locally arranged in a banded fabric, some pieces of small and large bone; no charcoal; quartzite gravel	Dsp; st-sp to mo-sp	Few vughs, massive ms, high pd	Clear	Complete P infillings	Strong accumulation of phosphate, no clear equivalent to b in profile 3R
b		4/1	grey brown to brown sandy loam, abundant gravel	Many sand-size carbonate and quartz grains; many limestone and one quartzite gravel, few to many bone frags and coprolites increasing in number with depth	Cp; st-sp	Few vughs, massive locally vughy ms; moderate pd	Diffuse	Sparitic incomplete calcite infillings	Limited, locally heavy corrosion of limestone gravel
c	cs1	4/1; 4/2	brown sandy loam, few large gravel	Many carbonate and quartz sand grains; few gravel; many coprolites; less bone than in layer above	Cp to ssp; st-sp	Few vughs, massive locally vughy ms; moderate pd	Diffuse	Few thin clay coatings	
	cs2	4/2	light brown sandy loam, many gravel	Many carbonate and quartz sand grains; many limestone and one quartzite gravel; many bones frags, and large coprolites	Cp to ssp; st-sp	Many vughs; vughy locally spongy ms; low pd	Diffuse	Incomplete P infillings	
	cs3	4/2; 4/3	brown sandy loam, few gravel	Few carbonate but many quartz sand grains; few gravel; many small coprolites, few bone	Ssp to dsp; st-sp to mo-sp	Many vughs; vughy ms; moderate pd	Diffuse	Many dissolution pores, pt including highly corroded remnants of calcite crystals	Fe-mottling, high degree of carbonate depletion
	cs4	4/3	light brown sandy loam, many gravel	Many limestone and few quartzite gravel; moderate amounts of coprolites and bone	Cp to ssp; st-sp	Few vughs, vughy ms, low pd	Diffuse	Incomplete P infillings	

Key: TS - thin section; * Coarse-fine related distribution pattern: clp - closed porphyric, ssp - single spaced porphyric, dsp - double spaced porphyric, op - open porphyric; birefringence fabric: crys - crystallitic, undiff. - undifferentiated, st-sp - stipple- speckled, mo-sp - mosaic-speckled, gra-str - grano-striated.

* Planes and complex packing voids as well as angular blocky microstructure due to shrinking,

Table C. Micromorphological description of thin sections from monolith 5 from the north profile of 8-W'.

Arch. level	Sub-level	TS	Color, texture	Coarse materials	Micromass*	Voids, microstructure (ms), packing density (pd); notes on fabric	Upper boundary	Pedofeatures	Remarks on site formation processes
r	rs1	5/1	Grey silty clay loam	Many quartz silt and sand grains; small pieces of charcoal, many small and few large coprolites	Op; st-sp	Few vughs; massive ms; high pd	Dense	P infillings near the top	Compaction by trampling
	rs2	5/1	Brown sandy loam	Abundant silt and sand grains of quartz, many pieces of bone and charcoal; many small and few large coprolites;	Op; st-sp	Many vughs and planes; vughy to (sub)angular blocky ms; moderate pd	Clear		Similar to r in profile 3R, but no carbonate grains and not compacted
a3		5/1	Light grey silty clay loam, few gravel	Dominated by carbonate fines but including fine gravel and coarse sand of siltstone as well as siliceous fines; comparatively few limestone rock frags; much charcoal	Op; cryst	Few vughs and planes; massive ms; high pd	Sharp	Many P infillings in the upper 5 mm	Similar to a3s1 in profile 3R, but gravel often consist of schist and more pieces of charcoal
a4		5/1	Orange brown to black sandy loam, very few gravel	Abundant silt and sand grains of quartz, many pieces of bone and charcoal; many small orange and few large coprolites; much charcoal and amorphous black matter (probably Mn) at the lower boundary;	Dsp; st-sp;	Few to many vughs, few complex packing voids and planes; vughy ms; moderate pd	Sharp		No equivalent in profile 3R

b0		5/1	Orange brown sandy loam	Silt and sand size quartz grains; many small coprolites	Dsp to op; undiff. to st-sp	Few vughs and (sub)horizontal planes; massive ms; high pd	Clear	P infillings in elongate horizontal pores; Fe hydroxide and typical Mn oxide nodules	Laminar fabric, equivalent to b in profile 2?
b1	b1s1	5/1	Brown sandy loam	Silt and sand grains of quartz; Few bone	Ssp to dsp; undiff. to st-sp	Many vughs and complex packing voids; vughy partly granular ms; low to moderate pd	Diffuse	P infillings in elongate horizontal pores	
	b1s2	5/2	Brown sandy loam, very few gravel	Many limestone and few quartzite gravel; moderate amounts of small and large coprolites, few bone	Ssp to dsp; undiff. pt. gra-str;	Same as above	Diffuse		Corrosion of limestone rock frags, Clay-rich fragmented microlayer at top
b2		5/2	Intercalation of grey sand and gravel with brown sandy loam on top of silty clay loam	Sandy with quartzite and siltstone gravel	Ssp to op; undiff. to mo-sp;	Many vughs, few vertical planes; vughy ms; low pd	Clear		Erosional upper contact, no equivalent in profile 1 and 2
c		5/2, 5/3	Brown sandy loam, many gravel	Many carbonate and few quartz sand grains; many limestone and few quartzite gravel; moderate amounts of small and large coprolites, few bone	Clp to ssp; undiff., pt. gra-str	Many complex packing voids; granular ms; low pd	Sharp	Calcite coatings on larger grains	Corrosion of carbonate grains near the top of the layer; rich in carbonate sand

Key: * Coarse-fine related distribution pattern: clp - closed porphyric, ssp - single spaced porphyric, dsp - double spaced porphyric, op - open porphyric; birefringence fabric: crys - crystallitic, undiff. - undifferentiated, st-sp - stipple- speckled, mo-sp - mosaic-speckled, gra-str - grano-striated.

Table D. Skeletal profiles (NISP) of level c for herbivores.

**Level c**	***Diceror.***		***Equus***		***Equus hyd.***		***Bos***		***Cervus***		***Capra***		***Rupicapra***		***Sus scrop.***		***Castor***		***Oryctolag.***	
	NISP	*%*	*NISP*	*%*	*NISP*	*%*	*NISP*	*%*	*NISP*	*%*	*NISP*	*%*	*NISP*	*%*	*NISP*	*%*	*NISP*	*%*	*NISP*	*%*
**Horn**									2	5			1	3						
**Cranial**			1	2																
**Mandible**			1	2					1	3	1	1							7	3
**Maxillar**											1	1	1	3					2	1
**Teeth**	15	88	35	81	1	33	8	91	25	63	69	69	21	66	5	50	4	100	25	10
**Vertebrae**									1	3	1	1	1	3					1	0
**Rib**																			1	0
**Scapula**																			2	1
**Sternon**																			1	0
**Humerus**									1	3	1	1			2	20			9	4
**Ulna**											2	2							8	3
**Radius**											3	3							7	3
**Metacarpal**			1	2							1	1							3	1
**Carpal**	1	6	1	2					1	3	5	5	1	3						0
**Pelvis**																			9	4
**Femur**													1	3					7	3
**Tibiae**							1	9	1	3									16	6
**Talus**			2	5					2	5										0
**Metapodial**																			52	20
**Metatarsal**			1	2					2	5	2	2							2	1
**Calcaneus**									1	3									28	11
**Phalange**	1	6	1	2	2	67			3	8	13	13	6	19	3	30			76	30
Total	17		43		3		9		40		99		32		10		4		256	

Table E. Skeletal profiles (NISP) of level c for carnivores.

**Level c**	**Carniv. Indet.**		***Cuon***		***Felix***		***Crocuta***		***Panthera p.***		***Canis l.***		***Lynx p.***		***Meles m.***		***Ursus***		***Vulpes***	
	NISP	*%*	*NISP*	*%*	*NISP*	*%*	*NISP*	*%*	*NISP*	*%*	*NISP*	*%*	*NISP*	*%*	*NISP*	*%*	*NISP*	*%*	*NISP*	*%*
**Mandible**			1	25	1	9											1	5		
**Teeth**	2	50	2	50	2	18	28	88	3	75	11	69	5	71	1	100	12	55	9	53
**Vertebrae**	1	25				0											2	9		
**Rib**	1	25				0											1	5		
**Humerus**					1	9													1	6
**Radius**			1	25		0														
**Metacarpal**						0					1	6								
**Carpal**						0											2	9		
**Tibiae**						0													1	6
**Metapodial**						0			1	25	2	13					2	9	1	6
**Metatarsal**						0													2	12
**Calcaneus**					4	36	1	3												
**Phalange**					3		3	9			2	13	2	29			2	9	3	18
**Total**	4		4		11		32		4		16		7		1		22		17	

Table F. Skeletal sections of bones for level c.

	**1960's**	**2014-2015**
**Level c**	%NISP	%NISP
Axial	4	3
Cranial	58	17
Shaft	10	57
Epiph.	6	3
Compact.	22	20
NISP	515	803

"Compact" include talus, carpal, tarsal and phalange. "1960's" shows old excavation data while "2014-2015" shows recent data.

Table G. Skeletal profiles (NISP) of level d.

**Level d**	***Bos***		***Capra***		***Equus***		***Oryctolagus***		***Ursus***		***Panthhera pardus***		***Canis l.***		**Small size**	
	NISP	*%*	*NISP*	*%*	*NISP*	*%*	*NISP*	*%*	*NISP*	*%*	*NISP*	*%*	*NISP*	*%*	*NISP*	*%*
**Mandible**							2	40	2	6						
**Teeth**			2	67					11	34	4	67	1	100		
**Vertebrae**									8	25						
**Rib**									2	6						
**Humerus**	2	100					1	20	1	3						
**Radius**									1	3						
**Pelvis**															1	50
**Femur**									1	3						
**Phalange**									4	13					1	50
**Tibiae**							1	20								
**Talus**			1	33	1	100			1	3	2	33				
**Metapodial**							1	20	1	3						
**Total**	1		2		1		5		32		6		1		2	
